# One-step propylene purification from a quaternary mixture by a single physisorbent

**DOI:** 10.1038/s41467-025-66438-9

**Published:** 2025-12-13

**Authors:** Peixin Zhang, Zhensong Qiu, Yechen Liu, Sen Chen, Lifeng Yang, Xian Suo, Xili Cui, Huabin Xing

**Affiliations:** 1 Zhejiang Key Laboratory of Intelligent Manufacturing for Functional Chemicals, College of Chemical and Biological Engineering, Zhejiang University, Hangzhou, China; 2 Engineering Research Center of Functional Materials Intelligent Manufacturing of Zhejiang Province, Institute for Intelligent Bio/Chem Manufacturing, ZJU-Hangzhou Global Scientific and Technological Innovation Center, Hangzhou, China; 3 State Key Laboratory of Silicon Materials, School of Materials Science and Engineering, Zhejiang University, Hangzhou, China

**Keywords:** Chemical engineering, Organic-inorganic nanostructures

## Abstract

One-step removal of multiple impurities implemented by adsorptive separation is an efficient and simple process to afford high purity products, but is hindered by the lack of advanced porous materials that could capture different types of molecules. Herein, a series of novel metal-organic frameworks ZU-921 to ZU-924 with cooperative binding environment of integrated aromaticity surface and fluorine/oxygen electronegative sites are designed, and ZU-921 is presented as the demonstration that solves the long-standing challenge in one-step propylene (C_3_H_6_) purification from the C3 quaternary mixture. The selective recognition ability towards alkyne, allene, alkane than alkene implemented by ZU-921 is attributed to the optimal interaction contribution from polarizability and dipole/quadruple moments that is realized by the fine-tuned density of parallelly-distributed electronegative sites via ligand engineering strategy. Ultra-high purity (99.99%) C_3_H_6_ could be directly obtained from the C3 quaternary mixture (C_3_H_4_/C_3_H_4_(PD)/C_3_H_8_/C_3_H_6_ 1 v/1 v/3 v/95 v) with the productivity of 17.27 L/kg derived from the 10-times scale-up column (1.0 cm × 50 cm) breakthrough experiment. This work not only presents a common strategy in advanced adsorbents design for multiple impurities capture but also provides an energy-efficient alternative for C_3_H_6_ purification.

## Abstract (web summary)

Researchers developed a porous material ZU-921 that solves a key challenge in removing multiple different types of impurities through fine-tuning pore chemistry, enabling the one-step production of high-purity propylene.

## Introduction

Multiple impurity capture is the essential step in gas purification, determining the gas purity, and is closely associated with the energy cost of the process^1–5^. Polymer-grade (>99.5%) propylene (C_3_H_6_) is the critical bulk commodity, and its purification involves the removal of complex impurities with similar properties, like propyne (C_3_H_4_, ~1%), propadiene (C_3_H_4_ (PD), ~1%), and propane (C_3_H_8_, ~3%)^6,7^. Currently, the purification of alkene relies on the tandem separation process in industry, including catalytic hydrogenation (noble-metal catalyst at high temperature and pressure) and cryogenic distillation (over 100 trays at 243 K and 0.3 MPa). The energy-intensive nature of the above separation process has spurred research into the development of nonthermal-driven separation technology^8,9^.

Adsorptive separation is recognized as a potentially energy-saving alternative to solve challenging separations, and considerable achievements have been gained along with the continuous development of advanced porous materials^10,11^, like metal-organic frameworks (MOFs)^12–17^, covalent-organic frameworks (COFs)^18,19^, etc.^20–23^. Benefiting from their demonstrated fine-tuning ability of pore size and pore chemistry, tailor-made porous materials towards C_3_H_6_ purification have been developed, such as binary mixtures of C_3_H_4_/C_3_H_6_^24,25^, and C_3_H_6_/C_3_H_8_^26–28^, ternary mixtures of C_3_H_4_/C_3_H_4_(PD)/C_3_H_6_^29,30^. In detail, the designed porous materials, decorating the pore surface with polar groups, like anions^31^, open metal sites^32^, are able to preferentially adsorb the molecules with higher dipole/quadrupole moments, and remove trace alkyne from C_3_H_6_ mixtures, as well as the selective capture of C_3_H_6_ from C_3_H_8_. Constructing an alkane trap to introduce high-density weak interaction sites can enhance the binding affinity with C_3_H_8_, and realize the selective capture of C_3_H_8_ from C_3_H_6_ mixtures^33,34^, but fail to simultaneously capture the C_3_H_4_, C_3_H_4_(PD) with lower polarizability. Controlling pore size to create molecular sieves that could exclude molecules of large size, achieving the separation of C_3_H_4_ and C_3_H_6_ from C_3_H_4_/C_3_H_6_ and C_3_H_6_/C_3_H_8_ mixtures, respectively^35–38^. However, despite the above progress, one-step C_3_H_6_ purification from quaternary C3 mixtures containing C_3_H_4_, C_3_H_4_(PD), and C_3_H_8_ via a single physisorbent still remains a grand challenge^39^.

**Fig. 1:**
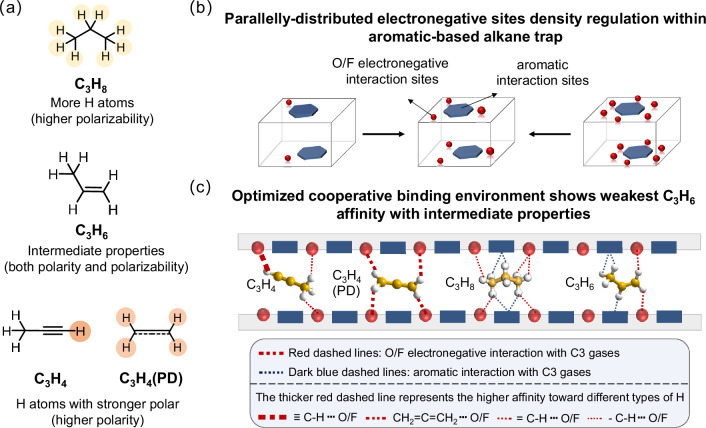
Schematic diagram of the adsorbent design and adsorption behavior for one-step C_3_H_6_ purification. **a** The properties difference of C_3_H_4_, C_3_H_4_ (PD), C_3_H_6,_ and C_3_H_8_, **b** the illustration of the strategy of adsorbents design. Introducing O/F electronegative sites into the aromatic-based propane trap to form a cooperative binding environment, and controlling the density of introduced electronegative sites to fine-tune the binding affinity sequence of the four C3 gases, **c** the adsorption behavior of C_3_H_4_, C_3_H_4_ (PD), C_3_H_6,_ and C_3_H_8_ under the optimal cooperative interaction environments. C_3_H_4_ and C_3_H_4_ (PD) exhibit higher affinity with O/F than C_3_H_6_ due to their higher polarity, while C_3_H_8_ could form more dense interactions with the aromatic-based sites and O/F sites than C_3_H_6_.

To realize one-step C_3_H_6_ purification, the adsorbents are expected to show higher binding affinity towards all C3 alkyne, allene, and alkane than alkene. However, as revealed by the physiochemical properties of the four gases, they have very similar properties, especially for C_3_H_6_ and C_3_H_8_. The polarizability, dipole/quadrupole moments, and molecular size of C_3_H_6_ all lie between C_3_H_4_/C_3_H_4_ (PD) and C_3_H_8_. Meanwhile, C_3_H_8_ shows the highest polarizability but the lowest dipole/quadrupole moments, while the condition is reversed for C_3_H_4_ and C_3_H_4_(PD). The fact indicates that the different binding sites or environments and their fine-tuning are required to realize the preferential accommodation of C_3_H_4_, C_3_H_4_(PD), and C_3_H_8_ than that of C_3_H_6_ in the confined channel, posing a daunting challenge to the design of porous materials (Fig. 1a and Fig. S1). In detail, to fulfill the target of simultaneous capture of C_3_H_4_, C_3_H_4_ (PD), and C_3_H_8_, the design of porous materials needs to overcome two great challenges. (1) Creating a molecular trap that shows preferential adsorption towards C_3_H_8_ than C_3_H_6_, the small polarizability difference between C_3_H_8_ and C_3_H_6_ makes it challenging (polarizability: C_3_H_8_ 62.9–63.7 × 10^−25^ cm^3^ vs C_3_H_6_ 62.6 × 10^−25^ cm^3^)^33,34,40^, the C_3_H_8_/C_3_H_6_ selectivity of most reported C_3_H_8_-selective materials is around 1.5^41,42^. (2) Creating the cooperative binding environment that recognizes molecules via both polarizability and dipole/quadrupole moments^43,44^. Different affinity sequences of the four C3 gases could be realized via fine-tuning the interaction contribution from the different kinds of binding sites^32^ (Fig. 1b, c). Through the exploitation of more H interaction sites of C_3_H_8_ than C_3_H_6_ and higher electropositivity H atoms of C_3_H_4_, C_3_H_4_(PD) than C_3_H_6_, porous materials with an optimal cooperative binding environment rationally show the weakest C_3_H_6_ affinity. Following this idea, the fluorine/oxygen polar binding sites are integrated into the aromatic-based alkane trap that is mainly dominated by the dispersion/induction interactions based on polarizability.

Through controlling the density of parallelly distributed fluorine via a ligand engineering strategy, the contribution of interactions from the dipole/quadrupole moments and polarizability could be fine-tuned. Herein, we solved the challenge of one-step C_3_H_6_ purification from the quaternary mixtures using tailor-made ZU-921 (ZU-represents Zhejiang University) featuring orderly lined aromatic surfaces and optimal density of electronegative sites. The selectively adsorbed alkyne, allene, and alkane over alkene molecules are bonded simultaneously via hydrogen-bonding interactions and multiple van der Waals interactions with high selectivity of alkane/alkene (2.03), alkyne/alkene (2.17), and allene/alkene (2.03). High-purity (99.99%) C_3_H_6_ could be directly obtained from C3 quaternary mixtures (C_3_H_4_/C_3_H_4_(PD)/C_3_H_8_/C_3_H_6_ 1 v/1 v/3 v/95) with the productivity of around 17.27 L/kg using ZU-921 as demonstrated by the 10-times scale-up column breakthrough experiments. The molecular-level understanding of the adsorption behavior for C3 gases within the well-defined pore space highlighted the importance of the rational integration of synergistic binding sites to enhance the recognition ability of multiple gases with different properties.

## Results

### Synthesis and characterization

**Fig. 2:**
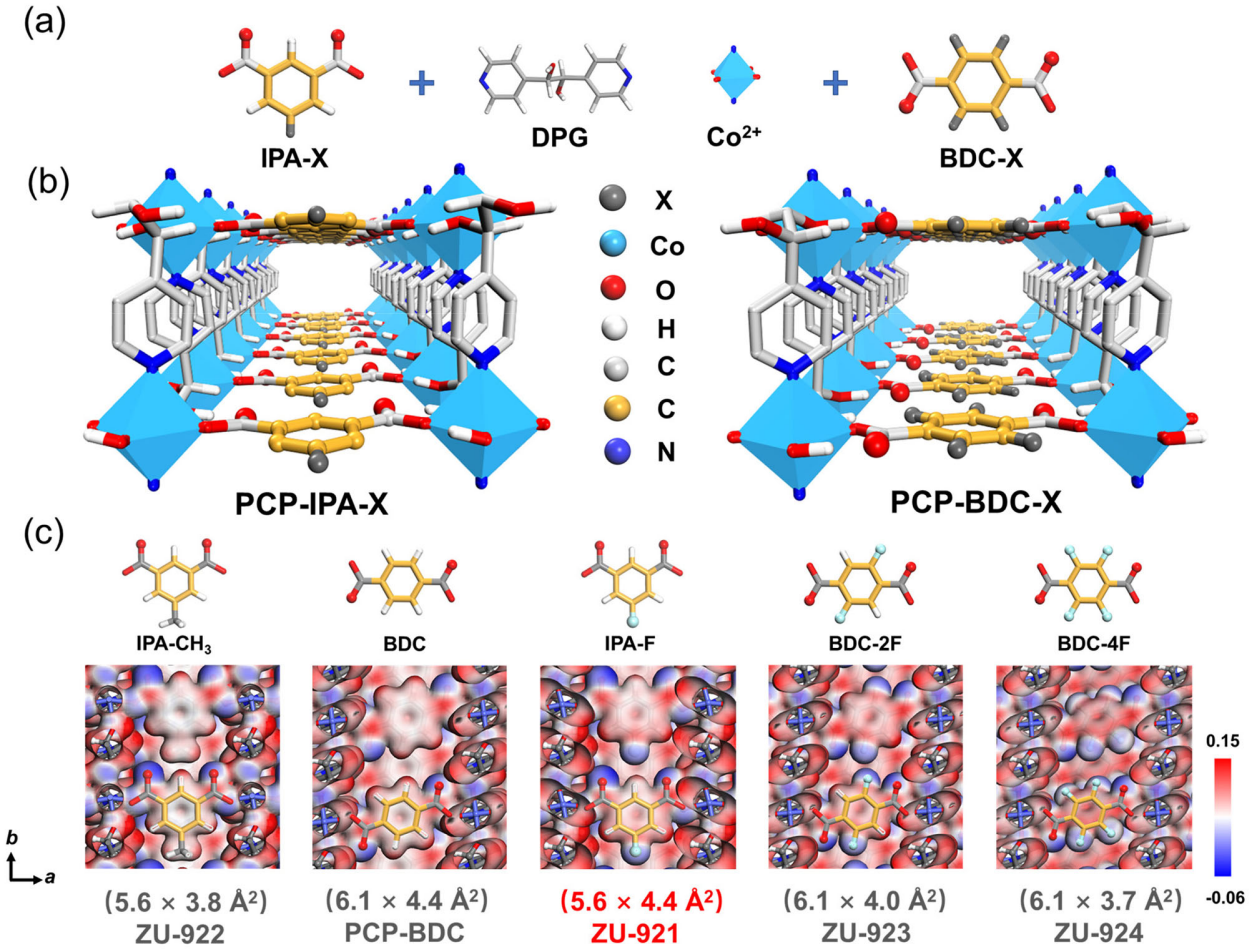
Scheme and structure of five isostructural MOFs. **a** The building blocks (Co^2+^, DPG, and organic ligand of IPA-X and BDC-X, X represents different functional groups), **b** the structure of the three-dimensional framework of PCP-IPA-X and PCP-BDC-X, and **c** the electrostatic potential maps of ZU-921 to ZU-924 and PCP-BDC and their pore size.

A series of isostructural ultramicroporous materials, ZU-921 ([Co(IPA-F)(DPG)]_n_, DPG = meso-α, β-di(4-pyridyl) glycol, IPA-F = 5-fluoroisophthalic acid), ZU-922 ([Co(IPA-CH_3_)(DPG)]_n_, IPA-CH_3_ = 5-methylisophthalic acid), ZU-923 ([Co(BDC-2F)(DPG)]_n_, BDC-2F = 2,5-difluoroterephthalic acid) and ZU-924 ([Co(BDC-4F)(DPG)]_n_, BDC-4F = tetrafluoroterephthalic acid) were successfully synthesized through solvothermal reactions of Co(NO_3_)_2_·6H_2_O, meso-α, β-di(4-pyridyl) glycol and their corresponding dicarboxylate acid. The structures of ZU-921 to ZU-924 are isostructural except for the functional groups, as revealed by their similar powder X-ray diffraction (PXRD) patterns (Fig. S2). The method of Rietveld refinement was adopted to obtain the refinement structures of ZU-921 and ZU-922 based on the parent structure of [Co(IPA)(DPG)]_n_ (IPA = isophthalic acid)^45^, and the low R_p_ (0.0143, 0.0142) and R_wp_ (0.0298, 0.0270) values indicate the high quality of the analyzed structures (Fig. S3, Table S2 and “Structure simulation” section). The PXRD patterns of the synthesized power of ZU-921 and ZU-922 are well matched with the simulated one of the refined structures (Fig. S4). Individually, each Co(II) atom was connected by two pyridine groups and two hydroxyl groups from independent meso-α, β-di(4-pyridyl) glycol ligands to form a 2D layer network, and the 2D layer network was further pillared by the dicarboxylate acid (IPA-F and IPA-CH_3_, BDC, BDC-2F and BDC-4F) to afford a 3D framework with one-dimensional straight channel (Fig. 2a, b). The pore windows of ZU-921 to ZU-924 are estimated to be 5.6 × 4.4 Å^2^, 5.6 × 3.8 Å^2^, 6.1 × 4.0 Å^2^, and 6.1 × 3.7 Å^2^ by the model of Connolly surface with probe of diameter 1.0 Å (Fig. 2b)^46^. The channels of these isostructural materials are featured with the parallel-aligned linearly extending isophthalic acid units, which can provide a big π system and multiple hydrogen bond acceptors for the accommodation of alkane. Through ligand engineering strategy, the surface electrostatic potential of the pore environment could be well fine-tuned via controlling the types and density of functional groups. We could observe that the pore channel shows more negative electrostatic potential with the increased density of polar fluorine functional sites, which would enhance the binding affinity with polar molecules (Fig. 2c). The permanent porosity of the synthesized porous materials was investigated by 77 K N_2_ and 195 K CO_2_ adsorption-desorption isotherms (Figs. S5 and S6). The corresponding surface area and pore volume were calculated and summarized in Table S3. The Langmuir surface area and pore volume were determined as 356.6 m^2^ g^−1^ and 0.145 cm^3 ^g^−1^ for ZU-921, and 369.5 m^2^ g^−1^ and 0.150 cm^3 ^g^−1^ for ZU-922, respectively. The pore size distribution (PSD) of ZU-921 and ZU-922 is centered at 4.9 Å and 4.5 Å, respectively, which agrees well with the theoretical pore size from crystal simulation (Figs. 1b and S6). Moreover, thermogravimetric analysis (TGA) curves demonstrated the good thermal stability of ZU-921 and ZU-922, and their decomposition temperatures are up to 280 °C and 300 °C, respectively (Fig. S7). The morphology was investigated using a NOVA 200 Nanolab scanning electron microscope (SEM) (Fig. S8). Additionally, the invariable PXRD patterns and BET results indicate that ZU-921 is stable in different solvents (MeOH, EtOH, MeCN, Acetone, and DMF) and solutions with different pH (pH = 5, pH = 9, pH = 11) (Figs. S9–S11).

### Adsorption and separation performances

**Fig. 3:**
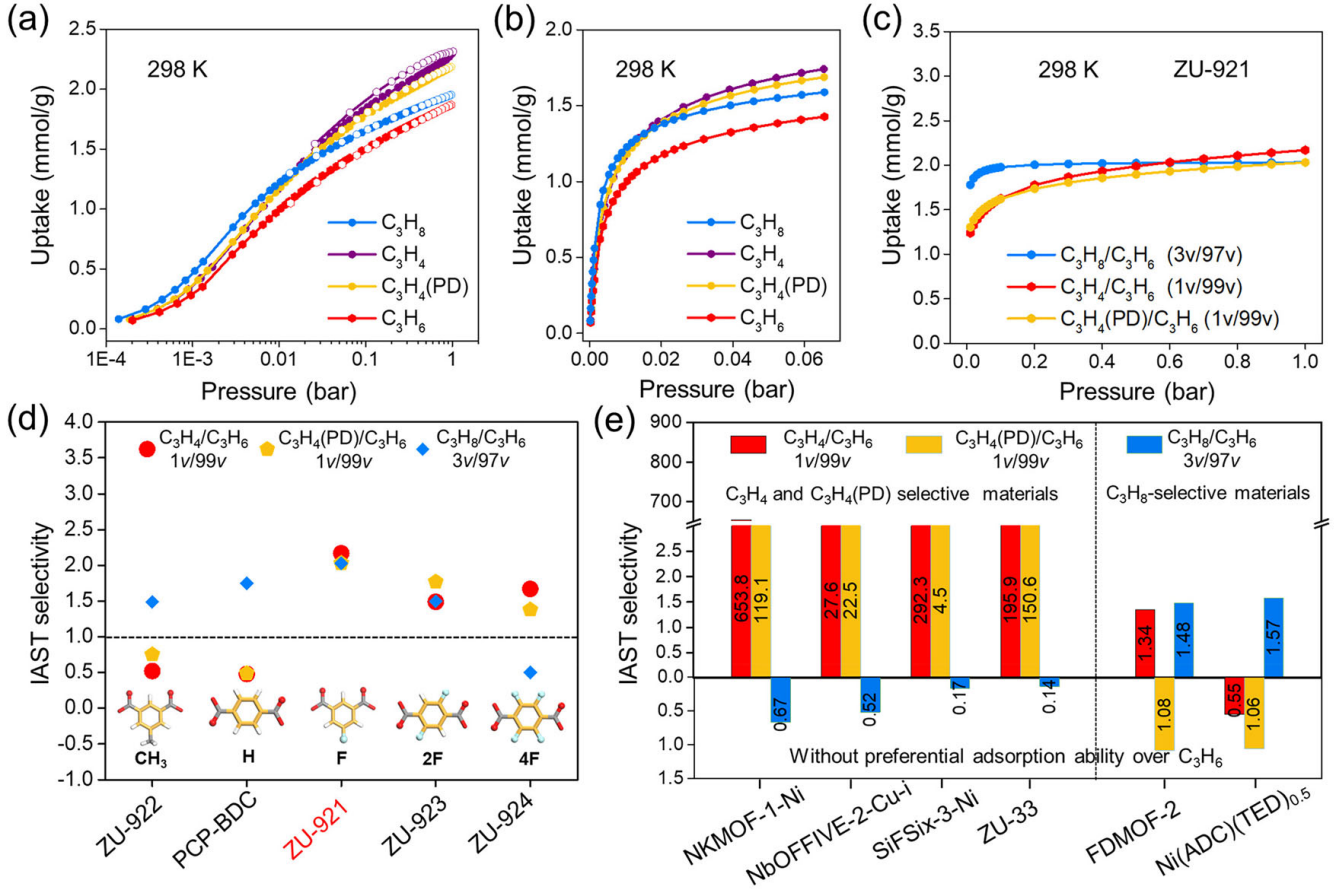
Adsorption and separation performance. The C_3_H_4_, C_3_H_4_(PD), C_3_H_8_, and C_3_H_6_ adsorption isotherms of ZU-921 at 298 K with (**a**) a logarithm scale under the pressure range of 0–1.0 bar, (**b**) a linear scale under the pressure range of 0–0.06 bar, **c** the IAST selectivity of C3 binary mixtures on ZU-921, **d** comparison of the IAST selectivity of C3 binary mixtures for the series of ZU-921 to ZU-924 and PCP-BDC at 298 K, and **e** comparison of the IAST selectivity of different C3 binary mixtures on ZU-921 with reported benchmark materials for C3 separation. Source data are provided as a Source Data file.

The single-component adsorption isotherms of C_3_H_4_, C_3_H_4_(PD), C_3_H_6_, and C_3_H_8_ were measured to explore the adsorption performance of ZU-921 to ZU-924 and PCP-BDC (Figs. 3a, b and S12–S17). As depicted in Fig. 3a, b, all the C_3_H_8_, C_3_H_4_ (PD), and C_3_H_4_ adsorption capacities of F-functional ZU-921 are higher than that of C_3_H_6_ during the whole pressure range (0–1.0 bar), suggesting its preferential adsorption behavior towards C_3_H_4_, C_3_H_4_(PD), and C_3_H_8_ over C_3_H_6_. To our knowledge, this adsorption behavior has not been observed in the reported literature. The ideal adsorbed solution theory (IAST) method is applied to further evaluate the separation potentials of porous materials^6^. Considering the impurity content of C_3_H_4_, C_3_H_4_ (PD), and C_3_H_8_ in cracking gas typically account for 0.5–1%, 0.5–1% and 2–3%^29,34,47^, respectively, the selectivity of C_3_H_8_/C_3_H_6_ (3 v/97 v), C_3_H_4_/C_3_H_6_ (1 v/99 v) and C_3_H_4_(PD)/C_3_H_6_ (1 v/99 v) binary mixtures on ZU-921 to ZU-924 and PCP-BDC were calculated (Fig. S18). As shown in Fig. 3c, ZU-921 exhibited high IAST selectivity for all of C_3_H_8_/C_3_H_6_ (3 v/97 v), C_3_H_4_/C_3_H_6_ (1 v/99 v), and C_3_H_4_(PD)/C_3_H_6_ (1 v/99 v) binary mixture at 298 K and 1.0 bar, which is up to 2.03, 2.17, and 2.03, respectively. Relatively, ZU-922 and PCP-BDC with aromatic-based pore environment exhibited moderate C_3_H_8_/C_3_H_6_ selectivity (1.49 and 1.75) but failed to selectively capture polar C_3_H_4_ and C_3_H_4_ (PD). ZU-924 with a high density of polar F-functional sites shows the priority adsorption sequence of C_3_H_4_ > C_3_H_4_ (PD) > C_3_H_6_ > C_3_H_8_ (Figs. 3d and S18). The experimental results demonstrate that the introduced density of the polar F-functional group is critical to afford the ideal materials for one-step C_3_H_6_ purification. The time-dependent adsorption curves demonstrate that the adsorption rates of all four C3 gases in ZU-921 are close with no obvious kinetic effect, indicating that the good C_3_H_6_ purification performance of ZU-921 is governed by the thermodynamic equilibrium mechanism (Figs. S20–S22).

Furthermore, we conducted a detailed comparison study about the adsorption separation performance for four C3 gases of the literature-reported benchmark materials, for example, the C_3_H_4_ and C_3_H_4_(PD)-selective materials (NKMOF-1-Ni^30^, NbOFFIVE-2-Cu-i^29^, SIFSIX-3-Ni^24^, and ZU-33 (GeFSIX-14-Cu-i)^47^) and C_3_H_8_-selective materials (Ni(ADC)(TED)_0.5_^48^ and FDMOF-2^34^). The C_3_H_4_, C_3_H_4_ (PD), C_3_H_6_, and C_3_H_8_ adsorption isotherms of these materials and their corresponding IAST selectivity of binary mixtures were measured and calculated (Figs. S23–S29 and Tables S4 andS5). None of these materials are able to simultaneously capture C_3_H_4_, C_3_H_4_ (PD), and C_3_H_8_ from C_3_H_6_ mixtures (Fig. 3e). In detail, the C_3_H_4_ and C_3_H_4_(PD)-selective materials with polar sites exhibit outstanding selectivities of C_3_H_4_/C_3_H_6_ and C_3_H_4_(PD)/C_3_H_6_, but the C_3_H_8_/C_3_H_6_ selectivity is lower than 1.0, indicating that the polar sites used to capture polar alkyne and allene are not beneficial for the alkane-selective adsorption. Similarly, the C_3_H_8_-selective materials with high-density weak interaction sites fail to capture C_3_H_4_ and C_3_H_4_(PD), and the C_3_H_8_/C_3_H_6_ selectivity is always low (≤2.0), revealing that the inert pore environment designed for the C_3_H_8_-selective adsorption could not be adapted for the accommodation of polar alkyne and allene. These results indicated that it was of great challenge to construct advanced porous materials that could selectively adsorb polar alkyne and allene, and inert alkane over alkene.

### Transient breakthrough experiments

**Fig. 4:**
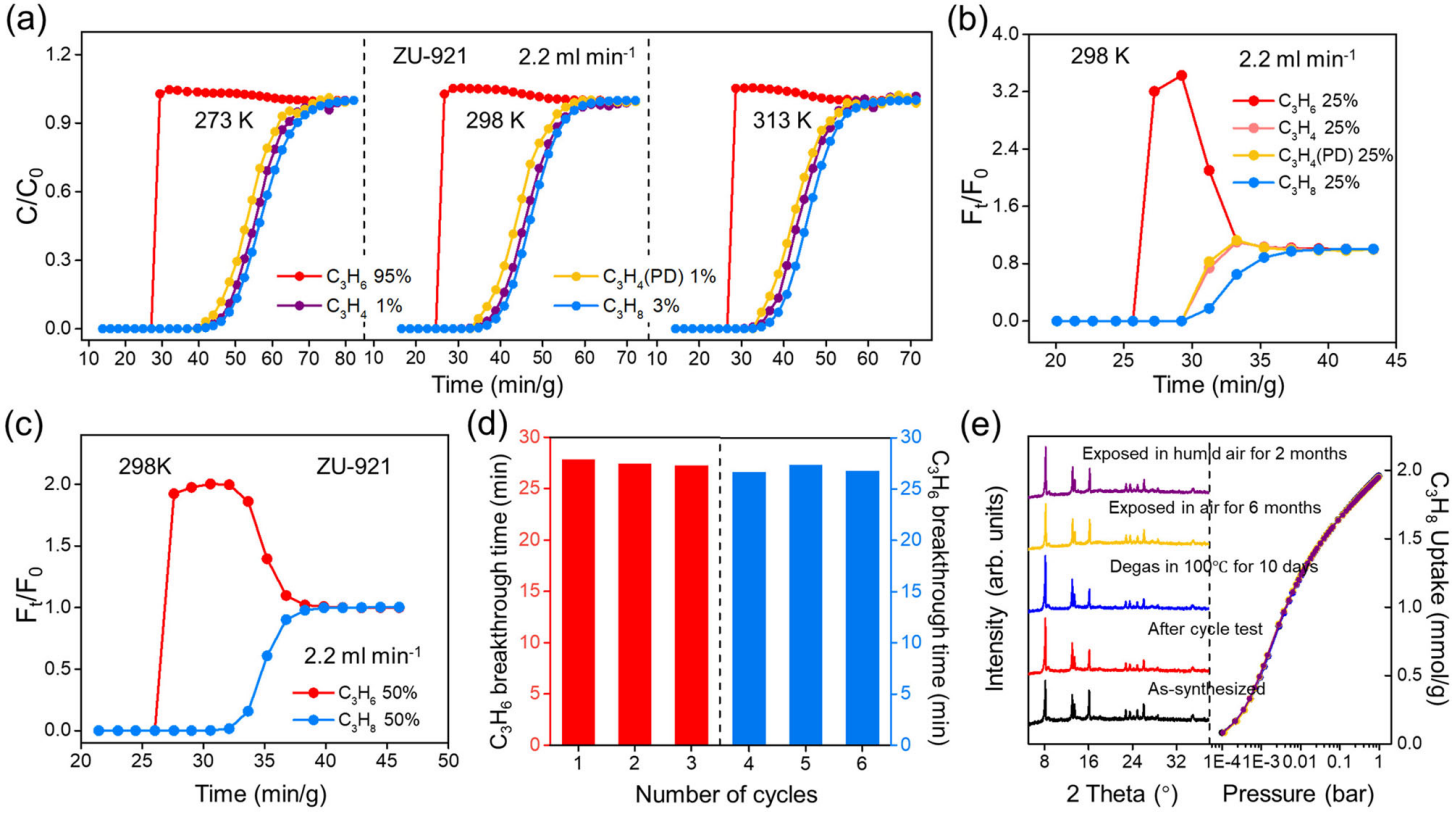
Olefin purification. Dynamic breakthrough curves of ZU-921 for (**a**) C_3_H_4_/C_3_H_4_(PD)/C_3_H_8_/C_3_H_6_ (1/1/3/95 *v/v/v/v*) mixture in C_t_/C_0_ under 273, 298 K and 313 K and 1.0 bar; **b** C_3_H_4_/C_3_H_4_(PD)/C_3_H_8_/C_3_H_6_ (25/25/25/25 *v/v/v/v*) mixture in F_t_/F_0_ under 298 K and 1.0 bar; **c** C_3_H_8_/C_3_H_6_ (50/50 *v/v*) mixture in F_t_/F_0_ under 298 K and 1.0 bar; **d** Six recycling breakthrough tests for C_3_H_8_/C_3_H_6_ (50/50 *v/v*, red) and C_3_H_4_/C_3_H_4_(PD)/C_3_H_8_/C_3_H_6_ (1/1/3/95 *v/v/v/v*, light blue) separation with ZU-921 under 298 K and 1.0 bar; **e** The PXRD patterns and C_3_H_8_ adsorption isotherms of ZU-921 after different treatments. (column: 0.46 cm × 15 cm, 1.33 g or 0.46 cm × 25 cm, 2.28 g, flow rate: 2.2 mL min^−1^). Source data are provided as a Source Data file.

Inspired by the unique adsorption behavior and high separation selectivity for C3 binary mixtures of ZU-921, the dynamic breakthrough experiments with mimicking C3 quaternary mixture proportions of cracking gas (C_3_H_4_/C_3_H_4_(PD)/C_3_H_8_/C_3_H_6_ 1 v/1 v/3 v/95 v) were conducted to evaluate its actual separation ability. The detailed experiment conditions and calculation methods of C_3_H_6_ productivity were described in the supporting information (Figs. S30–S31 and Table S13). As described in Fig. 4a, as the C_3_H_4_/C_3_H_4_(PD)/C_3_H_8_/C_3_H_6_ (1 v/1 v/3 v/95 v) mixture flowed through the column packed with ZU-921 under different temperatures (273 K, 298 K and 313 K), the C_3_H_6_ always eluted first, and then C_3_H_4_, C_3_H_4_(PD) and C_3_H_8_ broke out simultaneously, indicating good one-pot C_3_H_6_ purification performances of ZU-921. Specifically, 15.21 L/kg (99.99%) of C_3_H_6_ could be produced directly at 298 K and 1.0 bar (Fig. S32). The simple C_3_H_6_ purification process provides a potential energy-saving route to replace the current complex cascade purification ways. In addition, the separation performance towards equimolar C3 quaternary mixture (C_3_H_4_/C_3_H_4_(PD)/C_3_H_8_/C_3_H_6_ 25/25/25/25 v/v/v/v) on ZU-921 was further explored, and the C_3_H_6_ still firstly eluted at the time of 26.5 min/g with the C_3_H_6_ (99.5%) productivity of 3.63 L/kg (Figs. 4b and S33 andS34). The roll-up phenomenon of C_3_H_6_ indicates that ZU-921 shows the weakest affinity towards C_3_H_6_, and all C_3_H_4_, C_3_H_4_(PD), and C_3_H_8_ were almost simultaneously eluted, demonstrating their close binding affinity within ZU-921 (Fig. 4b). We also explored the dynamic breakthrough performance of ZU-922 and FDMOF-2 under the same conditions. As shown in Figs. S35 and S36, C_3_H_6_, C_3_H_4_, and C_3_H_4_(PD) were simultaneously eluted, while C_3_H_8_, with the highest adsorption affinity, was well adsorbed. The results showed that ZU-922 and FDMOF-2 could only realize the selective C_3_H_8_ capture from C_3_H_6_, consistent with the adsorption isotherm results. Considering the complex competitive behavior in multi-component separations, the breakthrough experiments for binary mixtures of C_3_H_8_/C_3_H_6_ and C_3_H_4_/C_3_H_6_ were conducted (Figs. S37 andS38). As revealed by the breakthrough experiments of C_3_H_8_/C_3_H_6_ and C_3_H_4_/C_3_H_6_ binary mixtures, C_3_H_6_ is always first eluted, followed by C_3_H_8_ or C_3_H_4_, indicating that ZU-921 could selectively capture the C_3_H_4_ and C_3_H_8_ to produce C_3_H_6_ directly. The C_3_H_6_ productivity of ZU-921 is up to 10.08 L/kg for the C_3_H_8_/C_3_H_6_ (50/50) binary mixture (Figs. 4c and S40), lower than FDMOF-2 (21.0 L/kg)^34^, but exceeding most of the reported materials, BUT-10 (3.95 L/kg)^43^ and WOFOUR-1-Ni (3.50 L/kg)^44^ under the same conditions (Fig. S41). Moreover, the breakthrough experiments of C_2_H_2_/C_2_H_4_ (1/99) and C_2_H_6_/C_2_H_4_ (50/50) mixtures indicate that ZU-921 shows good C_2_H_6_-selective adsorptive performance, but poor C_2_H_2_/C_2_H_4_ separation performance (Fig. S43).

Given the importance of the recyclability and stability of porous materials for practical applications, the water and thermal stability of ZU-921 were investigated. Even under high humid conditions up to RH = 75%, the breakthrough performance of ZU-921 was invariable (Fig. S39). Meanwhile, the separation performance of ZU-921 was well maintained during the six cycling tests for C_3_H_4_/C_3_H_4_(PD)/C_3_H_8_/C_3_H_6_ and C_3_H_8_/C_3_H_6_ mixtures (Figs. 4d and S44–S46). No obvious structure degradation of ZU-921 was observed after it was exposed to different harsh conditions, such as humid air and high temperature (100 °C), as demonstrated by the invariant PXRD patterns and C_3_H_8_ adsorption isotherms (Fig. 4e). The impressive separation performance and the good stability of ZU-921 rendered it a promising adsorbent for one-step C_3_H_6_ purification from complex gas mixtures.

### 10-times scale-up breakthrough experiment

**Fig. 5:**
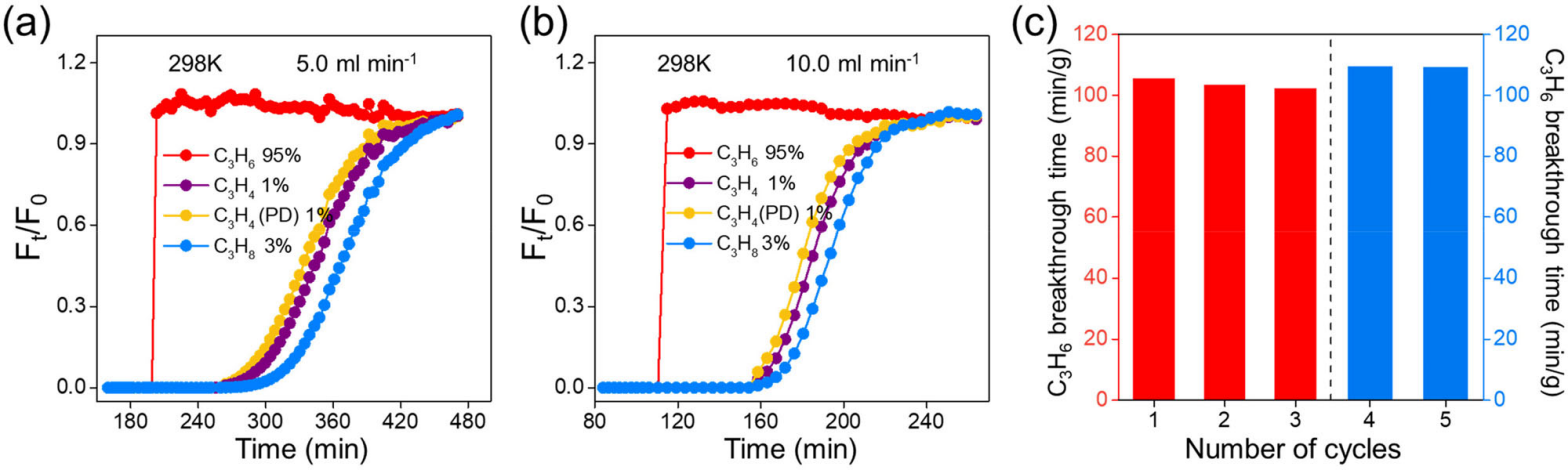
10-times scale-up breakthrough experiment. Dynamic breakthrough curves of ZU-921 for C_3_H_4_/C_3_H_4_(PD)/C_3_H_8_/C_3_H_6_ (1/1/3/95 *v/v/v/v*) mixture in F_t_/F_0_ at **a** 5.0 ml min^−1^, **b** 10.0 ml min^−1^, and **c** the recycling tests for C_3_H_4_/C_3_H_4_(PD)/C_3_H_8_/C_3_H_6_ (1/1/3/95 *v/v/v/v*, red; 25/25/25/25 *v/v/v/v*, light blue) after the regeneration under 393 K with the N_2_ flow rate of 20.0 mL min^−1^. (column: 1.0 cm × 50 cm, 20.5 g). Source data are provided as a Source Data file.

To further evaluate the application potential of ZU-921, we attempted the scale-up synthesis of ZU-921 and conducted the breakthrough experiments using the 10-times scale-up column (1.0 cm × 50 cm, 20.5 g) (Fig. S47). ZU-921 still exhibits impressive purification performance for different C_3_H_4_/C_3_H_4_(PD)/C_3_H_8_/C_3_H_6_ mixtures (1/1/3/95 *v/v/v/v* and 25/25/25/25 *v/v/v/v*) under different gas velocity (Figs. S48 andS49). The calculated C_3_H_6_ (99.99%) productivity is around 16.95 L/kg (5.0 mL/min) and 17.27 L/kg (10.0 mL/min) for the C_3_H_4_/C_3_H_4_(PD)/C_3_H_8_/C_3_H_6_ (1/1/3/95 *v/v/v/v*) mixture (Figs. 5a, b and S50–S52). The column could be regenerated with nitrogen purge at 393 K for 800 min, and during the five consecutive cycling breakthrough experiments, the separation performance of ZU-921 remained invariable (Figs. 5c, S53–S56). We also evaluated the theoretical energy consumption for the C_3_H_6_ production, and the value of the adsorption process is lower than the cascade catalytic hydrogenation and distillation process used in industry (Tables S10–S12).

### Modeling simulation studies

**Fig. 6:**
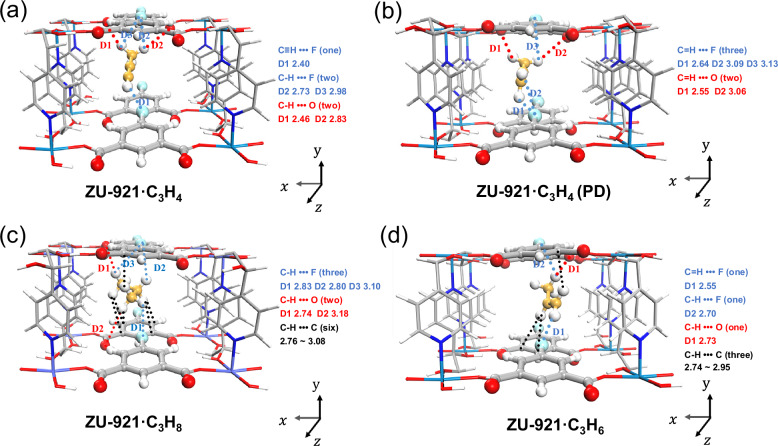
DFT-D calculated binding sites for C3 gases in ZU-921. **a** C_3_H_4_, **b** C_3_H_4_ (PD), **c** C_3_H_8_ and **d** C_3_H_6_ binding sites in ZU-921. The close contacts between framework atoms and the gas molecules are defined by the distances (in Å). (Framework: C, gray-80%; H, white; N, blue; O, red; Co, light blue; Gas: C, orange; H, white).

To reveal the molecular-level adsorption behavior of C3 gases within the channel of ZU-921, we performed detailed modeling studies using the first-principles dispersion-corrected density functional theory (DFT-D) method^45^. As shown in Figs. 6 and S57, all the C3 gases prefer to adsorb along the extending direction of the channel to get enough interactions with the parallel-arranged 5-fluoroisophthalic acid. In detail, C_3_H_4_ and C_3_H_4_(PD) are adsorbed in almost the same location. Each C_3_H_4_ molecule could interact with two negative fluorine atoms and two uncoordinated oxygen atoms via multiple cooperative hydrogen bonds (two C–H•••O (2.46 Å and 2.83 Å), two C–H•••F (2.73 Å and 2.98 Å) and C ≡ C–H•••F (2.40 Å)) (Fig. 6a). The C_3_H_4_(PD) molecule is bounded by two C = C–H•••O (2.55 Å and 3.06 Å) and three C = C–H••• F (2.64 Å, 3.09 Å and 3.13 Å) (Fig. 6b). While the C_3_H_8_ is inclined to interact with the paralleled three 5-fluoroisophthalic acid units via multiple van der Waals forces (six C–H•••C 2.76–3.08 Å) and multiple H-bonding interactions (C–H•••O 2.74 and 3.18 Å, C–H•••F 2.80, 2.83 and 3.10 Å) (Fig. 6c). In contrast, the interactions between ZU-921 and C_3_H_6_ are only provided by one C–H•••O (2.73 Å) and two C–H•••F (2.55 Å and 2.70 Å) and three C–H•••C (2.74–2.95 Å) interactions (Fig. 6d). The binding energies of C3 gases on ZU-921 follow the sequence of C_3_H_8_ (−56.43 kJ/mol) > C_3_H_4_(PD) (−55.20 kJ/mol)≈C_3_H_4_ (−54.64 kJ/mol) > C_3_H_6_(−52.96 kJ/mol), which is consistent with the calculated Q_st_ values of C3 gases based on their adsorption isotherms (Fig. S19). The results confirmed that ZU-921 could form a higher affinity with C_3_H_8_, C_3_H_4_, and C_3_H_4_ (PD) than C_3_H_6_. Simulation studies reveal that the polar fluorine, aromaticity sites, and uncoordinated oxygen are the keys to forming a synergistic binding environment for the simultaneous recognition of C_3_H_4_, C_3_H_4_ (PD), and C_3_H_8_ gases with different properties (Fig. S58).

## Discussion

In summary, we demonstrate the selective capture ability of ultramicroporous adsorbent ZU-921 for alkane, allene, and alkyne, achieving one-step C_3_H_6_ purification from C3 quaternary mixtures. The above remarkable progress in multiple impurities capture is attributed to the well-defined pore chemistry via ligand engineering strategy, the integrated binding sites of orderly lined aromatic units, as well as the quantified density of polar fluorine functional sites, enabling the exquisite control of priority affinity sequence of different molecules. Benefiting from the rational binding sequence, high-purity C_3_H_6_ (99.9% or 99.99%) could be easily obtained via a simple adsorption-desorption process from complex C3 mixtures. Our work not only presents an effective method to design advanced adsorbents for the simultaneous removal of multiple impurities but also demonstrates the great potential of adsorptive separation with tailor-made porous materials to simplify the complex separation process.

## Methods

### Chemicals

All reagents were analytical grade and used as received without further purification. Co(NO_3_)_2_·6H_2_O, Zn(NO_3_)_2_·6H_2_O, isophthalic acid (IPA), 5-fluoroisophthalic acid (IPA-F), 5-methylisophthalic acid (IPA-CH_3_), terephthalic acid (BDC), 2,5-difluoroterephthalic acid (BDC-2F) and tetrafluoroterephthalic acid (BDC-4F) methanol (MeOH), and dimethylformamide (DMF) were purchased from Aladdin Reagent Co. Ltd., Meso-α,β-Di(4-pyridyl) Glycol (DPG) was purchased from TCI Co. Ltd. 2,5-bis(trifluoromethyl) terephthalic acid (BDC-(CF_3_)_2_) and 1,4-diazabicyclo [2.2.2] octane (DABCO) were purchased from Aladdin. Ultrahigh purity grade He (99.999%), N_2_ (99.999%), C_3_H_4_ (99.9%), C_3_H_4_ (PD, 99.9%), C_3_H_6_ (99.99%), C_3_H_8_ (99.99%), and mixed gas (C_3_H_4_/C_3_H_6_ = 1/99, *v/v*, C_3_H_6_/C_3_H_8_ = 50/50, *v/v*, C_3_H_4_/C_3_H_4_(PD)/C_3_H_8_/C_3_H_6_ = 1/1/3/95, *v/v*/*v/v*, C_3_H_4_/C_3_H_4_(PD)/C_3_H_8_/C_3_H_6_ = 25/25/25/25, *v/v*/*v/v*) were purchased from Shanghai Wetry Standard gas Co., Ltd. (China) and used for all measurements.

### Material synthesis

PCP-IPA-X^33,49^ and the comparison materials of FDMOF-2^34^, Ni(ADC)(TED)_0.5_^48^, NKMOF-1-Ni^30^, NbOFFIVE-2-Cu-i^29^, SIFSIX-3-Ni^24^, and ZU-33^47^ were synthesized according to the previously reported procedure.

### ZU-921 (PCP-IPA-F)

81 mg DPG was dissolved in DMF/MeOH (1:1, 30 mL) at 60 °C, and 75 mg IPA-F and 109 mg Co(NO_3_)_2_·6H_2_O were dissolved in 5 mL MeOH. Then, the two solutions were mixed and heated at 80 °C for 24 h to yield as-synthesized ZU-921, with the yield reaching up to 82% (based on DPG ligand).

### Scale-up preparation of ZU-921

5.0 g DPG was dissolved in DMF/MeOH (1:1, 1.5 L) at 60 °C, and 4.63 g IPA-F and 6.7 g Co(NO_3_)_2_·6H_2_O were dissolved in 50 mL MeOH. Then, the two solutions were mixed and heated at 80 °C for 24 h to yield as-synthesized ZU-921, with the yield reaching up to 80% (based on DPG ligand).

### ZU-922 (PCP-IPA-CH_3_)

81 mg DPG was dissolved in DMF/MeOH (1:1, 30 mL) at 60 °C, and 75 mg IPA-CH_3_ and 109 mg Co(NO_3_)_2_·6H_2_O were dissolved in 5 mL MeOH. Then, the two solutions were mixed and heated at 80 °C for 24 h to yield as-synthesized ZU-922, with the yield reaching up to 80% (based on DPG ligand).

### ZU-923 (PCP-BDC-2F)

81 mg DPG was dissolved in DMF/MeOH (1:1, 30 mL) at 60 °C, and 80 mg BDC-2F and 109 mg Co(NO_3_)_2_·6H_2_O were dissolved in 5 mL MeOH. Then, the two solutions were mixed and heated at 80 °C for 24 h to yield as-synthesized ZU-923, with the yield reaching up to 77% (based on DPG ligand).

### ZU-924 (PCP-BDC-4F)

81 mg DPG was dissolved in DMF/MeOH (1:1, 30 mL) at 60 °C, and 95 mg BDC-4F and 109 mg Co(NO_3_)_2_·6H_2_O were dissolved in 5 mL MeOH. Then, the two solutions were mixed and heated at 80 °C for 24 h to yield as-synthesized ZU-924, with the yield reaching up to 75% (based on DPG ligand).

### FDMOF-2

A mixture of Zn(NO_3_)_2_·6H_2_O (0.4 mmol), the BDC-(CF_3_)_2_ (0.4 mmol) and DABCO (0.2 mmol), DMF (15 mL), and two drops of HNO_3_ were added into a 25 mL glass vial. After the mixture was stirred for 30 min, the vial was sealed and heated at 120 °C for 48 h to yield as-synthesized FDMOF-2 with the yield reaching up to 65% (based on BDC-(CF_3_)_2_ ligand).

### Sample characterization

Powder X-ray diffraction (PXRD) patterns were collected using a PANalytical Empyrean series 2 diffractometer with Cu-Ka radiation, at room temperature, with a step size of 0.0167°, a scan time of 15 s per step, and 2θ ranging from 5 to 50°. The thermogravimetric analysis (TGA) data were collected in a NETZSCH Thermogravimetric Analyzer (STA2500) from 50 to 700 °C with a heating rate of 10 °C/min. The morphology was investigated using a NOVA 200 Nanolab scanning electron microscope (SEM). The 195 K CO_2_ and 77 K N_2_ adsorption/desorption isotherms were obtained on a Micromeritics 3Flex and BSD-660 volumetric adsorption apparatus. The apparent Langmuir surface area was calculated using the adsorption branch with the relative pressure P/P_0_ in the range of 0.005–0.1. The total pore volume (V_tot_) was calculated based on the adsorbed amount of CO_2_ or N_2_ at the P/P_0_ of 0.99. The pore size distribution (PSD) was calculated using the H-K methodology with CO_2_ adsorption isotherm data and assuming a slit pore model.

### Gas adsorption measurements

The C_3_H_4_, C_3_H_4_(PD), C_3_H_6_, and C_3_H_8_ adsorption-desorption isotherms at different temperatures were measured volumetrically by Micromeritics 3Flex and BSD-660 adsorption apparatus for pressures up to 1.0 bar. Prior to the adsorption measurements, the samples were degassed using a high vacuum pump (<5 μm Hg) at 373 K for over 12 h.

### Kinetic adsorption measurement

The time-dependent adsorption profiles of C_3_H_4_, C_3_H_4_(PD), C_3_H_8_, and C_3_H_6_ were measured using an Intelligent Gravimetric Analyzer (IGA-100, HIDEN, U.K.). The diffusional time constants (D′, D/r^2^) were calculated by the short-time solution of the diffusion equation assuming a step change in the gas-phase concentration, clean beds initially, and micropore diffusion control:

$$\frac{{M}_{t}}{{M}_{e}}=\frac{6}{\sqrt{\pi }}.\sqrt{\frac{D}{{r}^{2}}}.\sqrt{t}$$

Where t (s) is the time, *M*_*t*_ (mmol/g) is the gas uptake at time t, *M*_e_ is the gas uptake at equilibrium (mmol/g), D (m^2^ s^−1^) is the diffusivity, and r (m) is the radius of the equivalent spherical particle. The slopes of *M*_*t*_/*M*_e_ versus$\sqrt{t}$ are derived from the fitting of the plots at 298 K and different adsorption pressures.

### Breakthrough experimental

The breakthrough experiments were carried out in a homemade apparatus under a standard procedure. First, the samples were degassed under vacuum at 393 K for 12 h and then were introduced to the adsorption column with different sizes (0.46 cm × 15 cm or 0.46 cm × 25 cm, or 1.0 cm × 50 cm). Second, the adsorption column was connected to the homemade apparatus, and the carrier gas (He ≥99.999%) purged the adsorption column for more than 1 h to ensure that the adsorption bed was clean and saturated with He. Third, we switched the carrier gas to the desired gas mixture without any inert gas dilution (C_3_H_4_/C_3_H_6_ = 1/99, *v/v*, C_3_H_6_/C_3_H_8_ = 50/50, *v/v*, C_3_H_4_/C_3_H_4_(PD)/C_3_H_8_/C_3_H_6_ = 1/1/3/95 *v/v*/*v/v*, C_3_H_4_/C_3_H_4_(PD)/C_3_H_8_/C_3_H_6_ = 25/25/25/25 *v/v*/*v/v*). Fourth, the desired gas flows through the column until the concentrations of all the components are consistent with the entrance of the gas mixture (temperature: 298 K, pressure: 1.0 bar). In this process, the eluted gas was passed to an analyzer port and analyzed using gas chromatography (GC490 Agilent) with a thermal conductivity detector (TCD), or gas chromatography (Shimadzu GC2010) with a flame ionization detector (FID). To obtain the breakthrough curves in F_t_/F_0_ (F_t_ and F_0_ are the flow rates of each gas at the outlet and inlet, respectively), the gas chromatography (Shimadzu GC2010) with a flame ionization detector (FID) is employed, and the loop capacity of the gas chromatography is 5 mL, and its injection time is 0.3 min. Within the 0–10 mL/min range of inlet gas flow rate (F_0_), the injection volume would not fill up the capacity of the loop, allowing the outlet gas flow rate (F_t_) of each gas to be determined from the peak area. After the breakthrough experiment, the adsorption column was regenerated at 393 K or 423 K with a 20 mL/min N_2_ flow rate for 10–12 h. Detailed experiment parameters like flow rates, temperatures, and column sizes are provided in every breakthrough curve.

### Isotherm fitting

The pure-component isotherms of C_3_H_4_, C_3_H_4_(PD), C_3_H_6_, and C_3_H_8_ were fitted using a two-site Langmuir-Freundlich model for the full range of pressure (0~1.0 bar).

1 $$q={q}_{{sat}1}\frac{{b}_{1}{p}^{v1}}{1+{b}_{1}{p}^{v1}}+{q}_{{sat}2}\frac{{b}_{2}{p}^{v2}}{1+{b}_{2}{p}^{v2}}$$

Here, p is the pressure of the bulk gas at equilibrium with the adsorbed phase (bar), q is the adsorbed amount per mass of adsorbent (mmol g^−1^), q_sat_ is the saturation capacity (mmol g^−1^), b is the affinity coefficient (bar^−1^), and v represents the deviation from an ideal homogeneous surface.

### Isosteric heat of adsorption

The isosteric heat of C_3_H_4_, C_3_H_4_(PD), C_3_H_6_, and C_3_H_8_ adsorption, Q_st_, defined as

2 $${Q}_{{st}}={{RT}}^{2}{\left(\frac{\partial {InP}}{\partial T}\right)}_{q}$$

were determined using the pure-component isotherm fits using the Clausius-Clapeyron equation. where Q_st_ (kJ/mol) is the isosteric heat of adsorption, T (K) is the temperature, P (bar) is the pressure, R is the gas constant, and q (mmol/g) is the adsorbed amount.

### IAST calculations

The selectivity of the preferential adsorption of component 1 over component 2 in a mixture containing 1 and 2 can be formally defined as:

3 $$S=\frac{{x}_{1}/{y}_{1}}{{x}_{2}/{y}_{2}\,}$$

In the above equation, x_1_ and y_1_ (x_2_ and y_2_) are the molar fractions of component 1 (component 2) in the adsorbed and bulk phases, respectively. We calculated the values of x_1_ and x_2_ using the Ideal Adsorbed Solution Theory (IAST) of Myers and Prausnitz^50^.

### Density functional theory calculations

First-principles density functional theory (DFT) calculations were performed using Materials Studio’s CASTEP code^45^. All calculations were conducted under the generalized gradient approximation (GGA) with Perdew−Burke−Ernzerhof (PBE). A semiempirical addition of dispersive forces to conventional DFT was included in the calculation to account for van der Waals interactions. Cutoff energy of 544 eV and a 2 × 2 × 3 k-point mesh was found to be enough for the total energy to be covered within 0.01 meV atom^−1^. The structures of the synthesized materials were first optimized from the refined structures. To obtain the binding energy, the pristine structure and an isolated gas molecule placed in a supercell (with the same cell dimensions as the pristine crystal structure) were optimized and relaxed as references. C_3_H_4_, C_3_H_4_(PD), C_3_H_6_, and C_3_H_8_ gas molecules were then introduced to different locations of the channel pore, followed by full structural relaxation. The static binding energy was calculated by the equation:

4 $${{{{\rm{E}}}}}_{B}={{{\rm{E}}}}({{{\rm{gas}}}})+{{{\rm{E}}}}({{{\rm{adsorbent}}}})-{{{\rm{E}}}}({{{\rm{adsorbent}}}}+{{{\rm{gas}}}})$$

### Structure simulation

The high-quality Powder X-ray diffraction (PXRD) data for Rietveld refinement of ZU-921 and ZU-922 frameworks were collected using a PANalytical Empyrean series 2 diffractometer with Cu-Ka radiation, at room temperature, with a step size of 0.01313°, a scan time of 198.65 s per step, and 2θ ranging from 5 to 90°. As indicated by the similar PXRD patterns and assembled modules, the structure of ZU-921 and ZU-922 is speculated to be isostructural to PCP-IPA except for the different substituted groups (–F, –CH_3_, and –H) (Fig. S2). We built the initial raw structure of ZU-921 and ZU-922 based on the framework of PCP-IPA using Material Studio. The Rietveld refinement, a software package for crystal determination from the XRD pattern, was performed to optimize the lattice parameters iteratively until the *w*Rp value converges. The Reflex Powder Solve was employed to further optimize the atomic positions, bond length, bond angle, etc. The pseudo-Voigt profile function was used for whole profile fitting, and the Berrar-Baldinozzi function was used for asymmetry correction during the refinement processes. Line broadening from crystallite size and lattice strain was considered.

## Supplementary information

Supplementary Information

Transparent Peer Review file

Author Checklist

## Source data

Source Data files

## Data Availability

Crystallographic data for the structures reported in this article have been deposited at the Cambridge Crystallographic Data Centre, under deposition numbers CCDC 2294207 (for ZU-921) and 2294206 (for ZU-922). Copies of the data can be obtained free of charge via https://www.ccdc.cam.ac.uk/structures/. The authors declare that the main data supporting the findings of this study are available within the article and its Supplementary Information files. Source data that support the findings of this study are provided as a source data file. Source data are provided with this paper.
