# Supplementary material for: One-step propylene purification from a quaternary mixture by a single physisorbent
Source: Nat Commun. 2025 Dec 13;16:11316. doi: 10.1038/s41467-025-66438-9 (PMC12722248; doi:10.1038/s41467-025-66438-9)
Supplement: Supplementary file 1 — Supplementary Information [file 41467_2025_66438_MOESM1_ESM.pdf]

Supporting information

One-step Propylene Purification from a Quaternary Mixture by a Single Physisorbent

Peixin Zhang, [1,2] Zhensong Qiu, [1] Yechen Liu, [1] Sen Chen, [1,2] Lifeng Yang*, [1,3] Xian Suo, [2] Xili Cui, [1,2] Huabin Xing* [1,2]

1. Zhejiang Key Laboratory of Intelligent Manufacturing for Functional Chemicals, College of Chemical and Biological Engineering, Zhejiang University, Hangzhou 310012, China
2. Engineering Research Center of Functional Materials Intelligent Manufacturing of Zhejiang Province, Institute for Intelligent Bio/Chem Manufacturing, ZJU-Hangzhou Global Scientific and Technological Innovation Center, Hangzhou 311200, China
3. State Key Laboratory of Silicon Materials, School of Materials Science and Engineering, Zhejiang University, Hangzhou 310027, China

*Corresponding author: Prof. H. Xing, E-mail: xinghb@zju.edu.cn, and L. Yang, lifeng_yang@zju.edu.cn

Supplementary Figures

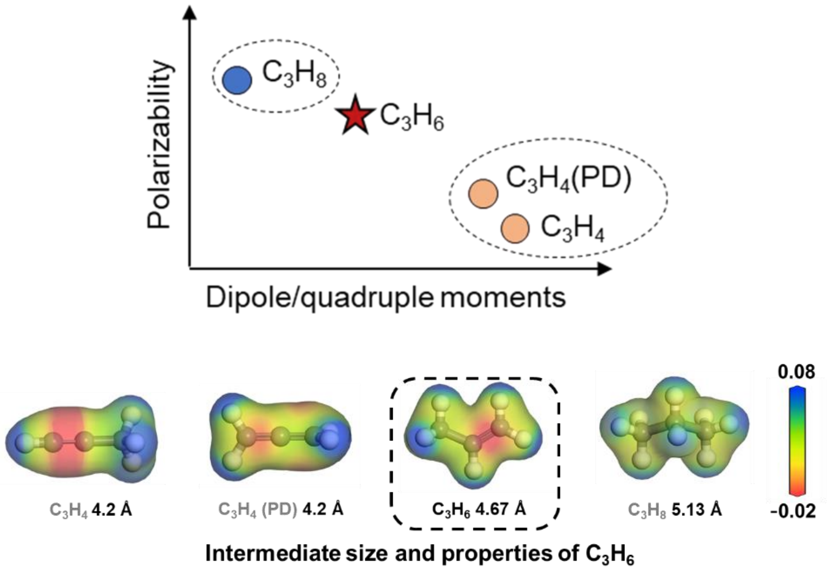

Supplementary Figure 1. Gas properties. The properties difference of C_3H_4, C_3H_4 (PD), C_3H_6 and C_3H_8

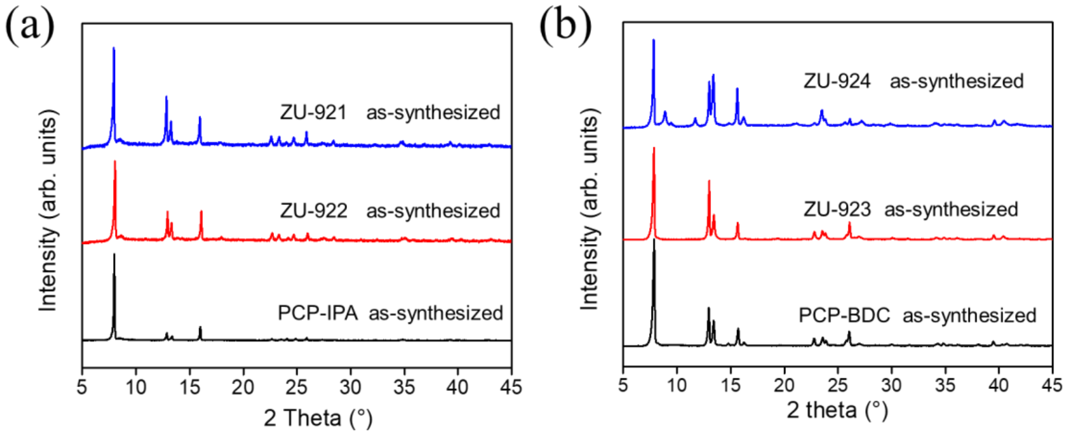

Supplementary Figure 2. PXRD patterns. The similar PXRD patterns of as-synthesized (a) PCP-IPA, ZU-921, and ZU-922 (b) PCP-BDC, ZU-923, and ZU-924. Similar patterns indicate their same structure.

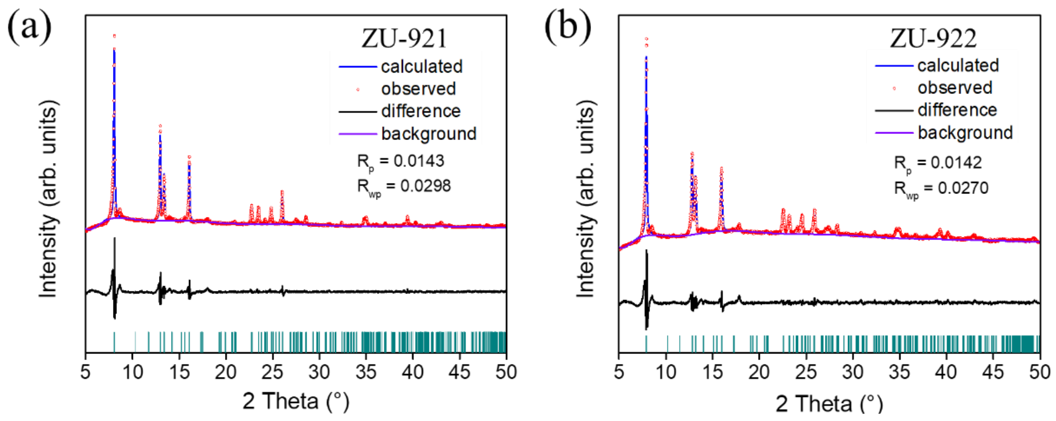

Supplementary Figure 3. Rietveld refinements of structure. Rietveld refinements of the powder diffraction data for the activated (a) ZU-921 and (b) ZU-922 (collected at room temperature).

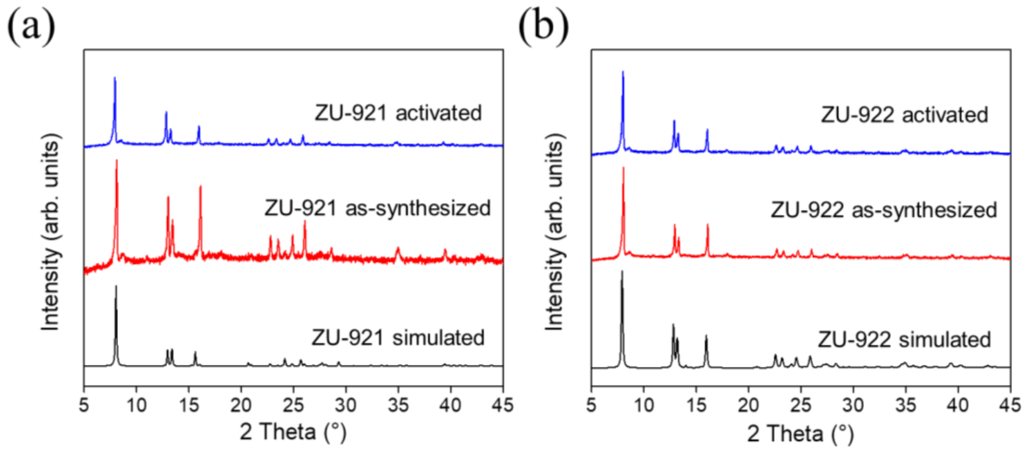

Supplementary Figure 4. PXRD patterns. The PXRD patterns of (a) ZU-921 and (b) ZU-922.

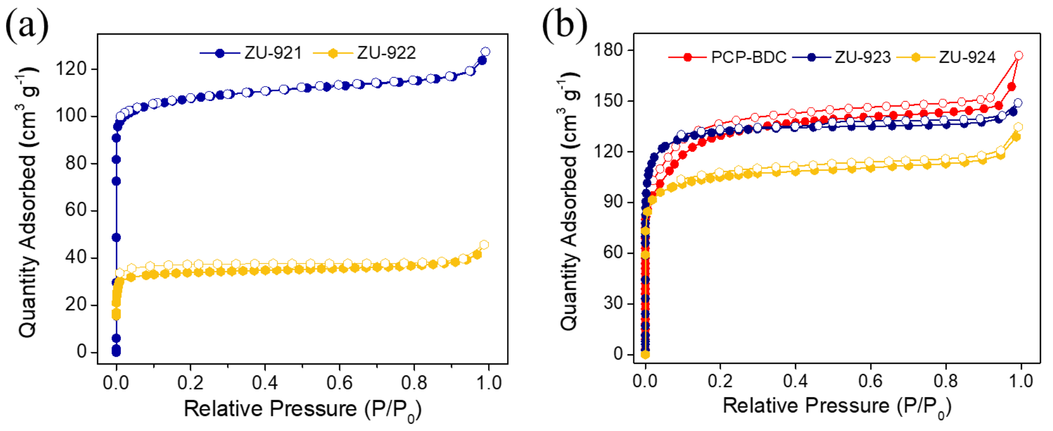

Supplementary Figure 5. Pore structure property. The 77 K N_2 adsorption isotherms of (a) ZU-921 and ZU-922 (b) PCP-BDC, ZU-923, and ZU-924. Source data are provided as a Source Data file.

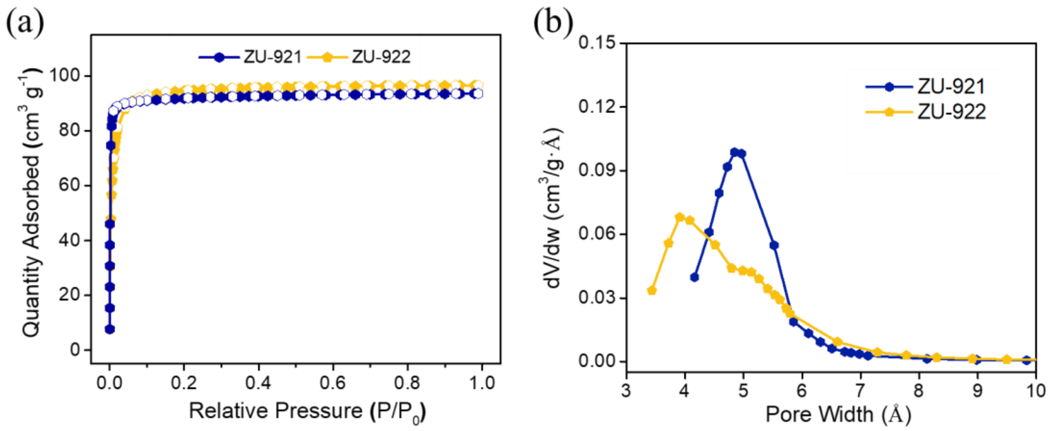

Supplementary Figure 6. Pore structure property. (a) The 195 K CO_2 adsorption isotherms and (b) H-K pore size distribution of ZU-921 and ZU-922. Source data are provided as a Source Data file.

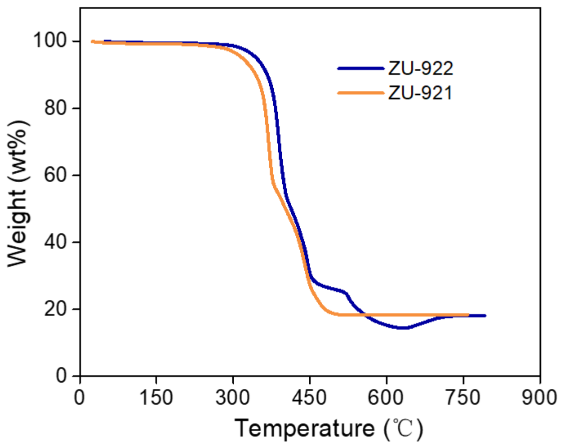

Supplementary Figure 7. Thermal stability. The TGA curves of ZU-921 and ZU-922. Source data are provided as a Source Data file.

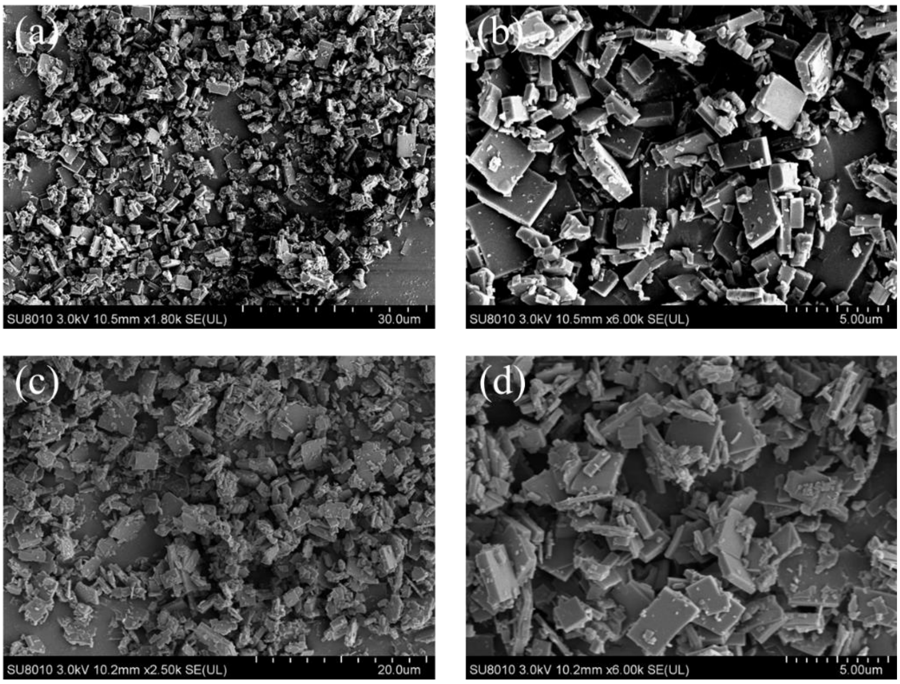

Supplementary Figure 8. SEM images. SEM images of ZU-921 (a) (b) and ZU-922 (c) (d).

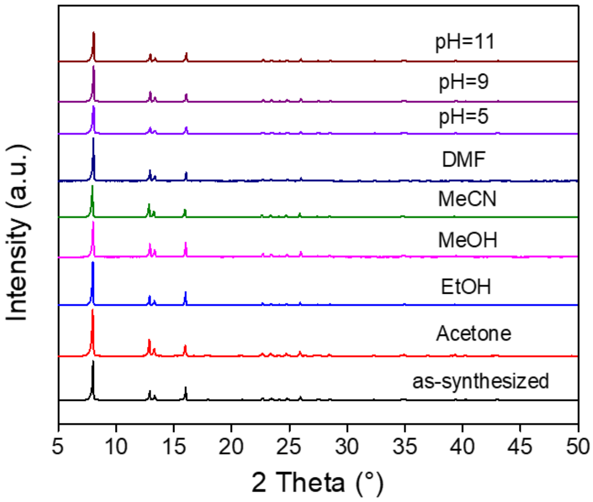

Supplementary Figure 9. **Chemical Stability.** Powder X-ray diffraction patterns of ZU-921 after different treatments.

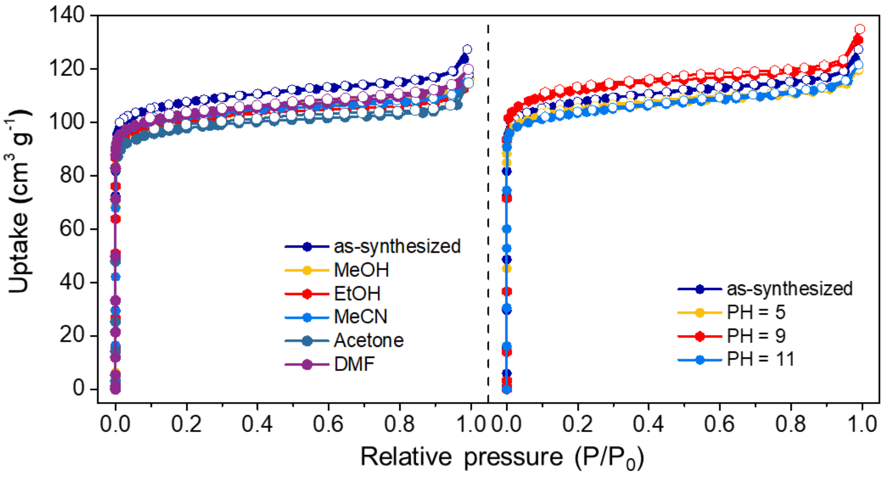

Supplementary Figure 10. **Chemical Stability.** The 77 N_2 adsorption isotherms of ZU-921 after different solvent and pH conditions treatment. Source data are provided as a Source Data file.

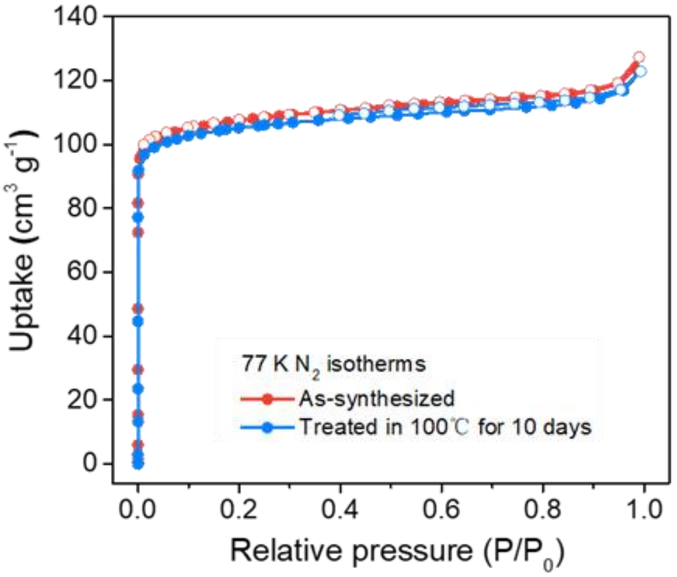

Supplementary Figure 11. **Thermal stability.** The 77 N_2 adsorption isotherms of ZU-921 treated at 100 ^{0}C for 10 days. Source data are provided as a Source Data file.

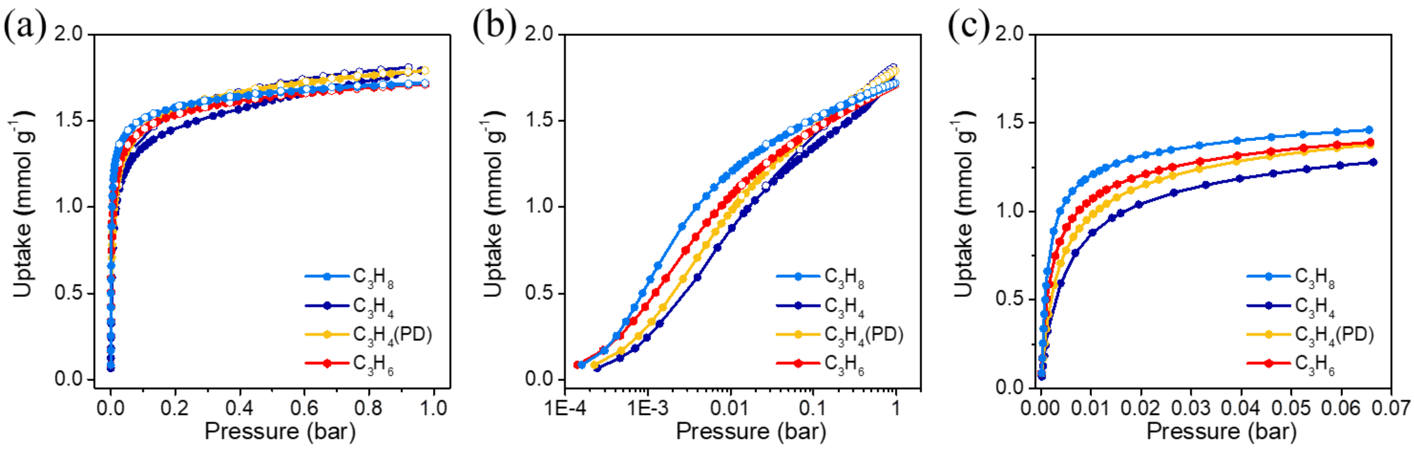

Supplementary Figure 12. Adsorption isotherms of ZU-922. The single-component adsorption isotherms of C3 gases of ZU-922 at 298 K with (a) a linear scale under the pressure range of 0-1.0 bar, (b) a logarithm scale under the pressure range of 0-1.0 bar, (c) a linear scale under the pressure range of 0-0.06 bar. Source data are provided as a Source Data file.

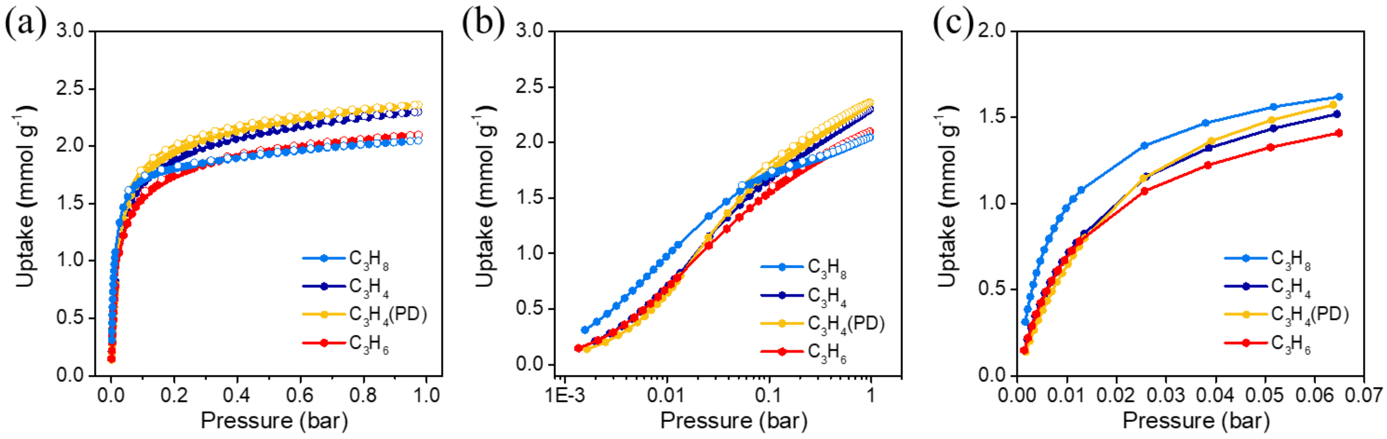

Supplementary Figure 13. Adsorption isotherms of PCP-BDC. The single-component adsorption isotherms of C3 gases of PCP-BDC at 298 K with (a) a linear scale under the pressure range of 0-1.0 bar, (b) a logarithm scale under the pressure range of 0-1.0 bar, (c) a linear scale under the pressure range of 0-0.06 bar. Source data are provided as a Source Data file.

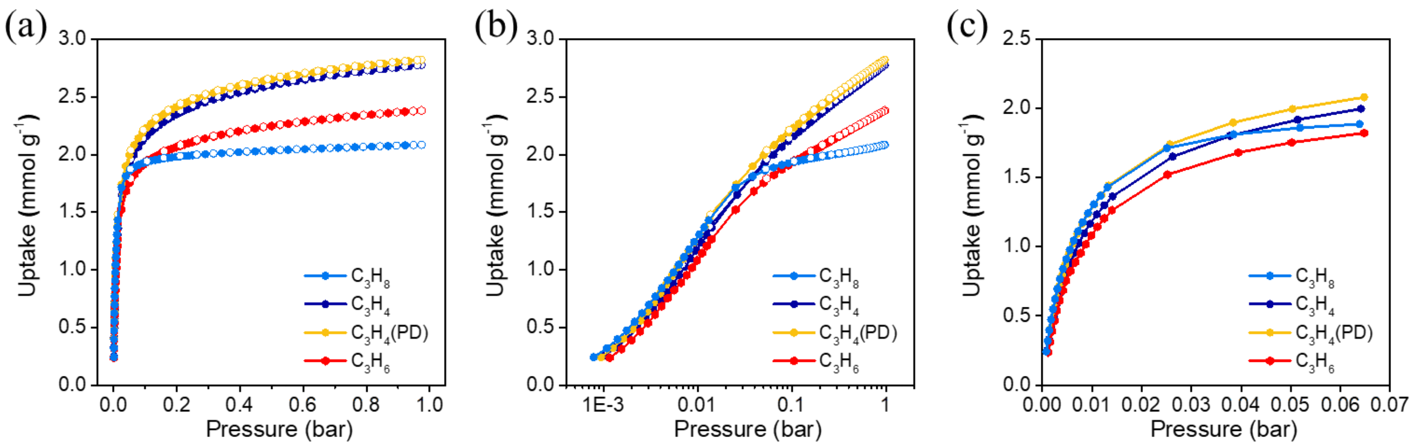

Supplementary Figure 14. Adsorption isotherms of ZU-923. The single-component adsorption isotherms of C3 gases of ZU-923 at 298 K with (a) a linear scale under the pressure range of 0-1.0 bar, (b) a logarithm scale under the pressure range of 0-1.0 bar, (c) a linear scale under the pressure range of 0-0.06 bar. Source data are provided as a Source Data file.

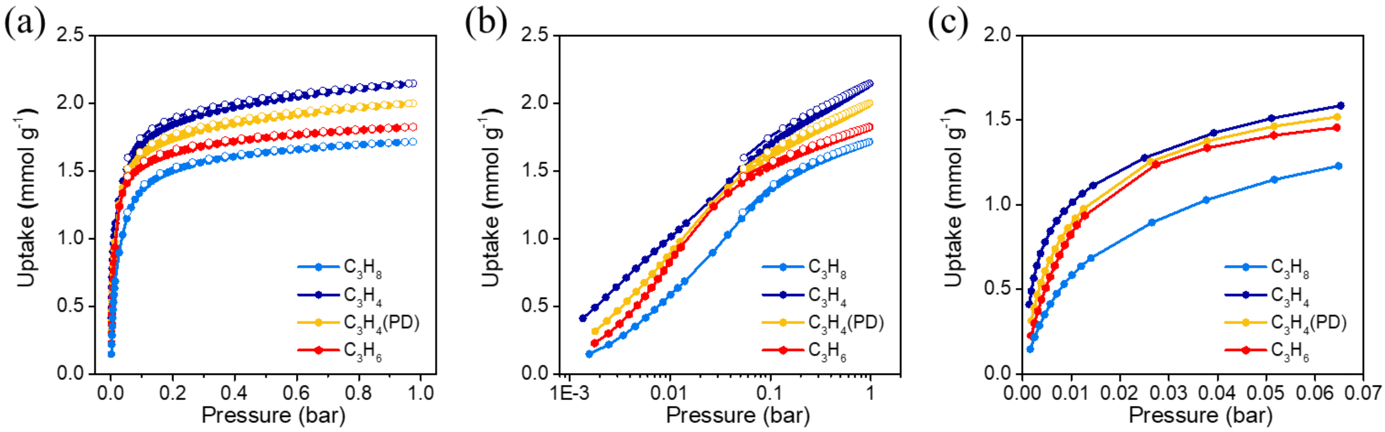

Supplementary Figure 15. Adsorption isotherms of ZU-924. The single-component adsorption isotherms of C3 gases of ZU-924 at 298 K with (a) a linear scale under the pressure range of 0-1.0 bar, (b) a logarithm scale under the pressure range of 0-1.0 bar, (c) a linear scale under the pressure range of 0-0.06 bar. Source data are provided as a Source Data file.

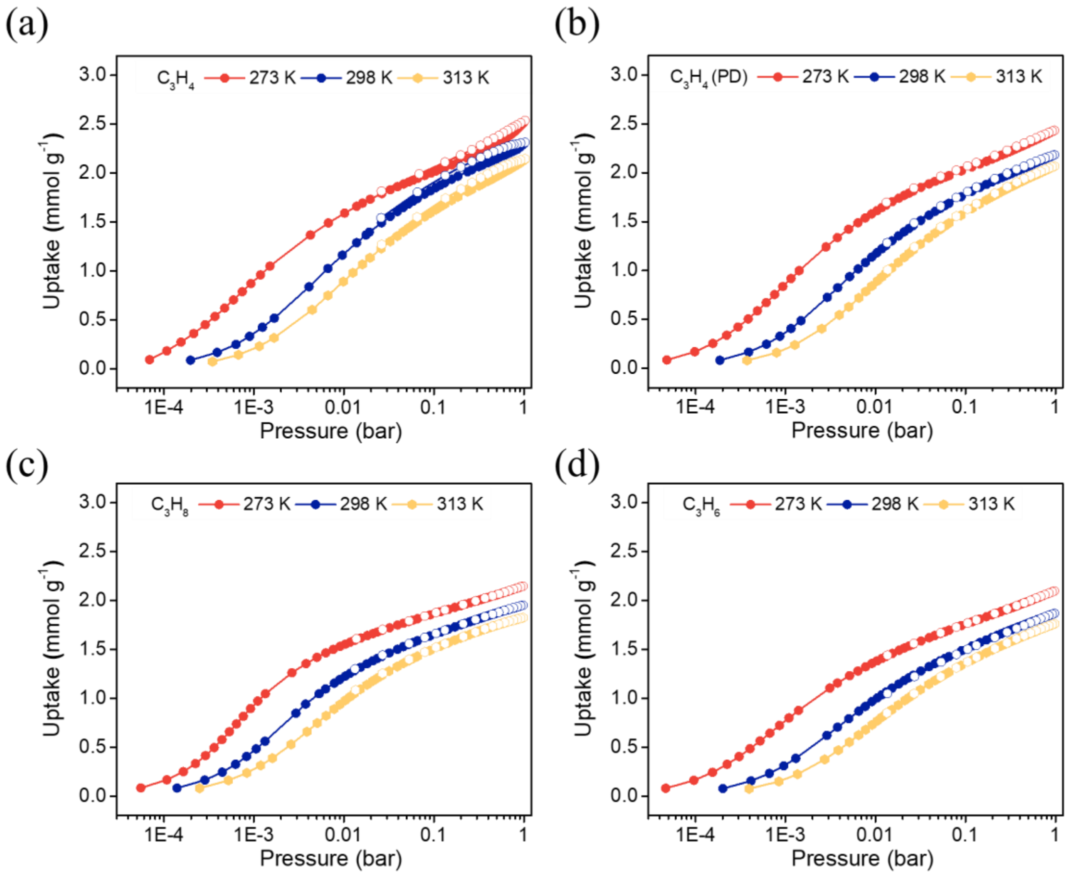

Supplementary Figure 16. Adsorption isotherms of ZU-921. (a) C_3H_4, (b) C_3H_4 (PD), (c) C_3H_8, (d) C_3H_6 adsorption-desorption isotherms of ZU-921 under three different temperatures. Source data are provided as a Source Data file.

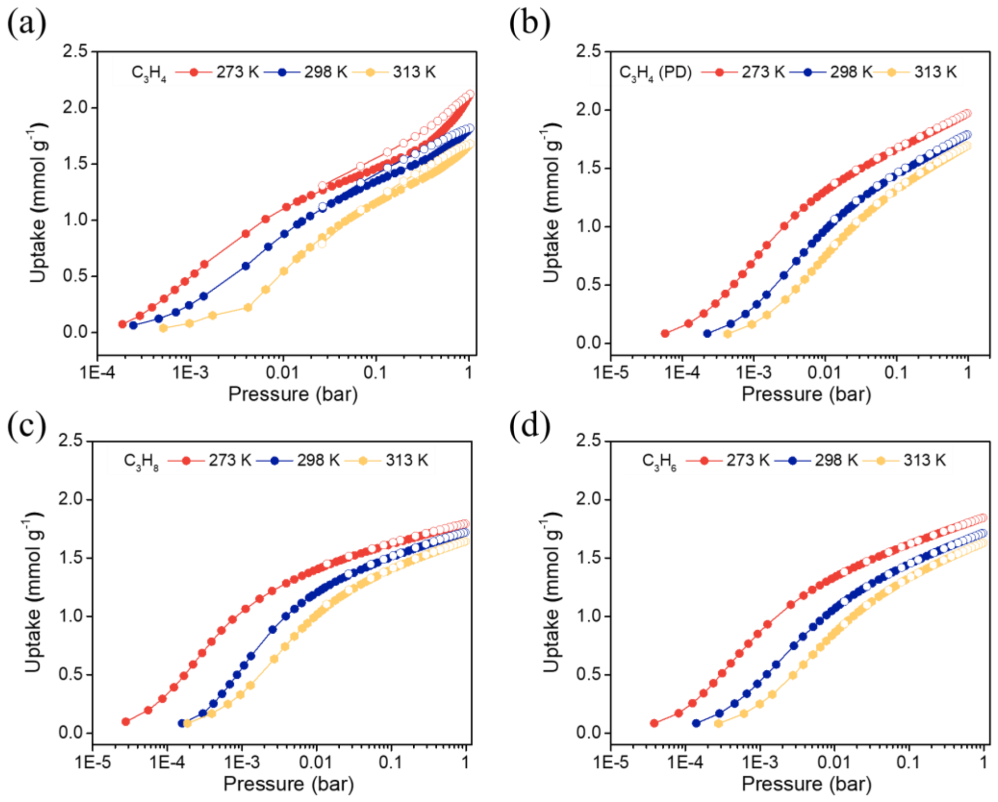

Supplementary Figure 17. Adsorption isotherms of ZU-922. (a) C_3H_4, (b) C_3H_4 (PD), (c) C_3H_8, (d) C_3H_6 adsorption-desorption isotherms of ZU-922 under three different temperatures. Source data are provided as a Source Data file.

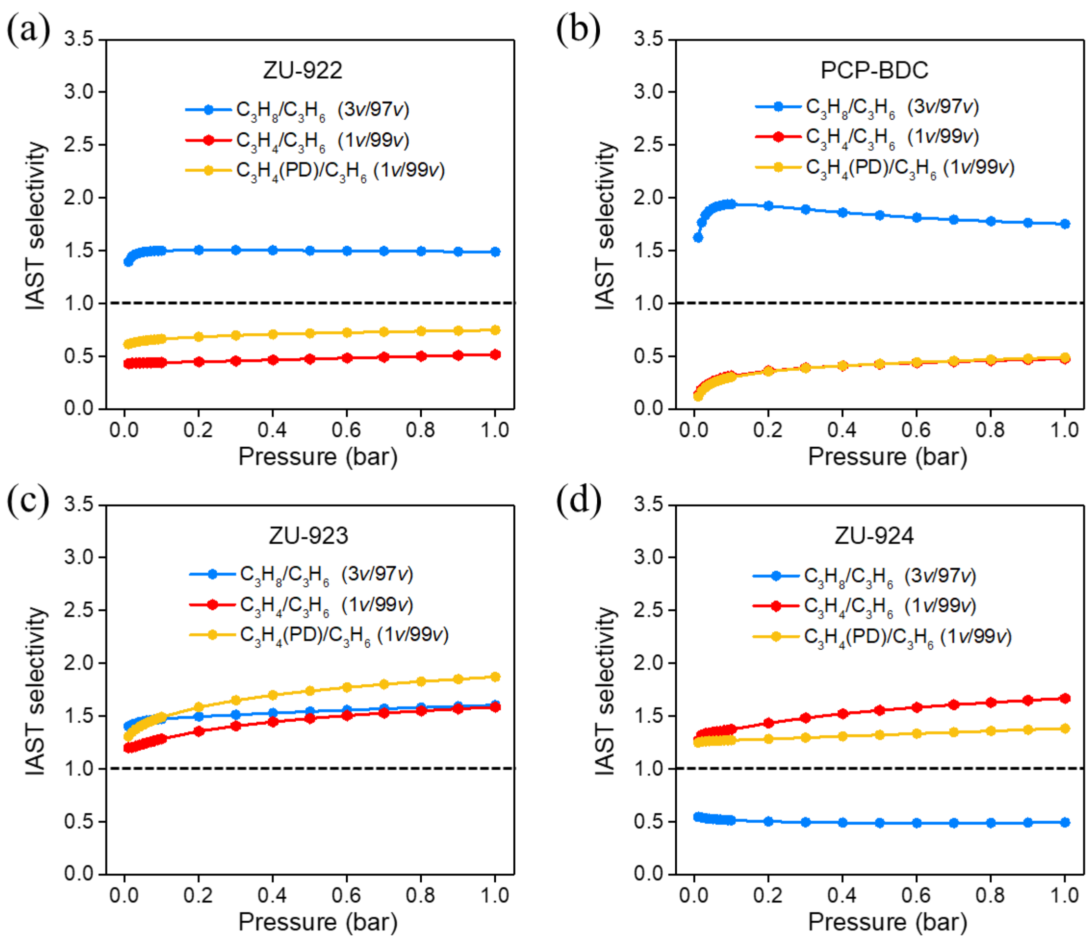

Supplementary Figure 18. IAST selectivity. The IAST selectivity of C3 gases binary mixtures on ZU-922, PCP-BDC, ZU-923 and ZU-924.

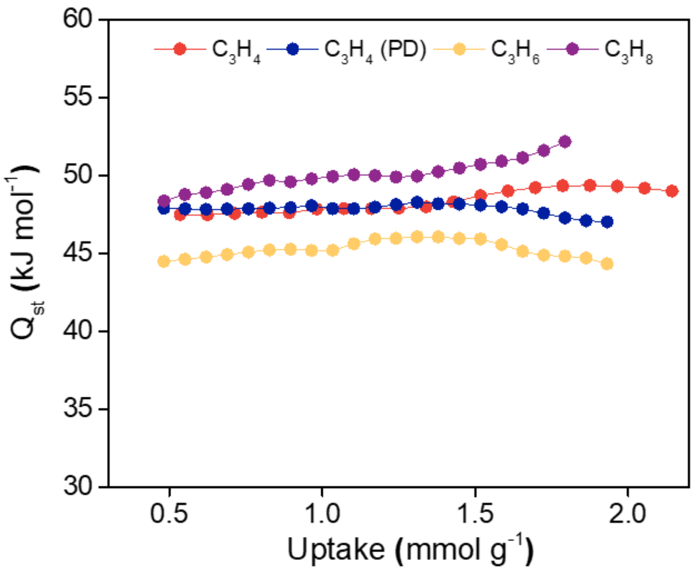

Supplementary Figure 19. Adsorption heat curves. The C_3H_8, C_3H_4, C_3H_4 (PD) and C_3H_6 isosteric heat of adsorption on ZU-921.

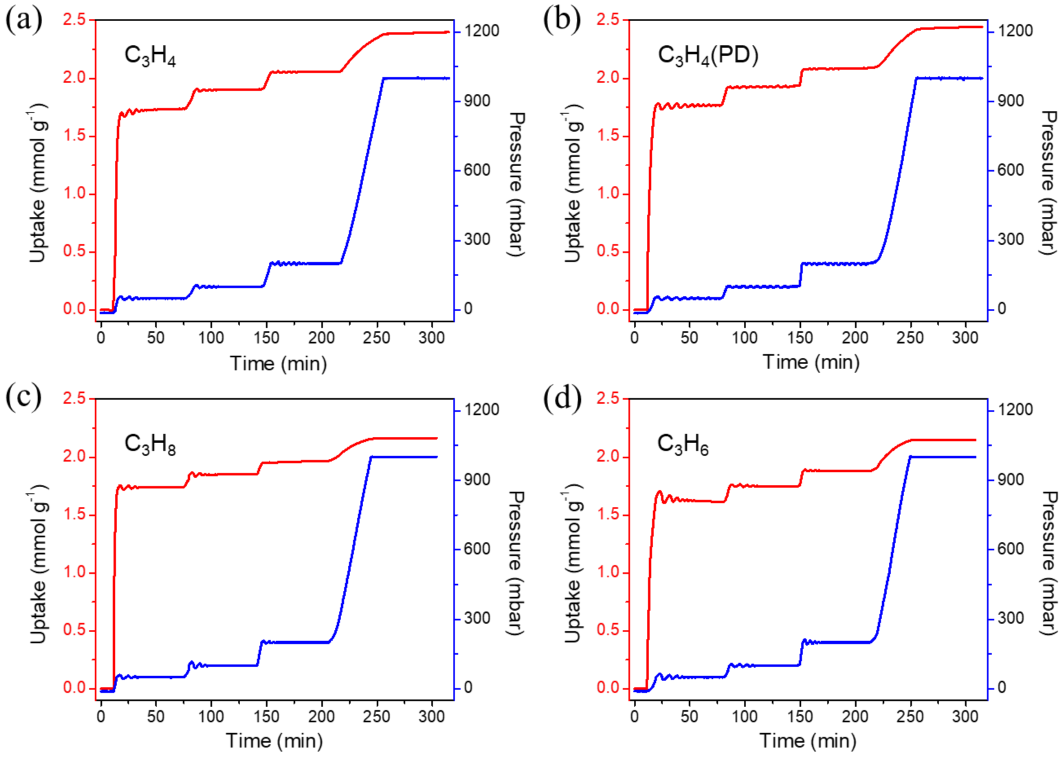

Supplementary Figure 20. Adsorption kinetics. Time-dependent gas uptake profiles of (a) C_3H_4 (b) C_3H_4 (PD) (c) C_3H_8 (d) C_3H_6 at 298 K on ZU-921. Source data are provided as a Source Data file.

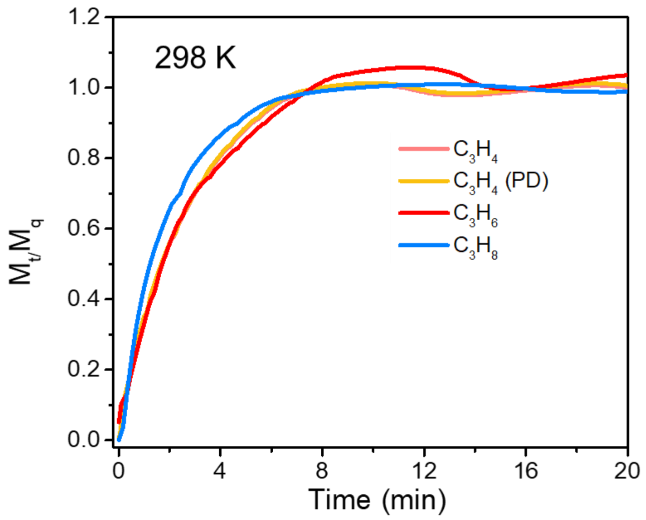

Supplementary Figure 21. Adsorption kinetics. Time-dependent gas uptake profiles of C3 gases on ZU-921 under 298 K and the pressure range of 0-50 mbar.

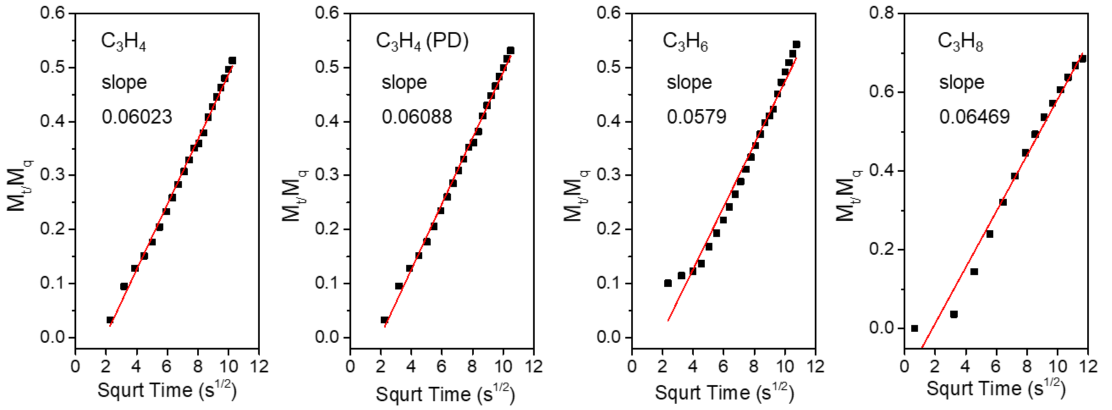

Supplementary Figure 22. Adsorption kinetics. Fitting of diffusion time constants based on time-dependent gas uptake profiles of ZU-921.

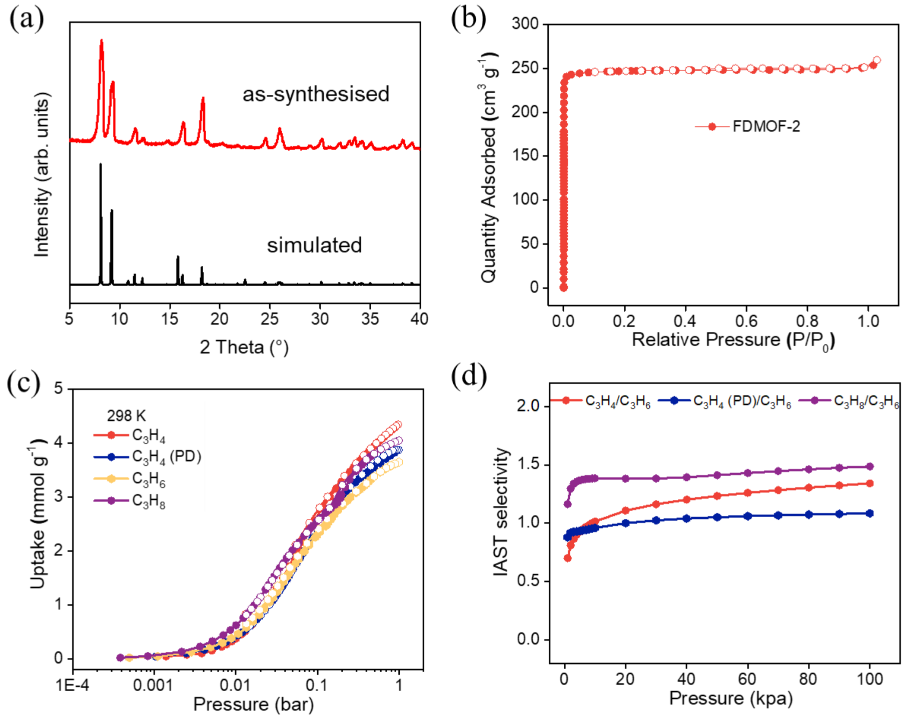

Supplementary Figure 23. Characterization of material structure and adsorption separation performance. (a) The PXRD patterns of FDMOF-2 (b) 77 K N_2 sorption isotherms (c) The single-component adsorption isotherms of C3 gases and (d) the IAST selectivity for C_3H_4/C_3H_6 (1/99, v/v), C_3H_4 (PD)/C_3H_6 (1/99, v/v) and C_3H_8/C_3H_6 (3/97, v/v) mixtures at 298 K on FDMOF-2. Source data are provided as a Source Data file.

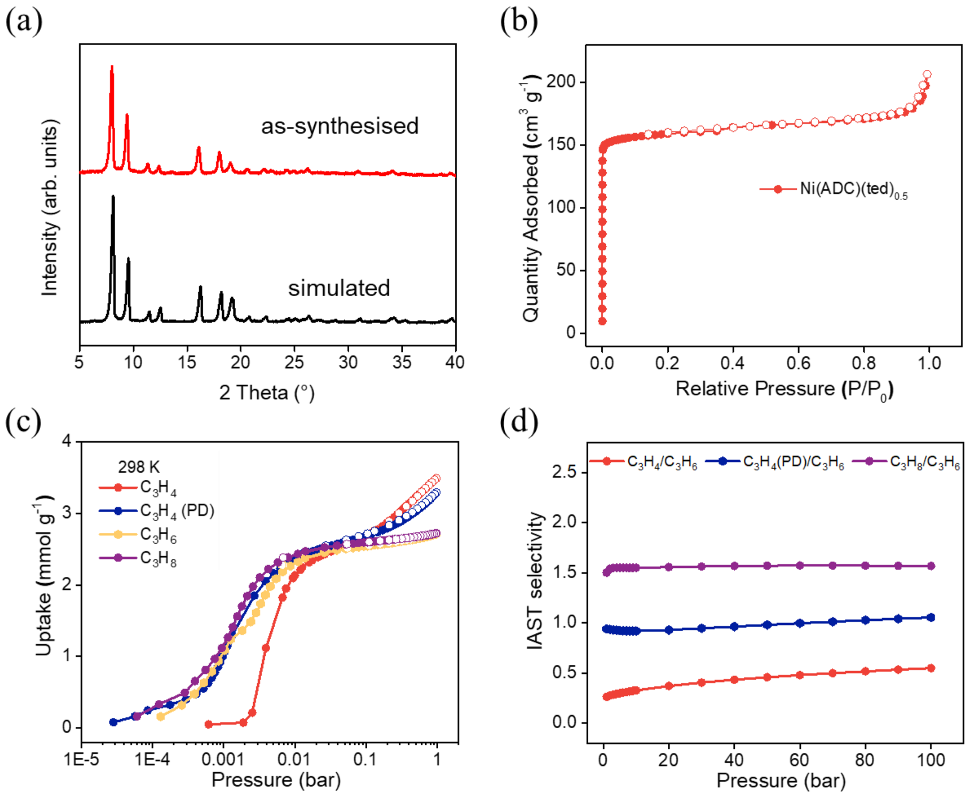

Supplementary Figure 24. Characterization of material structure and adsorption separation performance. (a) The PXRD patterns (b) 77 K N_2 sorption isotherms (c) The single-component adsorption isotherms of C3 gases and (d) the IAST selectivity for C_3H_4/C_3H_6 (1/99, v/v), C_3H_4 (PD)/C_3H_6 (1/99, v/v) and C_3H_8/C_3H_6 (3/97, v/v) mixtures at 298 K on Ni(ADC)(ted)$_{0.5}$. Source data are provided as a Source Data file.

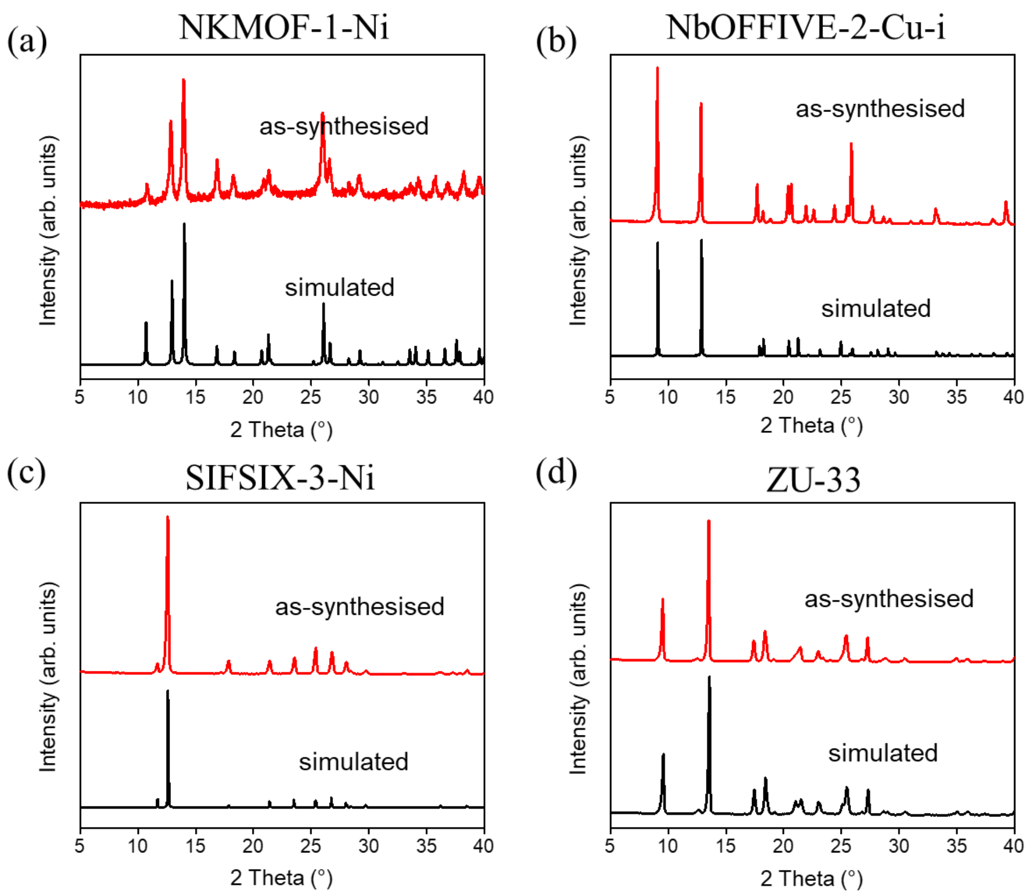

Supplementary Figure 25. Characterization of material structure. The PXRD patterns (a) NKMOF-1-Ni (b) NbOFFIVE-2-Cu-i (c) SIFSIX-3-Ni and (d) ZU-33

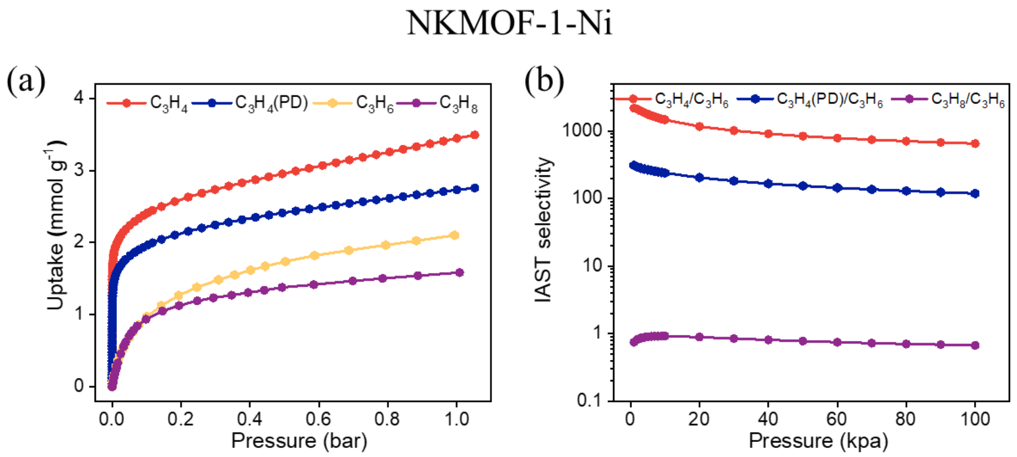

Supplementary Figure 26. Adsorption separation performance. (a) The single-component adsorption isotherms of C3 gases and (b) the IAST selectivity for the C_3H_4/C_3H_6 (1/99, v/v), C_3H_4 (PD)/C_3H_6 (1/99, v/v) and C_3H_8/C_3H_6 (3/97, v/v) mixtures at 298 K on NKMOF-1-Ni. Source data are provided as a Source Data file.

NbOFFIVE-2-Cu-i

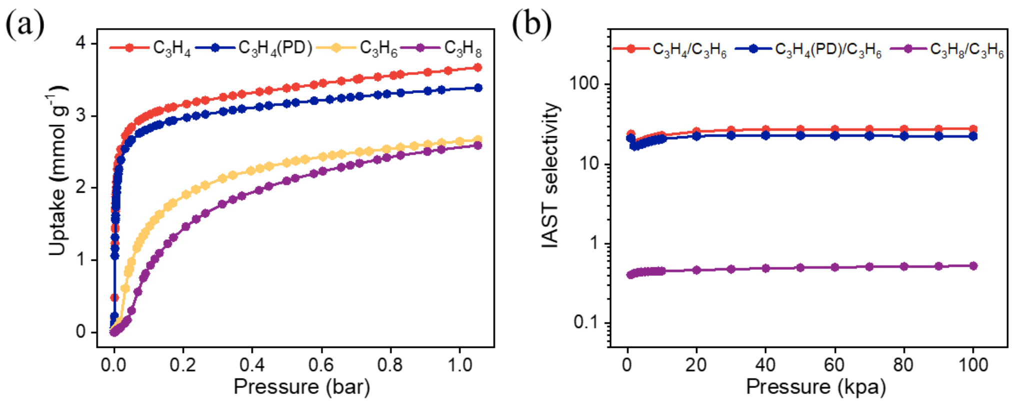

Supplementary Figure 27. Adsorption separation performance. (a) The single-component adsorption isotherms of C3 gases and (b) the IAST selectivity for the C_3H_4/C_3H_6 (1/99, v/v), C_3H_4 (PD)/C_3H_6 (1/99, v/v) and C_3H_8/C_3H_6 (3/97, v/v) mixtures at 298 K on NbOFFIVE-2-Cu-i. Source data are provided as a Source Data file.

SIFSIX-3-Ni

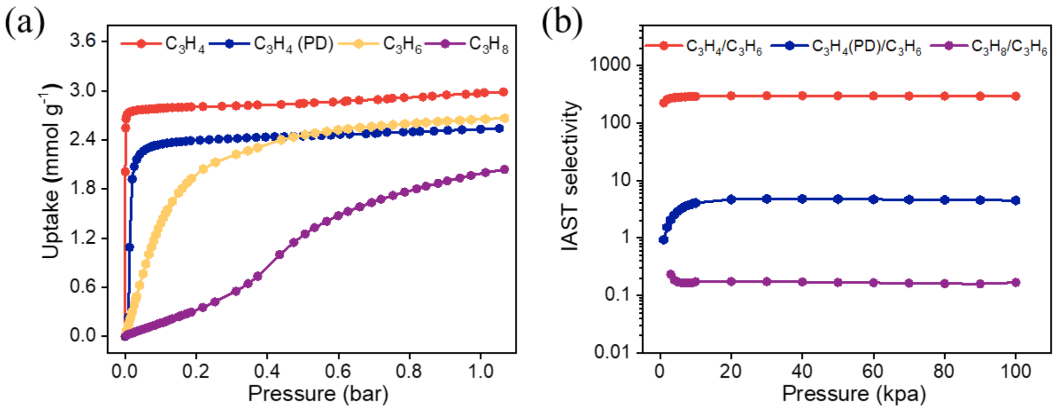

Supplementary Figure 28. Adsorption separation performance. (a) The single-component adsorption isotherms of C3 gases and (b) the IAST selectivity for C_3H_4/C_3H_6 (1/99, v/v), C_3H_4 (PD)/C_3H_6 (1/99, v/v) and C_3H_8/C_3H_6 (3/97, v/v) mixtures at 298 K on SIFSIX-3-Ni. Source data are provided as a Source Data file.

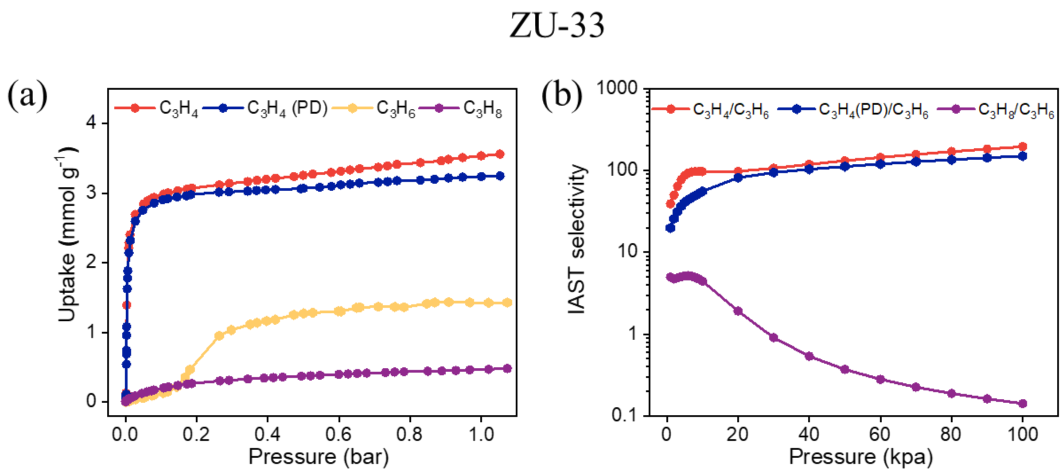

Supplementary Figure 29. Adsorption separation performance. (a) The single-component adsorption isotherms of C3 gases and (b) the IAST selectivity for C_3H_4/C_3H_6 (1/99, v/v), C_3H_4 (PD)/C_3H_6 (1/99, v/v) and C_3H_8/C_3H_6 (3/97, v/v) mixtures at 298 K on ZU-33. Source data are provided as a Source Data file.

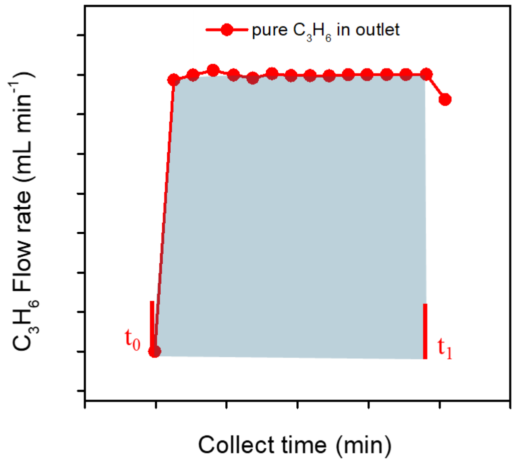

The productivity (q) of C_3H_6 is calculated as:

$$Q = \frac{\int_{t0}^{t1} f(t)\,dt}{m} = \frac{v_{c3h6} \times (t_1 - t_0)}{m}$$

v_{c3h6} refers to the outlet flow rate of C_3H_6, m refers to the mass of the adsorbent.

Supplementary Figure 30. Diagram for productivity calculation. The calculation diagram of C_3H_6 productivity based on its corrected outlet flow rate.

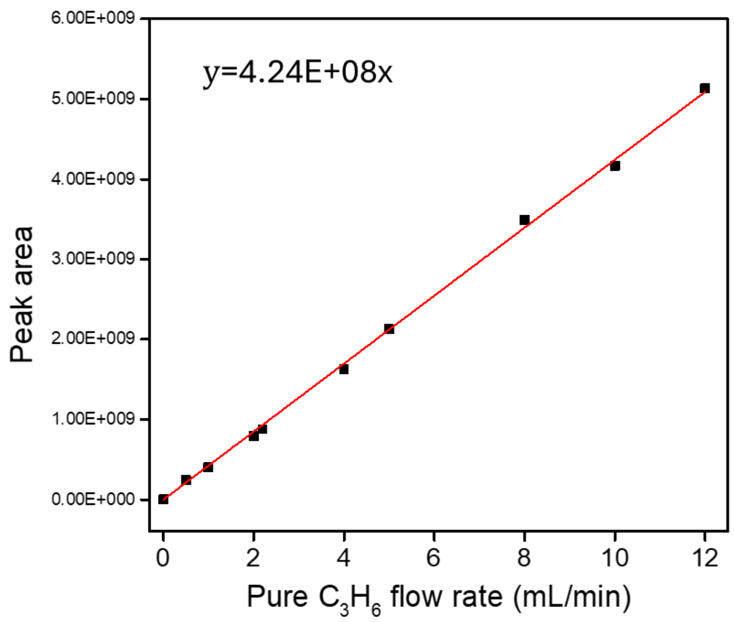

Supplementary Figure 31. Calibration curve. Calibration curve of pure C_3H_6 flow rate versus its peak area on the gas chromatogram.

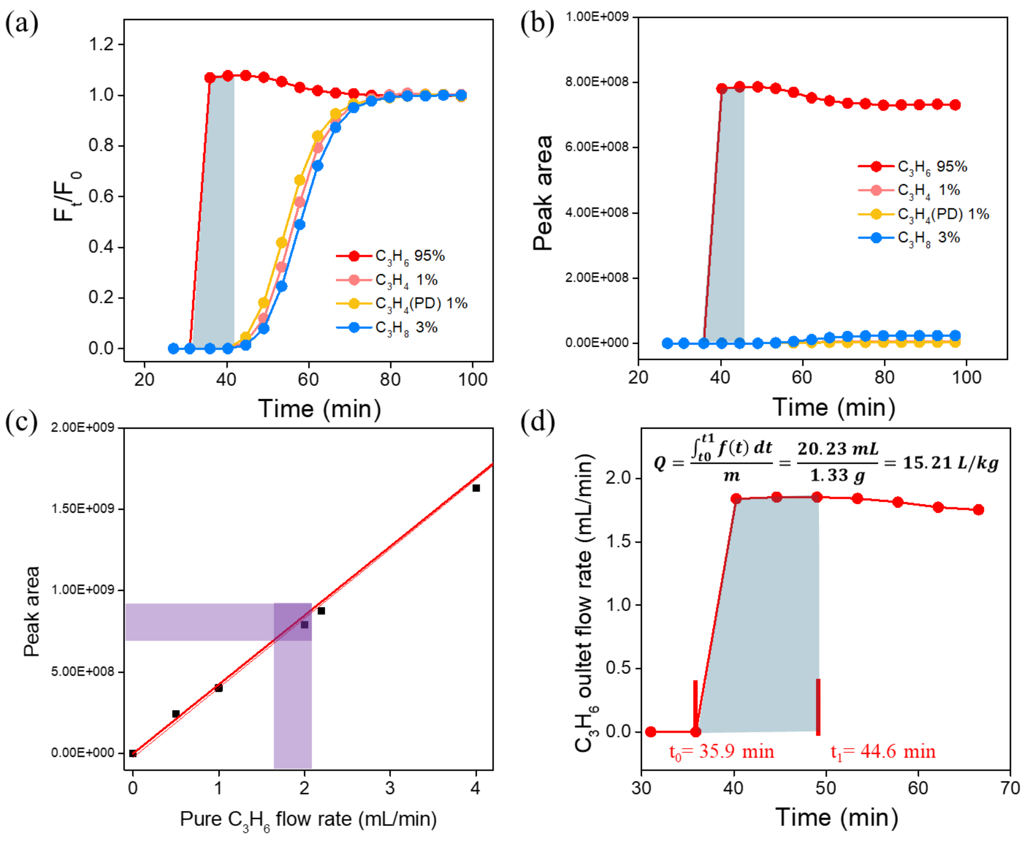

Supplementary Figure 32. Dynamic separation performance. The breakthrough curves of ZU-921 (0.46 cm x 15 cm, 1.33 g) and its C_3H_6 productivity. (a) Breakthrough curve of C_3H_4/C_3H_4(PD)/C_3H_8/C_3H_6 (1/1/3/95 *v/v/v/v*) mixture at the constant inlet flow rate of 2.2 mL/min under 298 K (F_t and F_0 are the flow rates of each gas at the outlet and inlet, respectively), (b) Real-time peak area of each gas during the breakthrough measured by Shimadzu GC2010, (c) Calibration curve of pure C_3H_6 flow rate versus C_3H_6 peak area on the gas chromatogram, (d) Based on calibration curve between flow rate and C_3H_6 peak area, the real-time outlet C_3H_6 flow rate could be calculated and the corresponding C_3H_6 productivity could be calculated, around 15.21 L/kg. Source data are provided as a Source Data file.

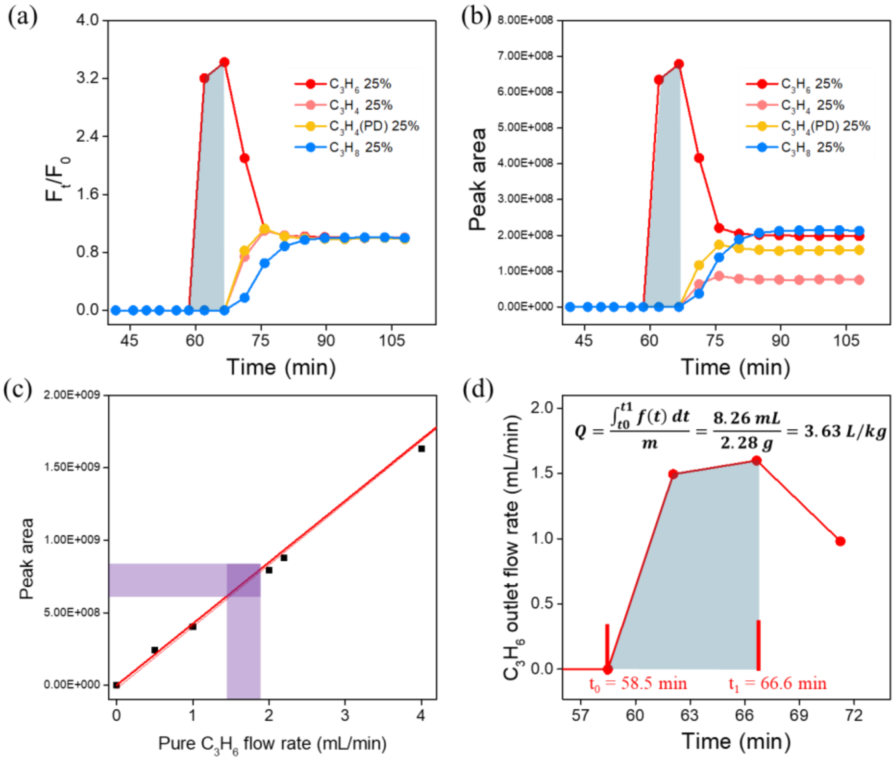

Supplementary Figure 33. Dynamic separation performance. The breakthrough curves of ZU-921 (0.46 cm × 25 cm, 2.28 g) and its C_3H_6 productivity. (a) Breakthrough curve of C_3H_4/C_3H_4(PD)/C_3H_8/C_3H_6 (25/25/25/25 *v/v/v/v*) mixture at the constant inlet flow rate of 2.2 mL/min under 298 K (F_t and F_0 are the flow rates of each gas at the outlet and inlet, respectively), (b) Real-time peak area of each gas during the breakthrough measured by Shimadzu GC2010, (c) Calibration curve of pure C_3H_6 flow rate versus C_3H_6 peak area on the gas chromatogram, (d) Based on calibration curve between flow rate and C_3H_6 peak area, the real-time outlet C_3H_6 flow rate could be calculated, and the corresponding C_3H_6 productivity could be calculated, around 3.63 L/kg. Source data are provided as a Source Data file.

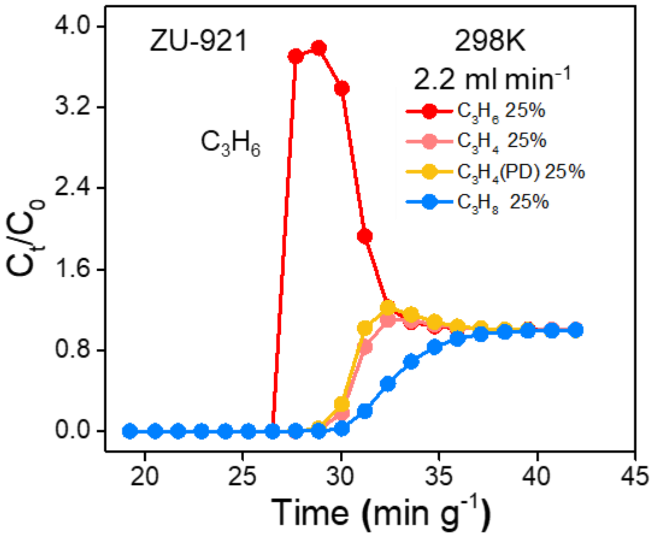

Supplementary Figure 34. Dynamic separation performance. Dynamic breakthrough curves of ZU-921 for C_3H_4/C_3H_4(PD)/C_3H_8/C_3H_6 (25/25/25/25 *v/v/v/v*) mixture in C_t/C_0 under 298 K and 1.0 bar. Source data are provided as a Source Data file.

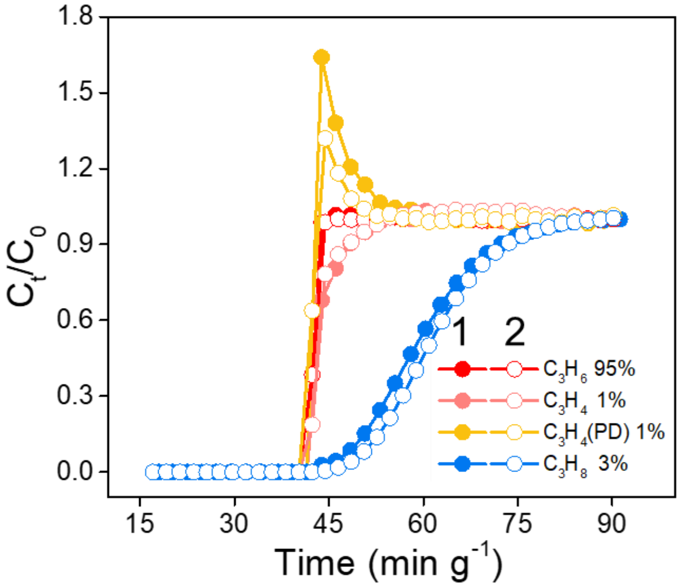

Supplementary Figure 35. Dynamic separation performance. Cycling dynamic breakthrough curves of C_3H_4/C_3H_4(PD)/C_3H_8/C_3H_6 (1/1/3/95 *v/v/v/v*) mixture in C_t/C_0 on FDMOF-2 under 298 K and 1.0 bar. (column: 0.46 cm × 15 cm, 1.29 g, flow rate: 2.2 mL min^{-1})

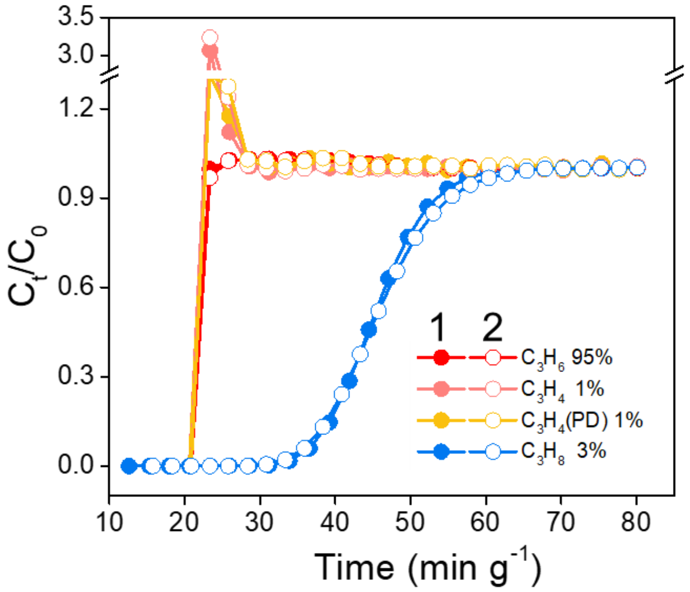

Supplementary Figure 36. Dynamic separation performance. Cycling dynamic breakthrough curves of C_3H_4/C_3H_4(PD)/C_3H_8/C_3H_6 (1/1/3/95 *v/v/v/v*) mixture in C_t/C_0 on ZU-922 under 298 K and 1.0 bar. (column: 0.46 cm × 15 cm, 1.04 g, flow rate: 2.2 mL min^{-1})

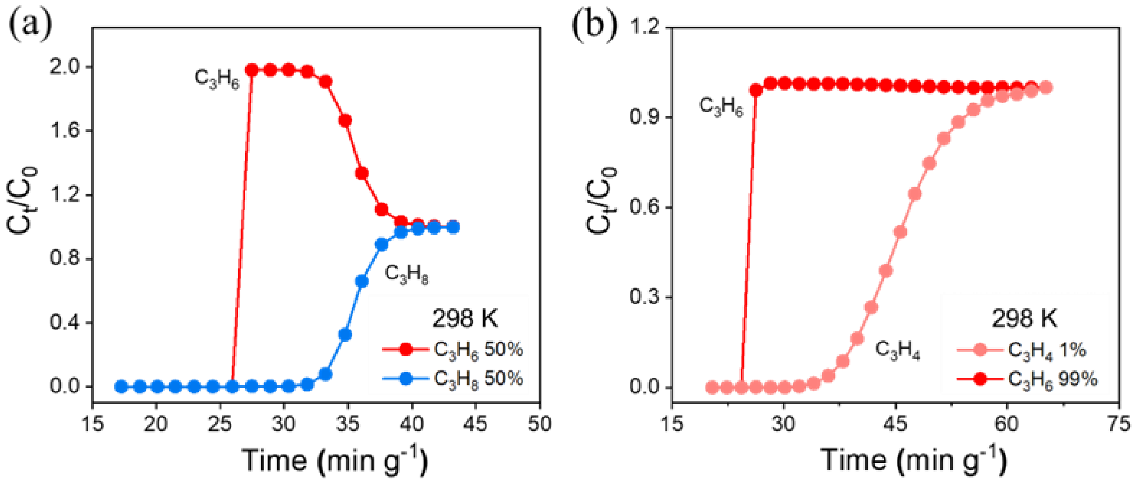

Supplementary Figure 37. Dynamic separation performance. Dynamic breakthrough curves of (a) C_3H_8/C_3H_6 (50/50 *v/v*) and (b) C_3H_4/C_3H_6 (1/99 *v/v*) in C_t/C_0 at 298 K on ZU-921. (column: 0.46 cm × 15 cm, 1.33 g, flow rate: 2.2 mL min^{-1}) Source data are provided as a Source Data file.

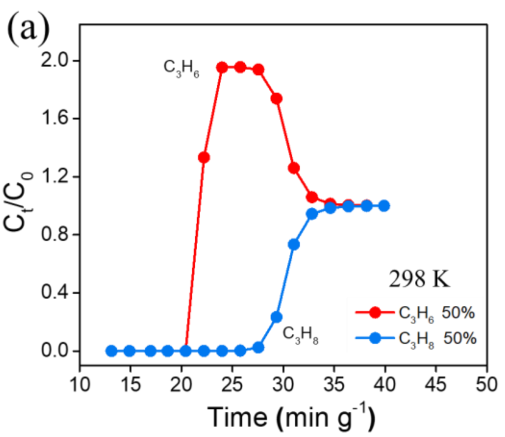

Supplementary Figure 38. Dynamic separation performance. Dynamic breakthrough curves of (a) C_3H_8/C_3H_6 (50/50 *v/v*) and (b) C_3H_4/C_3H_6 (1/99 *v/v*) at 298 K on ZU-922. (column: 0.46 cm × 15 cm, 1.04 g, flow rate: 2.2 mL min^{-1}) Source data are provided as a Source Data file.

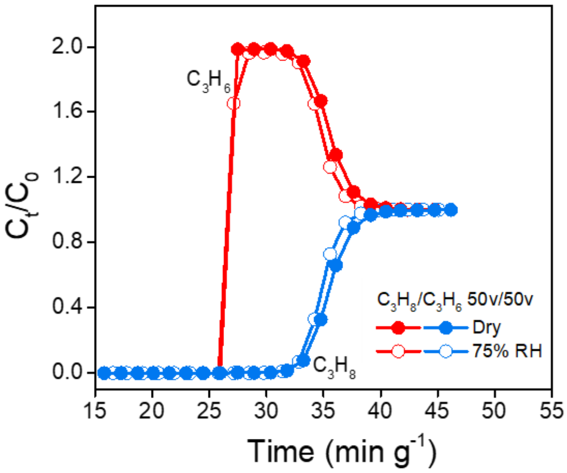

Supplementary Figure 39. Dynamic separation performance. Dynamic breakthrough curves of C_3H_8/C_3H_6 (50/50 *v/v*) with (hollow) or without water vapor (solid) at 298 K on ZU-921. (column: 0.46 cm × 15 cm, 1.33 g, flow rate: 2.2 mL min^{-1})

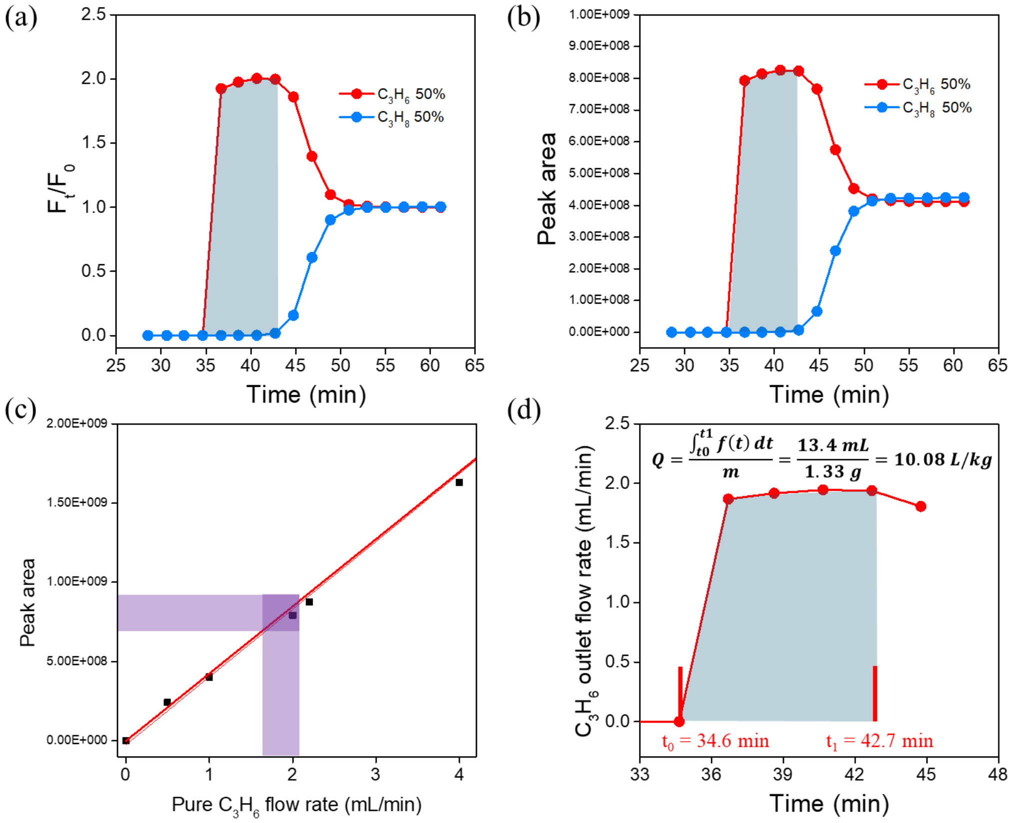

Supplementary Figure 40. Dynamic separation performance. The breakthrough curves of ZU-921 (0.46 cm × 15 cm, 1.33 g) and its C_3H_6 productivity. (a) Breakthrough curve of C_3H_8/C_3H_6 (50/50 *v/v*) mixture at the constant inlet flow rate of 2.2 mL/min under 298 K (F_t and F_0 are the flow rates of each gas at the outlet and inlet, respectively), (b) Real-time peak area of each gas during the breakthrough measured by Shimadzu GC2010, (c) Calibration curve of pure C_3H_6 flow rate versus C_3H_6 peak area on the gas chromatogram, (d) Based on calibration curve between flow rate and C_3H_6 peak area, the real-time outlet C_3H_6 flow rate could be calculated, and the corresponding C_3H_6 productivity could be calculated, around 10.08 L/kg. Source data are provided as a Source Data file.

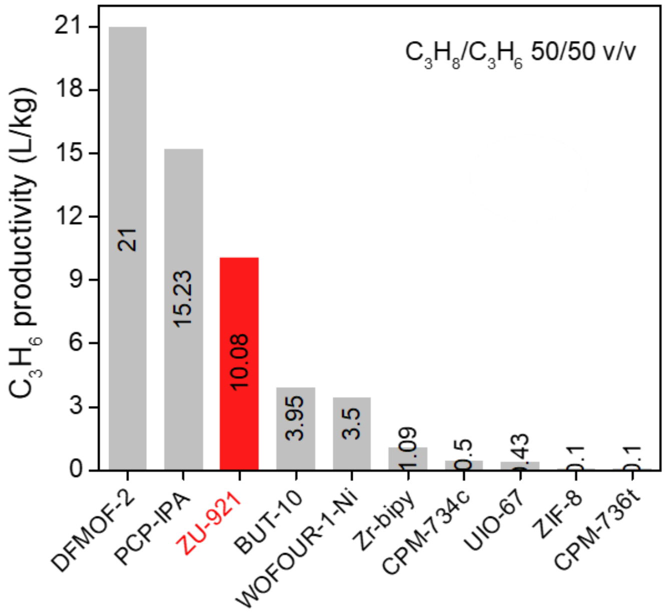

Supplementary Figure 41. Comparison of Olefins productivity. Comparison of C_3H_6 productivity (99.5%) on ZU-921 with reported benchmark materials for C_3H_8/C_3H_6 (50/50 *v/v*) mixture.

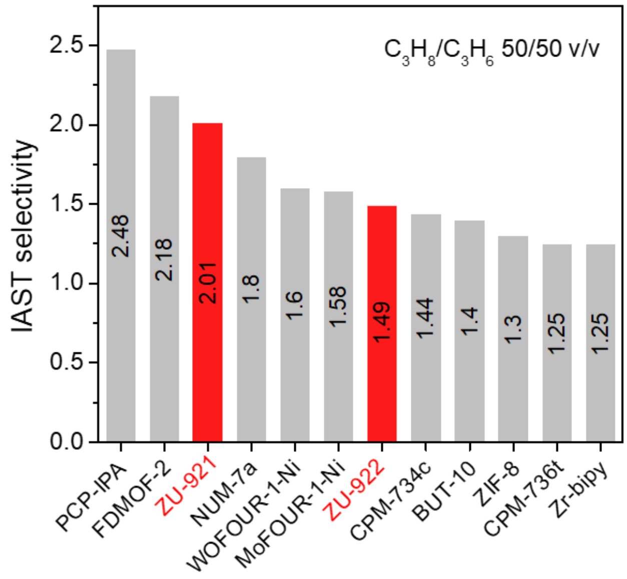

Supplementary Figure 42. Comparison of C_3H_8/C_3H_6 IAST selectivity. Comparison plot of C_3H_8/C_3H_6 IAST selectivity among reported benchmark materials.

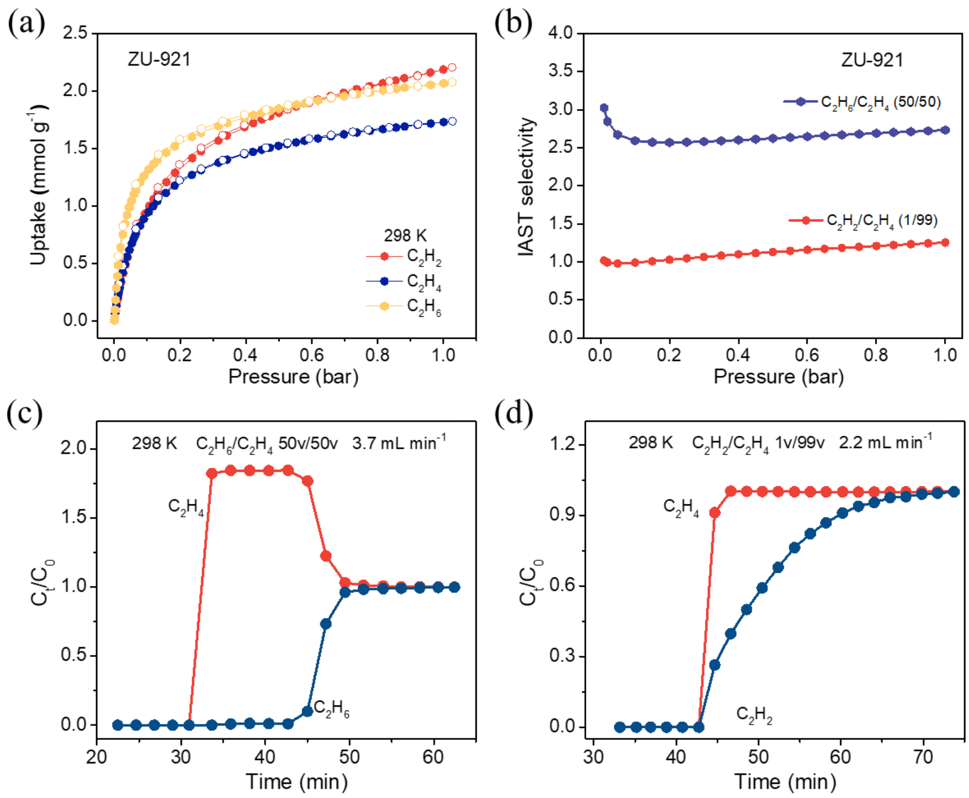

Supplementary Figure 43. Adsorption and separation performance. (a) The C_2H_2, C_2H_4, and C_2H_6 adsorption isotherms and (b) IAST selectivity of C_2H_2/C_2H_4 (1/99) and C_2H_6/C_2H_4 (50/50) mixtures; and dynamic breakthrough curves of (c) C_2H_6/C_2H_4 (50/50 v/v) with the flow rate of 3.7 mL min^{-1} and (d) C_2H_2/C_2H_4 (1/99 v/v) with the flow rate of 2.2 mL min^{-1} at 298 K on ZU-921 (column: 0.46 cm × 15 cm, 1.33 g). Source data are provided as a Source Data file.

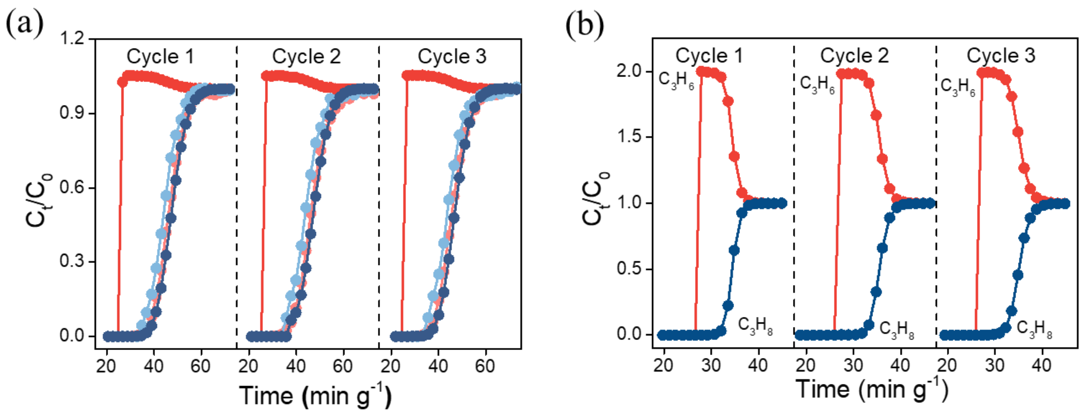

Supplementary Figure 44. Recycling stability tests of ZU-921. Recycling breakthrough tests for C_3H_4/C_3H_4(PD)/C_3H_8/C_3H_6 (1/1/3/95 *v/v/v/v*, flow rate: 2.2 mL min^{-1}) and C_3H_8/C_3H_6 (50/50 *v/v*, flow rate: 2.2 mL min^{-1}) mixture with ZU-921 under 298 K and 1.0 bar (column: 0.46 cm × 15 cm, 1.33 g).

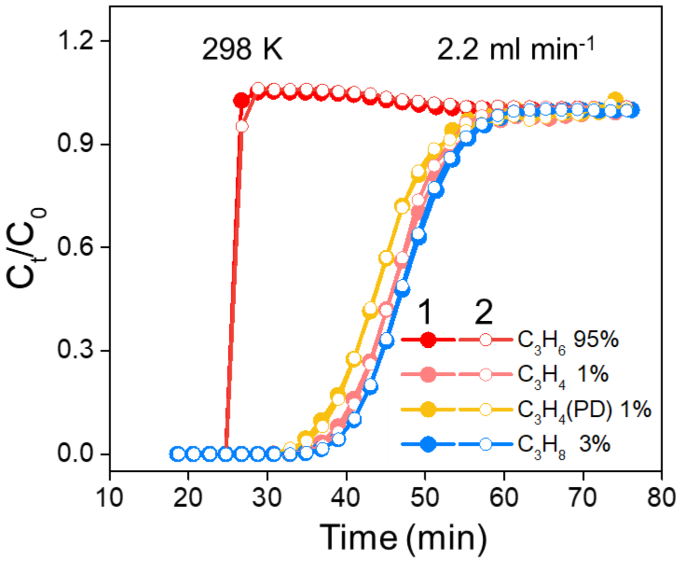

Supplementary Figure 45. Regeneration test. The breakthrough curves of ZU-921 after regeneration with the N_2 flow rate of 10 mL/min at 353 K for 6 hours (close: the first breakthrough curve, open: after regeneration; column: 0.46 cm × 15 cm, 1.33 g, flow rate: 2.2 mL min^{-1})

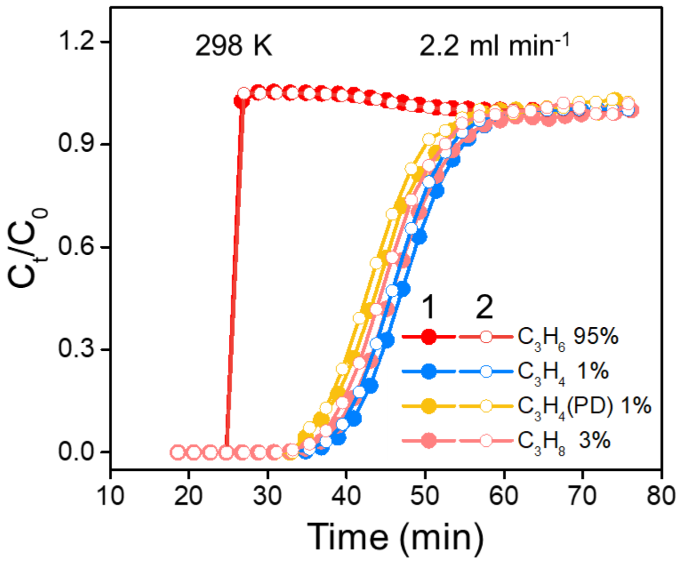

Supplementary Figure 46. Regeneration test. The breakthrough curves of ZU-921 after regeneration under high vacuum (< 1.0 ×10^{-5} mmHg) and 373 K for 6 hours (close: the first breakthrough curve, open: after regeneration; column: 0.46 cm × 15 cm, 1.33 g, flow rate: 2.2 mL min^{-1})

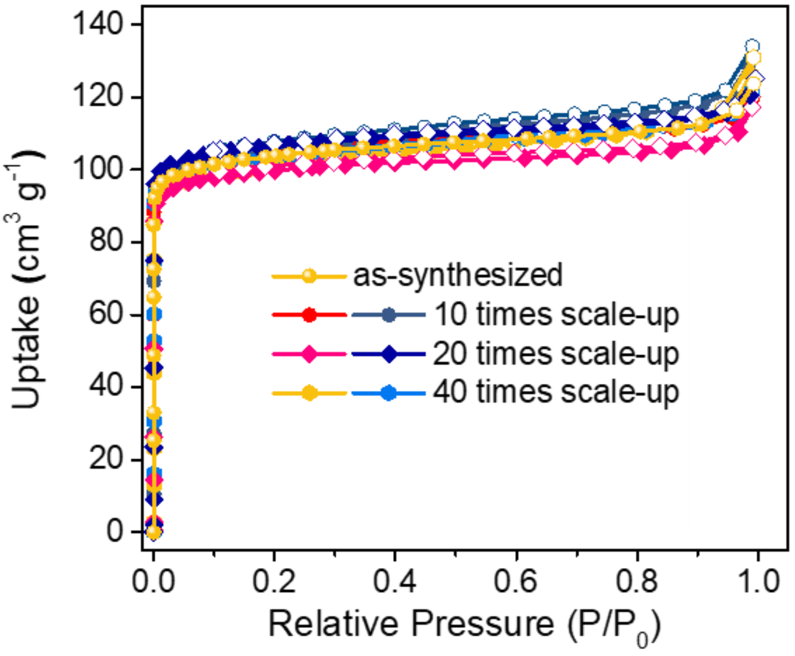

Supplementary Figure 47. Scale-up production consistency. The 77 K N_2 isotherms of scale-up ZU-921.

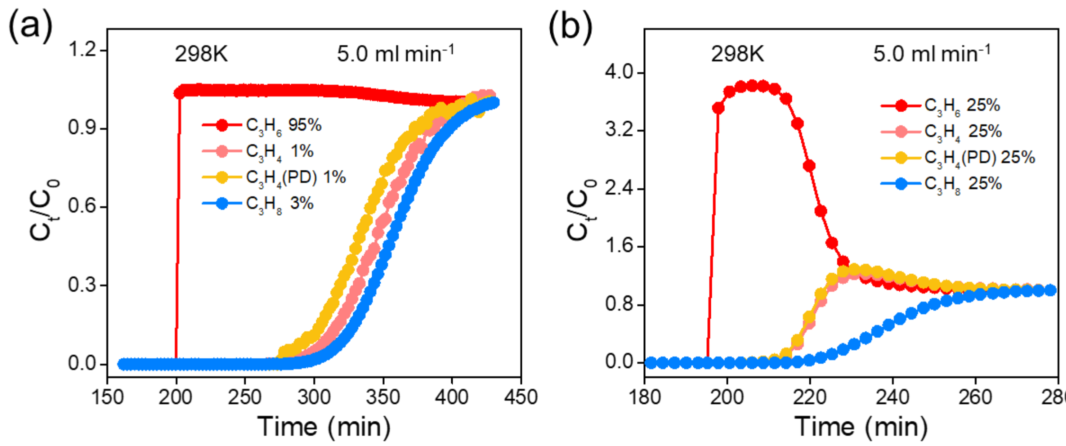

Supplementary Figure 48. Dynamic separation performance. Dynamic breakthrough curves of ZU-921 for (a) C_3H_4/C_3H_4(PD)/C_3H_8/C_3H_6 (1/1/3/95 *v/v/v/v*) mixture (b) C_3H_4/C_3H_4(PD)/C_3H_8/C_3H_6 (25/25/25/25 *v/v/v/v*) mixture in C_t/C_0 under 298 K. (column: 1.0 cm × 50 cm, 20.5 g, flow rate: 5.0 mL min^{-1}) Source data are provided as a Source Data file.

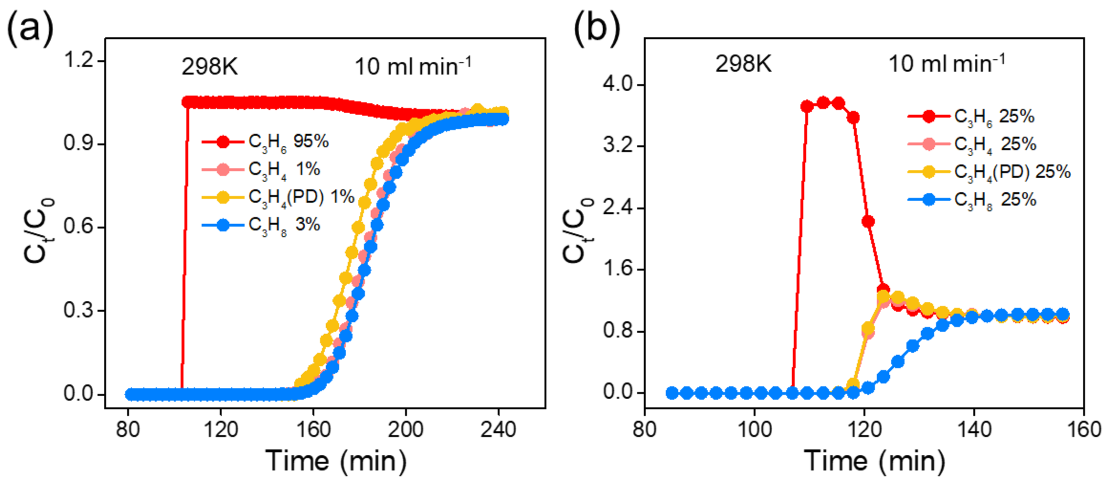

Supplementary Figure 49. Dynamic separation performance. Dynamic breakthrough curves of ZU-921 for (a) C_3H_4/C_3H_4(PD)/C_3H_8/C_3H_6 (1/1/3/95 *v/v/v/v*) mixture (b) C_3H_4/C_3H_4(PD)/C_3H_8/C_3H_6 (25/25/25/25 *v/v/v/v*) mixture in C_t/C_0 under 298 K. (column: 1.0 cm × 50 cm, 20.5 g, flow rate: 10.0 mL min^{-1}) Source data are provided as a Source Data file.

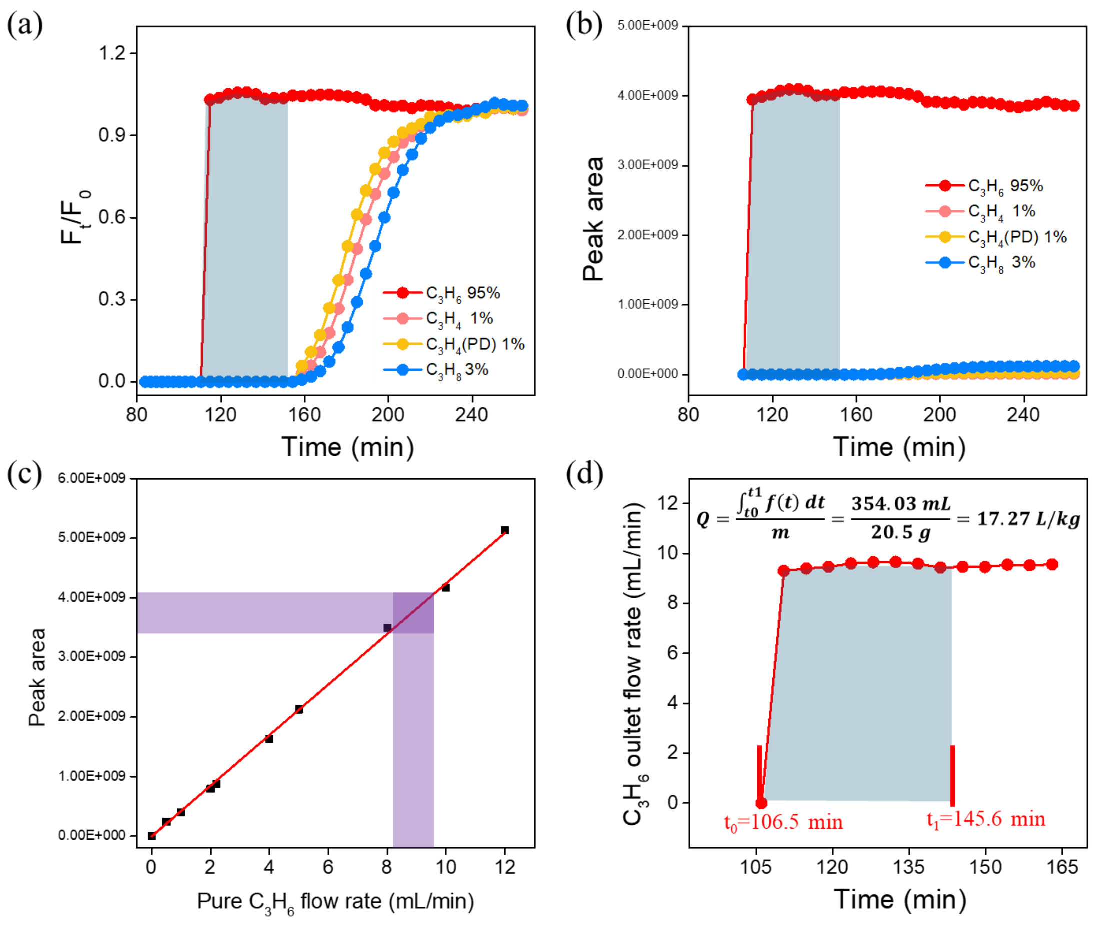

Supplementary Figure 50. Dynamic separation performance. The breakthrough curves of ZU-921 (1.0 cm x 50 cm, 20.5 g) and its C_3H_6 productivity. (a) Breakthrough curve of C_3H_4/C_3H_4(PD)/C_3H_8/C_3H_6 (1/1/3/95 *v/v/v/v*) mixture at the constant inlet flow rate of 10 mL/min under 298 K (F_t and F_0 are the flow rates of each gas at the outlet and inlet, respectively), (b) Real-time peak area of each gas during the breakthrough measured by Shimadzu GC2010, (c) Calibration curve of pure C_3H_6 flow rate versus C_3H_6 peak area on the gas chromatogram, (d) Based on calibration curve between flow rate and C_3H_6 peak area, the real-time outlet C_3H_6 flow rate could be calculated, and the corresponding C_3H_6 productivity could be calculated, around 17.27 L/kg. Source data are provided as a Source Data file.

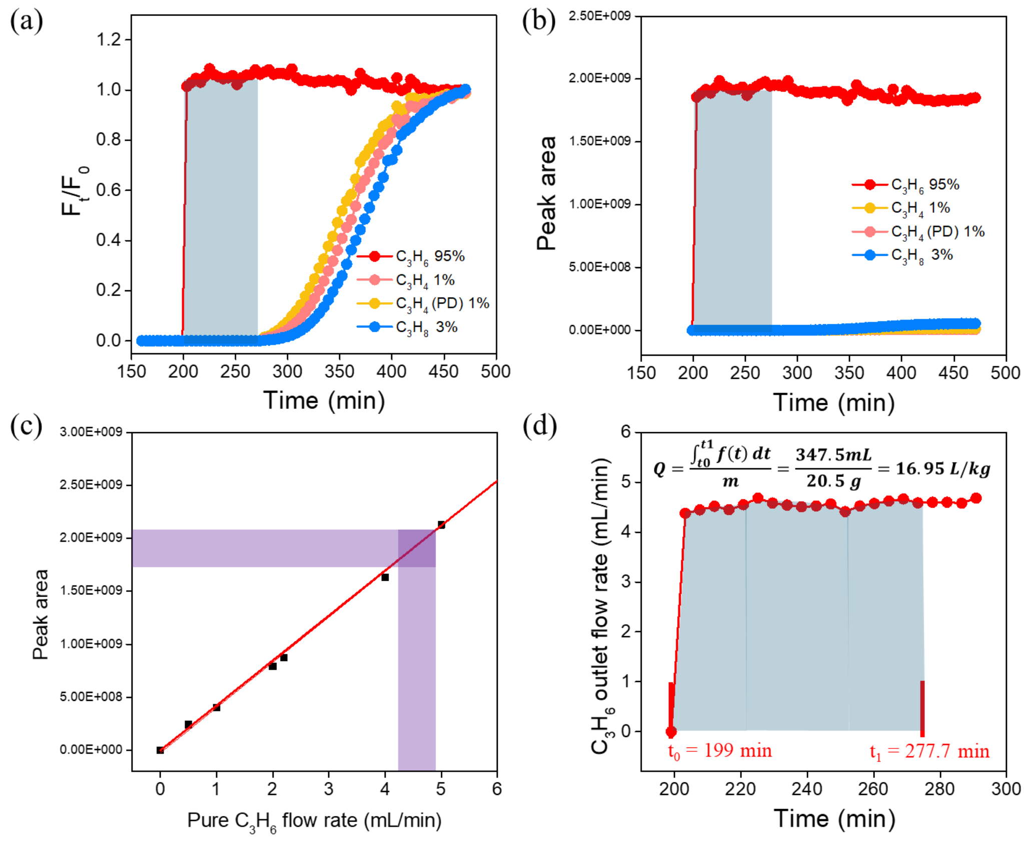

Supplementary Figure 51. Dynamic separation performance. The breakthrough curves of ZU-921 (1.0 cm x 50 cm, 20.5 g) and its C_3H_6 productivity. (a) Breakthrough curve of C_3H_4/C_3H_4(PD)/C_3H_8/C_3H_6 (1/1/3/95 *v/v/v/v*) mixture at the constant inlet flow rate of 5.0 mL/min under 298 K (F_t and F_0 are the flow rates of each gas at the outlet and inlet, respectively), (b) Real-time peak area of each gas during the breakthrough measured by Shimadzu GC2010, (c) Calibration curve of pure C_3H_6 flow rate versus C_3H_6 peak area on the gas chromatogram, (d) Based on calibration curve between flow rate and C_3H_6 peak area, the real-time outlet C_3H_6 flow rate could be calculated, and the corresponding C_3H_6 productivity could be calculated, around 16.95 L/kg. Source data are provided as a Source Data file.

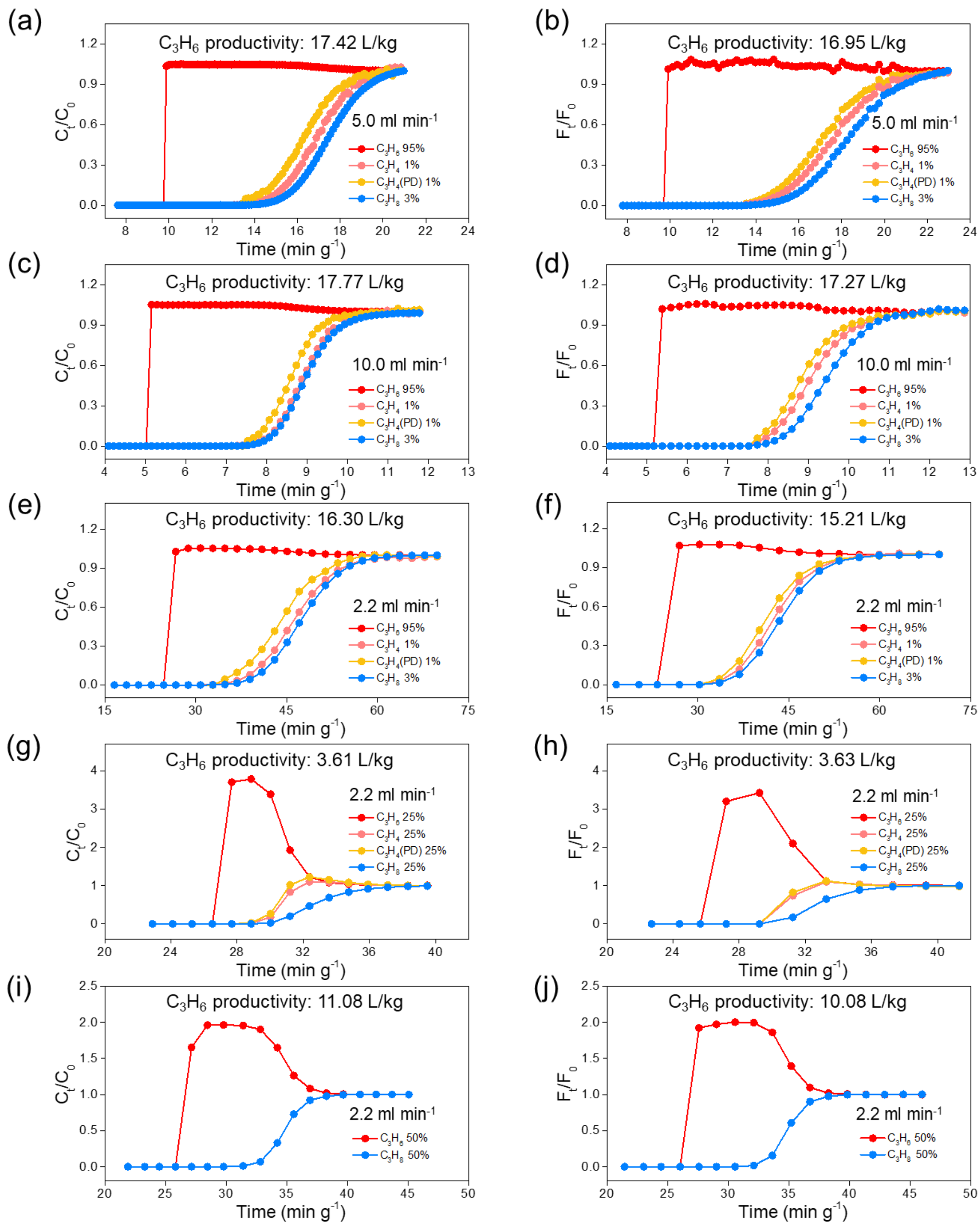

Supplementary Figure 52. Dynamic separation performance. (a) , (c) , (e) , (g) , (i) Dynamic breakthrough curves of ZU-921 under 298 K and 1.0 bar for different C3 mixtures in C_t/C_0 (C_t and C_0 are the relative concentration of each gas at the outlet and inlet, respectively) ; (b) , (d) , (f) , (h) , (j) Dynamic breakthrough curves of ZU-921 under 298 K and 1.0 bar for different C3 mixtures in F_t/F_0 (F_t and F_0 are the flow rates of each gas at the outlet and inlet, respectively).

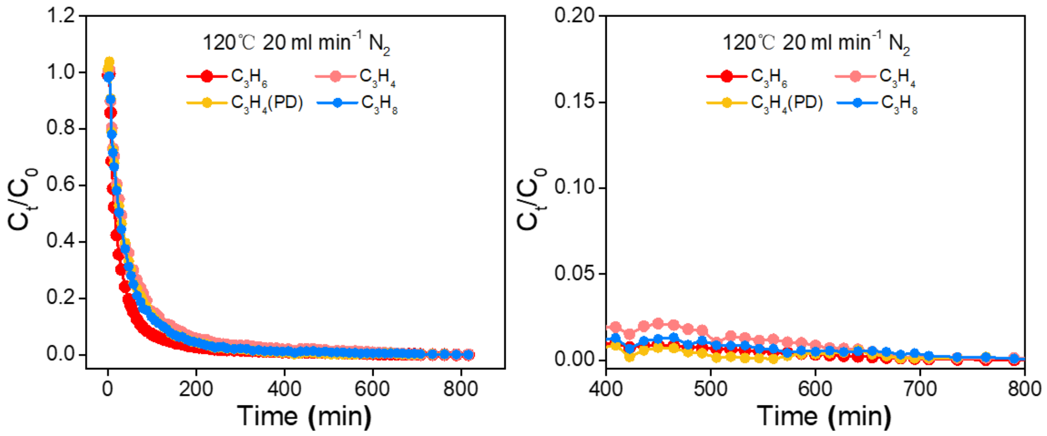

Supplementary Figure 53. Regeneration curves. Regeneration curves with a N_2 flow rate of 20 mL min^{-1} at 393 K after breakthrough measurements of C_3H_4/C_3H_4(PD)/C_3H_8/C_3H_6 (1/1/3/95 *v/v/v/v*) mixture at the flow rate of 10 mL min^{-1}. (column: 1.0 cm × 50 cm, 20.5 g, the desorption condition could be equivalent to using about 16 L and 393 K of hot nitrogen for the regeneration of the adsorption column, and the energy consumption included the energy to heat nitrogen from 298 K to 393 K and the energy to recover the nitrogen) Source data are provided as a Source Data file.

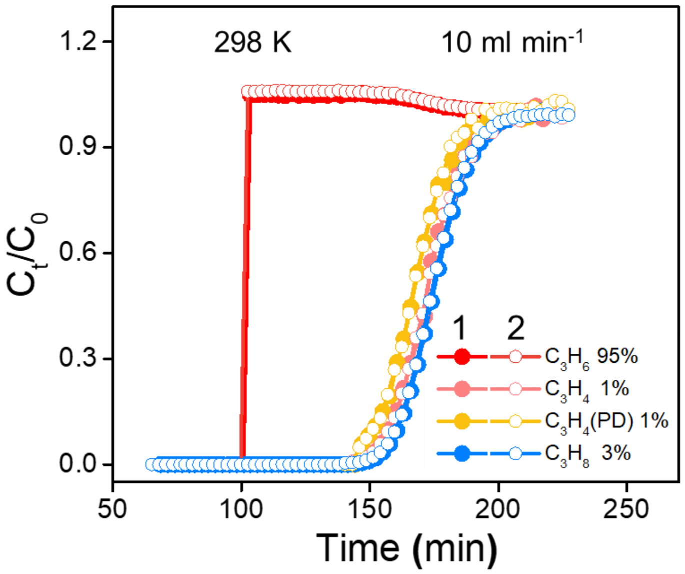

Supplementary Figure 54. Regeneration condition. The breakthrough curves of ZU-921 after regeneration with the N_2 flow rate of 20 mL/min at 393 K. (close: the first breakthrough curve, open: after regeneration; column: 1.0 cm × 50 cm, 20.5 g, flow rate: 10 mL min^{-1}) Source data are provided as a Source Data file.

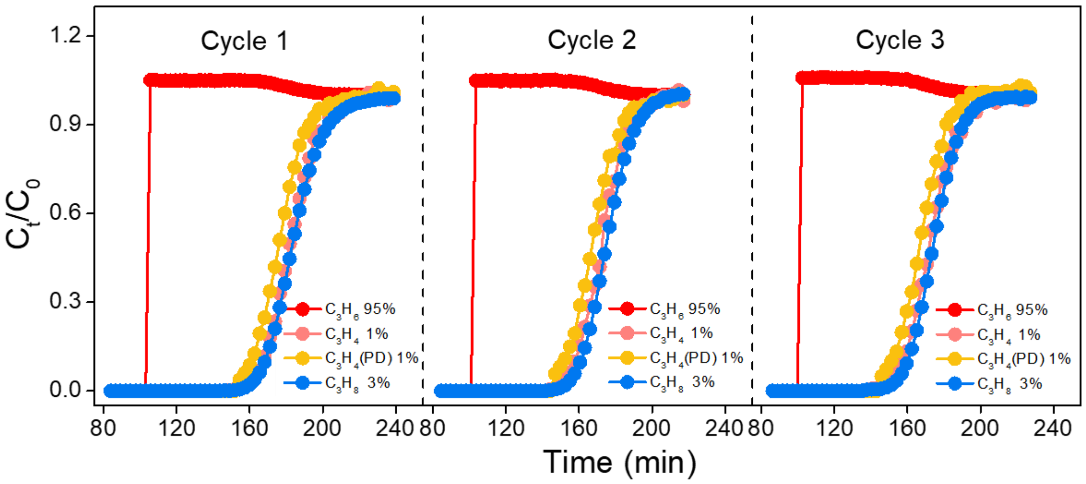

Supplementary Figure 55. Recycling stability. Recycling breakthrough tests for C_3H_4/C_3H_4(PD)/C_3H_8/C_3H_6 (1/1/3/95 *v/v/v/v*) mixture in C_t/C_0 with ZU-921 under 298 K and 1.0 bar. (column: 1.0 cm × 50 cm, 20.5 g, flow rate: 10 mL min^{-1}) Source data are provided as a Source Data file.

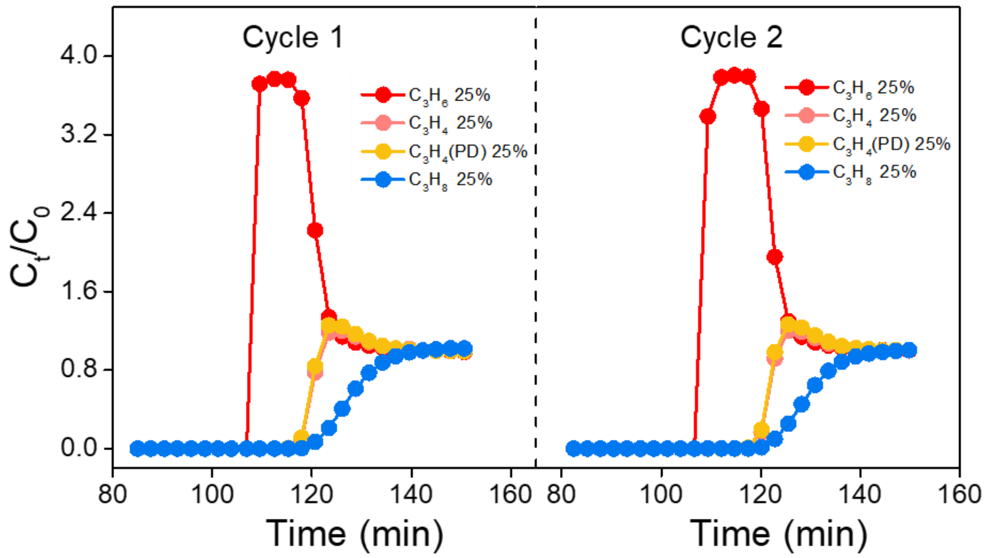

Supplementary Figure 56. Recycling stability. Recycling breakthrough tests for C_3H_4/C_3H_4(PD)/C_3H_8/C_3H_6 (25/25/25/25 *v/v/v/v*) mixture in C_t/C_0 with ZU-921 under 298 K and 1.0 bar. (column: 1.0 cm × 50 cm, 20.5 g, flow rate: 10 mL min^{-1}) Source data are provided as a Source Data file.

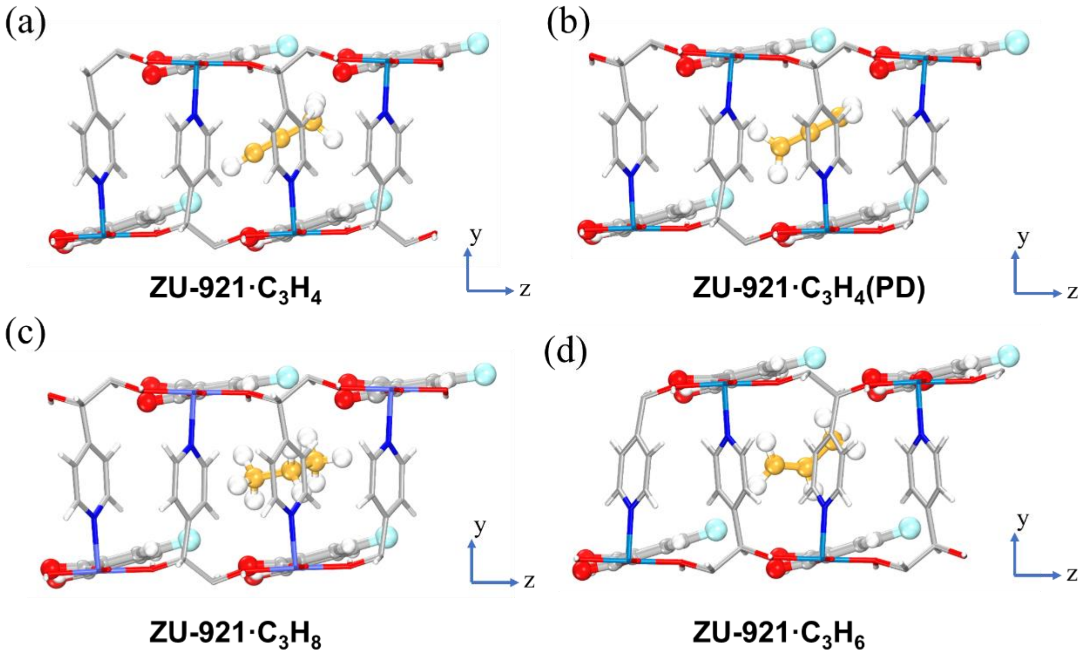

Supplementary Figure 57. Adsorption sites. DFT-D calculated preferable adsorption positions for C3 gases in ZU-921 (a) C_3H_4 (b) C_3H_4 (PD) (c) C_3H_8 (d) C_3H_6 adsorption positions in ZU-921 (Framework: C, grey-80%; H, white; N, blue; O, red; Co, light blue; Gas: C, orange; H, white).

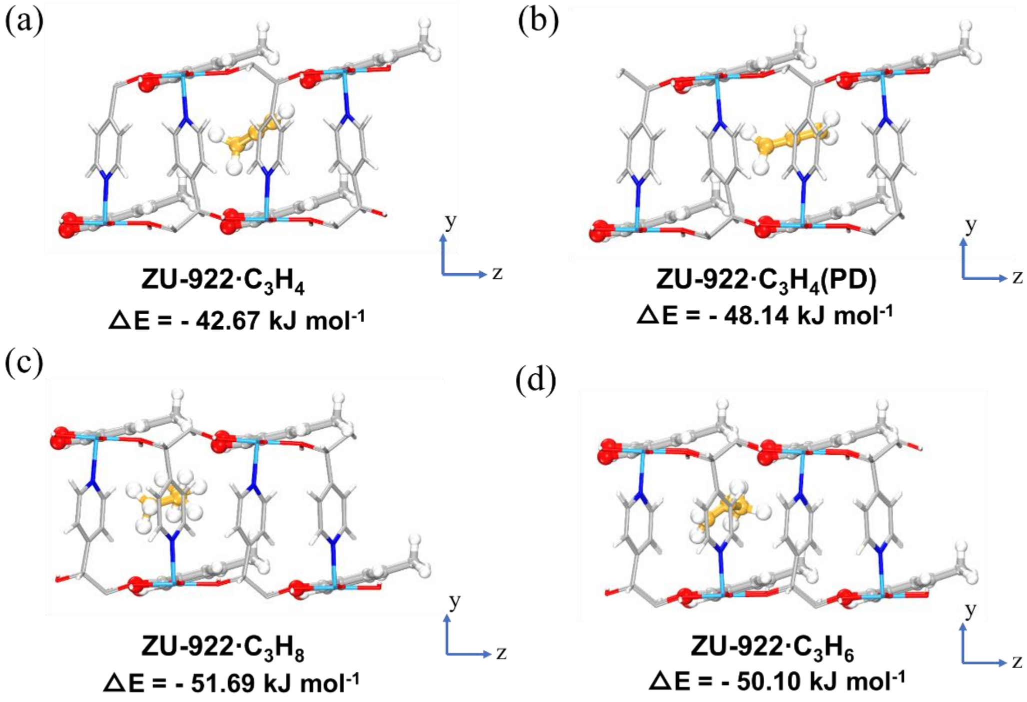

Supplementary Figure 58. Adsorption sites. DFT-D calculated preferable adsorption positions for C3 gases in ZU-922 (a) C_3H_4 (b) C_3H_4 (PD) (c) C_3H_8 (d) C_3H_6 adsorption positions in ZU-922 (Framework: C, grey-80%; H, white; N, blue; O, red; Co, light blue; Gas: C, orange; H, white).

Supplementary Tables

Supplementary Table 1. Physical properties of C_3H_4, C_3H_4 (PD), C_3H_6, and C_3H_8.

Adsorbate	Kinetic diameter (Å)	Polarizability (*10^{-25}/cm^3)	Dipole moment (*10^{-18}/esu cm^2)	Quadruple moment (D*A)	vdW surface areas (Å^2)	boiling point (°C)
C_3H_4	4.2	55.5	0.834	60.352	90.2	-23.3
C_3H_4 (PD)	4.2	56.9	0.00025	70.365	-	-34.5
C_3H_6	4.68	62.6	0.425	67.574	97.3	-47.4
C_3H_8	4.3-5.12	62.9-63.7	0.093	66.907	102.2	-42.1

1D*A= 3.33564*$10^{\wedge -37}$ C*cm^2

Supplementary Table 2. Lattice parameters of the refined structures of ZU-921 and ZU-922.

Unit cell parameters	ZU-921	ZU-922
Formula	$C_{40}H_{30}N_4O_{12}F_2Co_2$	$C_{42}H_{40}N_4O_{12}Co_2$
Formula weight	914.54	910.64
Crystal system	orthorhombic	orthorhombic
Space group	AMM2	AMM2
a (Å)	11.162972	11.050116
b (Å)	13.835723	13.715282
c (Å)	7.657443	7.637173
α (°)	90	90
β (°)	90	90
γ (°)	90	90
V (Å^3)	1182.68	1157.45
D_{calcd} (g cm^{-3})	1.2841	1.3007
Rp	0.0143	0.0142
R_{wp}	0.0298	0.0270
CCDC number	2294207	2294206

Supplementary Table 3. Textural parameters summary of ZU-921 to ZU-924 and PCP-BDC

Sample	Adsorbate	S_{BET} [a] ($m^2 g^{-1}$)	$S_{Langmuir}$ [b] ($m^2 g^{-1}$)	V_{Total} [c] ($cm^3 g^{-1}$)
ZU-921	195 K CO_2	/	356.6	0.146
ZU-922		/	369.5	0.150
ZU-921	77 K N_2	425.5	/	0.184
ZU-923		510.7	/	0.230
ZU-924		402.1	/	0.199
PCP-BDC		492.4	/	0.245

[a]The specific surface area was determined by the BET or Langmuir equation (P/P_0 = 0.005−0.3). [b]Total pore volume at P/P_0 = 0.99.

Supplementary Table 4. Summary of separation metrics of benchmark materials for C3 separations under 1.0 bar and 298 K

Samples	IAST selectivity C_3H_4/C_3H_6 (1/99 v/v)	IAST selectivity C_3H_4 (PD)/C_3H_6 (1/99 v/v)	IAST selectivity C_3H_8/C_3H_6 (3/97 v/v)	Reference
NKMOF-1-Ni	653.8	119.1	0.67	29
NbOFFIVE-2-Cu-i	27.6	22.5	0.52	28
SIFSIX-3-Ni	292.3	4.5	0.17	23
ZU-33	195.9	150.6	0.14	44
DFMOF-2	1.34	1.08	1.48	32
Ni(ADC)(TED)$_{0.5}$	0.55	1.06	1.57	45
**PCP-BDC**	**0.48**	**0.49**	**1.75**	**This work**
**ZU-924**	**1.67**	**1.38**	**0.5**	**This work**
**ZU-922**	**0.52**	**0.75**	**1.49**	**This work**
**ZU-923**	**1.49**	**1.77**	**1.50**	**This work**
**ZU-921**	**2.17**	**2.03**	**2.03**	**This work**

Supplementary Table 5. Summary of separation metrics of ZU-921 for C3 separations under 1.0 bar

Samples	Temperature	IAST selectivity C_3H_8/C_3H_6 (3/97 v/v)	IAST selectivity C_3H_4/C_3H_6 (1/99 v/v)	IAST selectivity C_3H_4 (PD)/C_3H_6 (1/99 v/v)
ZU-921	273 K	2.02	2.08	2.13
	298 K	2.03	2.17	2.03
	313 K	1.84	1.95	1.82

Supplementary Table 6. Dual-site Langmuir-Freundlich parameters of different gases on ZU-921 to ZU-924 and PCP-BDC at 298 K

		Site I			Site II			
	Gas	q_{sat1} mol kg^{-1}	b_1 kPa^{-1}	V_1 dimensionless	q_{sat2} mol kg^{-1}	b_2 kPa^{-1}	V_2 dimensionless	R^2
ZU-921	C_3H_4	1.24E+00	9.65E-02	5.46E-01	1.62E+00	1.91E+00	8.86E-01	0.9998
	C_3H_4 (PD)	1.16E+00	1.82E-01	5.84E-01	1.34E+00	2.85E+00	9.78E-01	0.9999
	C_3H_6	1.05E+00	1.85E-01	5.83E-01	1.10E+00	3.05E+00	9.78E-01	0.9999
	C_3H_8	1.18E+00	6.09E+00	1.09E+00	9.94E-01	2.64E-01	5.58E-01	0.9999
ZU-922	C_3H_4	2.01E+00	8.38E-03	7.85E-01	1.34E+00	1.73E+00	8.91E-01	0.9998
	C_3H_4 (PD)	1.01E+00	1.53E-01	5.62E-01	1.12E+00	3.06E+00	9.85E-01	0.9999
	C_3H_6	1.06E+00	5.48E+00	9.91E-01	8.51E-01	2.51E-01	5.54E-01	0.9999
	C_3H_8	1.04E+00	1.72E+01	1.32E+00	7.93E-01	3.83E-01	5.75E-01	0.9997
PCP-BDC	C_3H_4	2.12E+00	8.10E-02	4.70E-01	1.44E+00	6.08E-01	1.06E+00	0.9999
	C_3H_4 (PD)	3.88E+00	3.23E-02	4.40E-01	1.12E+00	4.76E-01	1.17E+00	0.9999
	C_3H_6	2.70E+00	5.54E-01	3.99E-01	8.51E-01	1.96E+00	1.52E+00	0.9999
	C_3H_8	1.87E+00	7.12E-04	1.32E+00	1.12E+00	1.08E+00	8.94E-01	0.9999
ZU-923	C_3H_4	1.59E+00	9.66E-02	5.98E-01	1.83E+00	1.34E+00	9.92E-01	0.9999
	C_3H_4 (PD)	1.56E+00	1.15E-01	6.11E-01	1.80E+00	1.67E+00	1.07E+00	0.9999
	C_3H_6	8.43E-01	1.89E-02	8.70E-01	1.98E+00	1.21E+00	9.89E-01	0.9999
	C_3H_8	1.75E+00	1.80E+00	1.19E+00	2.92E+01	6.02E-03	1.38E-01	0.9996
ZU-924	C_3H_4	2.50E+00	4.21E-04	1.08E+00	2.15E+00	8.81E-01	6.09E-01	0.9997
	C_3H_4 (PD)	2.85E+00	6.06E-03	6.39E-01	1.74E+00	1.02E+00	8.76E-01	0.9999
	C_3H_6	1.86E+00	1.06E+00	9.09E-01	1.31E-01	5.43E+04	2.75E+00	0.9999
	C_3H_8	2.71E+00	9.79E-07	2.11E+00	1.75E+00	4.87E-01	8.43E-01	0.9998

Supplementary Table 7. Dual-site Langmuir-Freundlich parameters of different gases on ZU-921 and ZU-922 at 273 K

	Gas	Site I			Site II			R^2
		q_{sat1} mol kg^{-1}	b_1 kPa^{-1}	V_1 dimensionless	q_{sat2} mol kg^{-1}	b_2 kPa^{-1}	V_2 dimensionless	
ZU-921	C_3H_4	1.24E+00	9.65E-02	5.46E-01	1.62E+00	1.91E+00	8.86E-01	0.9998
	C_3H_4 (PD)	1.16E+00	1.82E-01	5.84E-01	1.34E+00	2.85E+00	9.78E-01	0.9999
	C_3H_6	1.05E+00	1.85E-01	5.83E-01	1.10E+00	3.05E+00	9.78E-01	0.9999
	C_3H_8	1.18E+00	6.09E+00	1.09E+00	9.94E-01	2.64E-01	5.58E-01	0.9999
ZU-922	C_3H_4	2.01E+00	8.38E-03	7.85E-01	1.34E+00	1.73E+00	8.91E-01	0.9998
	C_3H_4 (PD)	1.01E+00	1.53E-01	5.62E-01	1.12E+00	3.06E+00	9.85E-01	0.9999
	C_3H_6	1.06E+00	5.48E+00	9.91E-01	8.51E-01	2.51E-01	5.54E-01	0.9999
	C_3H_8	1.04E+00	1.72E+01	1.32E+00	7.93E-01	3.83E-01	5.75E-01	0.9997

Supplementary Table 8. Dual-site Langmuir-Freundlich parameters of different gases on ZU-921 and ZU-922 at 313 K

	Gas	Site I			Site II			R^2
		q_{sat1} mol kg^{-1}	b_1 kPa^{-1}	V_1 dimensionless	q_{sat2} mol kg^{-1}	b_2 kPa^{-1}	V_2 dimensionless	
ZU-921	C_3H_4	1.24E+00	9.65E-02	5.46E-01	1.62E+00	1.91E+00	8.86E-01	0.9998
	C_3H_4 (PD)	1.16E+00	1.82E-01	5.84E-01	1.34E+00	2.85E+00	9.78E-01	0.9999
	C_3H_6	1.05E+00	1.85E-01	5.83E-01	1.10E+00	3.05E+00	9.78E-01	0.9999
	C_3H_8	1.18E+00	6.09E+00	1.09E+00	9.94E-01	2.64E-01	5.58E-01	0.9999
ZU-922	C_3H_4	2.01E+00	8.38E-03	7.85E-01	1.34E+00	1.73E+00	8.91E-01	0.9998
	C_3H_4 (PD)	1.01E+00	1.53E-01	5.62E-01	1.12E+00	3.06E+00	9.85E-01	0.9999
	C_3H_6	1.06E+00	5.48E+00	9.91E-01	8.51E-01	2.51E-01	5.54E-01	0.9999
	C_3H_8	1.04E+00	1.72E+01	1.32E+00	7.93E-01	3.83E-01	5.75E-01	0.9997

Supplementary Table 9. Dual-site Langmuir-Freundlich parameters of different C3 gases for the comparison materials at 298 K

Samples	Gas	Site I			Site II			R^2
		q_{sat1} mol kg^{-1}	B_1 kPa^{-1}	V_1 dimensionless	q_{sat2} mol kg^{-1}	B_2 kPa^{-1}	V_2 dimensionless	
DFMOF-2	C_3H_4	2.52E+00	3.73E-02	8.54E-01	2.72E+00	1.11E-01	1.50E+00	0.99999
	C_3H_4 (PD)	1.57E+01	3.00E-03	5.54E-01	3.40E+00	1.18E-01	1.20E+00	0.99999
	C_3H_6	3.22E+00	1.16E-01	8.57E-01	8.95E-01	1.05E-01	2.03E+00	0.99994
	C_3H_8	1.03E+00	3.42E-05	3.08E+00	3.03E+00	2.54E-01	1.26E+00	0.99975
Ni(ADC)(ted)$_{0.5}$	C_3H_4	2.19E+00	1.45E+01	3.28E+00	2.33E+00	5.43E-02	6.82E-01	0.99791
	C_3H_4 (PD)	2.43E+00	1.14E+01	1.23E+00	2.19E+00	8.91E-03	8.64E-01	0.99845
	C_3H_6	1.96E-01	2.32E-04	1.99E+00	2.58E+00	7.08E+00	1.05E+00	0.99835
	C_3H_8	1.50E+01	2.50E-02	9.83E-02	2.14E+00	2.42E+01	1.52E+00	0.99923
NbOFFIVE-2-Cu-i	C_3H_4	2.01E+00	3.53E+01	2.65E+00	1.76E+00	2.20E-01	7.54E-01	0.99652
	C_3H_4 (PD)	1.81E+00	4.08E+01	2.77E+00	1.63E+00	2.57E-01	7.98E-01	0.99789
	C_3H_6	1.65E+00	5.14E-02	1.96E+00	1.16E+00	2.16E-03	1.70E+00	0.99921
	C_3H_8	1.42E+00	8.49E-03	2.15E+00	1.61E+00	1.30E-03	1.64E+00	0.99972
SIFSIX-3-Ni	C_3H_4	1.15E+00	5.10E-04	1.30E+00	2.77E+00	1.47E+02	1.47E+00	0.99528
	C_3H_4 (PD)	1.83E+00	1.28E-01	8.39E+00	6.65E-01	2.05E-01	1.21E+00	0.99825
	C_3H_6	7.72E-01	6.15E-03	2.38E+00	2.14E+00	7.40E-02	1.00E+00	0.99995
	C_3H_8	2.02E+00	2.00E-05	2.77E+00	2.54E+00	2.79E-02	2.53E-01	0.99862
NKMOF-1-Ni	C_3H_4	8.98E+01	6.33E-03	2.81E-01	1.33E+00	8.83E+03	1.52E+00	0.99689
	C_3H_4 (PD)	9.45E+01	3.39E-03	3.30E-01	1.27E+00	3.85E+01	9.53E-01	0.99932
	C_3H_6	2.34E+00	2.32E-02	8.03E-01	1.02E+00	2.27E-01	9.12E-01	0.99993
	C_3H_8	4.22E+00	2.30E-03	8.39E-01	1.20E+00	2.14E-01	1.09E+00	0.99991
ZU-33	C_3H_4	2.58E+00	7.17E+00	2.17E+00	2.11E+00	4.94E-02	5.93E-01	0.99457
	C_3H_4 (PD)	1.51E+00	7.76E+04	9.42E+00	1.64E+00	4.74E-01	1.14E+00	0.99741
	C_3H_6	1.37E+00	4.05E-05	3.20E+00	3.90E-02	1.01E+00	1.18E+00	0.99737
	C_3H_8	7.11E-01	7.07E-02	7.03E-01	4.42E-03	8.51E-01	1.03E+00	0.99942

Energy consumption calculation. The energy consumptions are approximately calculated using the Aspen software and do not consider the energy loss. For the adsorptive separation process, we calculated the energy consumption through simulated pressure and temperature swing adsorption process (heat and vacuum at 393 K) and the experimental hot nitrogen purging process (the purge of hot nitrogen at 393 K). 1). The energy consumption of the heat and vacuum at 393 K includes the energy to heat the adsorption bed from 298 K to 393 K and the energy of the vacuum. The energy consumption of the heating adsorption bed from 298 K to 393 K was calculated based on the heat exchanger unit, and the energy of the vacuum was calculated based on the theoretical vacuum pumping power of the pressure swing process using Aspen adsorption. 2). the energy consumption of the purge of hot nitrogen (393 K) includes the energy to heat nitrogen from 298 K to 393 K and the energy to recover the nitrogen (the purity of recovered N_2 is 99.99%). The detailed calculated parameters are derived from the regeneration condition of the breakthrough experiment using the 10-times scale-up column (1.0 cm × 50 cm, 20.5 g) (Figure S39). The energy consumption of heating nitrogen from 298 K to 393 K and recovering the nitrogen were calculated based on the heat exchanger unit and flash tank separation unit, respectively. For the cascade catalytic hydrogenation and distillation process, the energy consumption of catalytic hydrogenation was calculated using the heat exchanger unit of Aspen software based on the real temperature change of the feed and discharge before and after catalytic hydrogenation (315 K to 283 K to 318 K). The energy consumption of distillation for the propylene purification is calculated using the Aspen software with the physical property method being the RK-SOAVE model. The separation conditions referred to the parameters of the distillation of propylene-propane separation in the industrial sector and optimized the optimal theoretical separation energy consumption by adjusting the number of trays, reflux ratio, yield, and purity of propylene.

Supplementary Table 10. The energy consumption for the adsorptive separation of C3 quaternary mixture (C_3H_4/C_3H_4(PD)/C_3H_8/C_3H_6 1v/1v/3v/95v). (Column: 0.1 m× 2.0 m, Material: 8.2 kg, flow rate: 0.44 mol/min, the desorption was evaluated under high-vacuum (<10^{-5} mmHg) and 393 K for 800 min)

	C_3H_6 purity	Heating energy consumption MJ/kg (C_3H_6)	Vacuum energy consumption MJ/kg (C_3H_6)	Total energy consumption MJ/kg(C_3H_6)
1	99.9%	3.7	2.6	6.3
2	99.99%	4.0	2.9	6.9

Supplementary Table 11. The energy consumption for the adsorptive separation of C3 quaternary mixture (C_3H_4/C_3H_4(PD)/C_3H_8/C_3H_6 1v/1v/3v/95v). (The desorption condition is based on the 10-times scale-up column breakthrough experiment: 20 mL/min N_2 at 393 K for 800 min)

	C_3H_6 purity	Heating N_2 energy consumption MJ/kg (N_2)	Recycling N_2 energy consumption MJ/kg (N_2)	Total energy consumption MJ/kg (C_3H_6)
1	99.9%	0.099	0.20	7.8
2	99.99%	0.099	0.20	8.5

Supplementary Table 12. The separation condition and energy consumption for the separation of C3 binary mixture (C_3H_4/C_3H_4(PD)/C_3H_8/C_3H_6 1v/1v/3v/95v) using catalytic hydrogenation and distillation. (Catalytic hydrogenation: temperature: 283 K, feed pressure: 0.58 MPa, processing capacity: 1000 kg/h, Distillation: temperature: 318 K, feed pressure: 1.86 MPa, processing capacity: 1000 kg/h)

	C_3H_6 Purity	Distillation energy consumption MJ/kg (C_3H_6)	Catalytic hydrogenation energy consumption MJ/kg (C_3H_6)	Total energy consumption MJ/kg (C_3H_6)
1	99.9%	12.6 (120 trays)	0.33	12.9
2	99.9%	10.7 (140 trays)	0.33	11.0
3	99.9%	10.2 (160 trays)	0.33	10.5
4	99.99%	37.2 (120 trays)	0.33	37.5
5	99.99%	21.2 (140 trays)	0.33	21.5
6	99.99%	15.7 (160 trays)	0.33	16.0

Supplementary Table 13. Peak area of pure C_3H_6 on gas chromatogram at different flowrates.

C_3H_6 Flow Rate (mL/min)	Peak area of C_3H_6 on gas chromatogram (5 runs for each flow rate)					
	1	2	3	4	5	Average
**0.5**	242122655	241999436	243359982	242822446	242422136	242576129.8
**1**	402980681	402569858	402961636	402113722	402479468	402621073
**2**	791745545	791265404	792815638	791829422	792074543	791946110.4
**2.2**	875890769	875769510	876313521	877118717	878208307	876660164.8
**4**	1634797563	1627341600	1632185555	1626608924	1625901278	1629366984
**5**	2125749654	2123782849	2129630367	2127789707	2123854054	2126161326
**8**	3488385274	3489885376	3491554594	3496525947	3494715313	3492213301
**10**	4164949108	4158484708	4160955124	4175422954	4165375457	4165037470
**12**	5127780274	5126233395	5135672935	5137342795	5129699463	5131345772

Supplementary Table 14. The calculated C_3H_6 productivity based on the breakthrough curve of C_t/C_0 and F_t/F_0 under 298 K and 1.0 bar.

Mixtures	Material mass (g) Flow rate (mL/min)	Productivity calculated by breakthrough curve (L/kg)	Productivity calculated by breakthrough curve (L/kg)
		C_t/C_0	F_t/F_0
C_3H_4/C_3H_4(PD)/C_3H_8/C_3H_6 (1/1/3/95)	20.5 g, 5.0 mL/min	17.42 (99.99%)	16.95 (99.99%)
	20.5 g, 10.0	17.77 (99.99%)	17.27 (99.99%)
	1.33 g, 2.2 mL/min	16.30 (99.99%)	15.21 (99.99%)
C_3H_4/C_3H_4(PD)/C_3H_8/C_3H_6 (25/25/25/25)	2.28 g, 2.2 mL/min	3.61 (99.5%)	3.63 (99.5%)
C_3H_8/C_3H_6 (50/50)	1.33 g, 2.2 mL/min	11.08 (99.9%)	10.08 (99.9%)
