# Supplementary material for: One-step propylene purification from a quaternary mixture by a single physisorbent
Source: Nat Commun. 2025 Dec 13;16:11316. doi: 10.1038/s41467-025-66438-9 (PMC12722248; doi:10.1038/s41467-025-66438-9)
Supplement: Supplementary file 3 — Author Checklist [file 41467_2025_66438_MOESM3_ESM.docx]

| 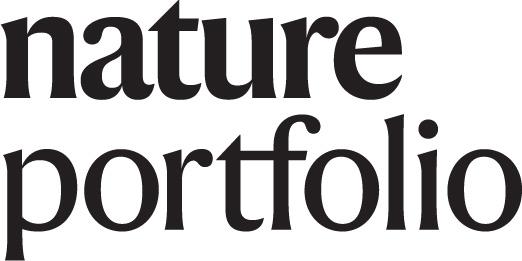 |  |
| --- | --- |

| **Author Checklist** | Manuscript Number: NCOMMS-25-66435 |
| --- | --- |
| **Please check the items below carefully and add a response in each row of the table to indicate the changes that you have made. Please also check through any additional marked-up edits we may have provided within the manuscript file.** | |
|  |  |
| **Author information** |  |
| Our guidance: | Your response: |
| We ask that you consult with your coauthors to ensure that all names, affiliations, and titles are represented correctly. Note that if any authors are added or removed after this point then all authors will be requested to provide approval documentation that could potentially delay the production of your paper. | We have confirmed all the authors and updated their affiliations. |
| Ensure affiliations are appropriately labeled and featured sequentially and in ascending order (1,2,3,... or a,b,c...). Please ensure all corresponding authors are marked with a specific symbol and include their emails. Similarly, if you have “equally contributing” or “joint supervision” authors, use a specific symbol to mark them and not a number. | We have ensured that all affiliations and corresponding authors are appropriately labeled. |
| Please ensure the author contributions section mentions each author's initials at least once with their contributions to the work. Authors with the same initials must be differentiated in the statement. | We have checked that each author’s initials and contributions are clearly listed and differentiated. |
|  |  |
| **Article structure** |  |
| Our guidance: | Your response: |
| We can accommodate up to 10 display items (Figures or Tables) in the main article. Each Figure and Table must fit easily within an A4 page (210 x 297 mm). Please ensure that the number and size of your Figures and Tables fulfil these requirements to avoid any delay in the acceptance of your article. | We have checked and made sure of the  number and size of my figures and  tables fulfil these requirements. |
| Ensure main Figures are uploaded as separate individual files. Each figure file must contain all intended panels labelled and displayed as intended and fit entirely on a single page. Do NOT include legends within the figure files, as these must be in the main manuscript.  Supplementary Figures must be all contained in the Supplementary Information PDF and do NOT need to be uploaded separately. | Each figure in main manuscript was uploaded as separate individual files. |
| **To comply with this format and optimise the presentation of data in your Article, we suggest the following changes to the display items in your paper:** |  |
| When scaled to fit our article template, text and detail in your figures may be too small to be legible; this includes text/labels in figures, and axis labels. Please provide your final figures at article scale and as a guideline, ensure that text is not smaller than 6 pts. | We have updated the figures and ensure all text is at least 6 pts for legibility. |
| **Please ensure your main manuscript file includes the following sections, in this order:** | We have revised the manuscript file  according to the formatting instructions |
| *Title Author list Affiliations Abstract Introduction Results Discussion (optional) Results and Discussion (optional) Methods Data Availability Code Availability (if relevant) References Acknowledgements Author Contributions Statement Competing Interests Statement Tables* *Figure Legends/Captions (for main text figures)* |  |
| We do not edit Supplementary Information files; they will be uploaded with the published article as they are submitted with the final version of your manuscript. Any tracked changes should be removed from the file and the file should be provided as a PDF file. Supplementary Figures do not need to be provided separately.  Please change the header of the Supplementary Information file to “Supplementary Information”. The title page of the Supplementary Information file should contain the header “Supplementary Information” followed by the title of the paper and authors’ names, or the first author’s name in the form “Smith et al”. | We have checked and made sure that the tracked changes have been removed from the file, and the file has been provided as a PDF file. |
| Please supply Source Data files for all data presented in graphs within the Figures. We are aware there are tools that can extract values from images, but these hinder both accessibility and accuracy of the extracted data, therefore we strongly advise authors to also provide the underlying data for all plots/graphs in the manuscript and supplementary information. | We have provided Source Data files for all data presented in the graphs to ensure accessibility and accuracy. |
| Within the file, the relevant raw data from each figure or table (in the main manuscript and in the Supplementary Information) should be represented by a single sheet in an Excel document, or a single .txt file or other file type in a zipped folder. An example of the Source Data file is available demonstrating the correct format: https://www.nature.com/documents/ncomms-example-source-data.xlsx The file should be labelled 'Source Data', with the title and a brief description included in your response here, and should be mentioned in all relevant figure legends using the template text below: 'Source data are provided as a Source Data file.' A reference to the source data file should be added in the 'Data Availability' section, using the text: Source data are provided with this paper. | We have completed this Source Data section as required. |
|  |  |
| **Main text** |  |
| Our guidance: | Your response: |
| Please do not use italics, bold font, underlining or speech marks/quotation marks except in headings unless required for technical terms (in both the main text and the display items). | OK, we have followed this rule. |
| Please make sure that mathematical terms throughout your manuscript and Supplementary Information (including in figures, figure axes, and legends) conform strictly to the following guidelines. Equations must be supplied in editable format, and not as images. Scalar variables (e.g. x, V, χ) must be typeset in italic, whereas multi-letter variables and functions (e.g. log) must be formatted in roman. Vectors (such as the wavevector k or the magnetic field vector B) must be typeset in bold without italics. | We have checked and made sure that　mathematical terms throughout the manuscript and supplementary information conform strictly to the　guidelines. |
| Please label equations sequentially as (1), (2), (3), etc. | We have labeled equations sequentially  as (1),(2),(3),(4)and(5). |
| Please refrain from using words such as new/novel/first/unique, when referring to the scientific findings as novelty can be inferred by context. Please also avoid exaggerated and subjective language and claims such as ‘extreme’, 'extremely', ‘giant’, ‘ultra’, ‘impressive’, 'outstanding', 'fascinating', ‘tremendous’, ‘powerful’, 'pave the way', ‘strikingly’, ‘ultimate’, ‘surprisingly’, ‘remarkable’, ‘notably’, ‘record’, ‘surprising’, 'unprecedented', ‘open new avenues’, ‘open the window’, ‘next generation’, and similar terminology. | We have checked and ensured that we have not used exaggerated or managerial words. |
| Please define abbreviations only once and at the first use. | We have checked. |
|  |  |
| **Figures and Tables** |  |
| Our guidance: | Your response: |
| Please see the guidelines linked below for detailed instructions about how your figures should be prepared. Following these instructions will reduce the chances of delays should we need to request replacement artwork from you at a later stage. | Thanks， we have followed the guidelines. |
| <https://www.nature.com/documents/NRJs-guide-to-preparing-final-artwork.pdf> |  |
| To ensure that your Figures are widely accessible, please make sure that the colour schemes used do not impair their readability. Please see the following resources for further information and suggestions:  Colour Brewer: http://colorbrewer2.org/#type=sequential&scheme=BuGn&n=3  Vischeck: http://www.vischeck.com/ Better Figures: https://betterfigures.org/2015/06/23/picking-a-colour-scale-for-scientific-graphics/ | Thanks, we have updated the colour schemes as suggested. |
| Using rainbow colour plots can be visually misleading (see links below) and I suggest altering the colour schemes used in your plots.  https://www.nature.com/articles/s41467-020-19160-7 https://www.climate-lab-book.ac.uk/2014/end-of-the-rainbow/ https://root.cern.ch/rainbow-color-map <https://eagereyes.org/basics/rainbow-color-map> | Thanks, we have changed our rainbow colour as suggested. |
| Red/green color contrasts can confuse our colorblind readers; please consider recoloring such figures, if possible. | Thanks, we have recoloured the figure and confirmed no Red/green colour contrasts in all figures. |
| Please note that schemes are not used; sequences of chemical reactions or experimental procedures should be submitted as figures, with appropriate captions. | We have revised the Scheme 1 into Figure 1. |
| All figure legends must include a brief title that summarises the whole figure. | We have added a brief title for all figures. |
| Please make sure that the terms ‘atomic units (a. u.)’ or ‘arbitrary units (arb. units)’ are appropriately used. | We have revised and confirmed it. |
| Any abbreviations, symbols or colours present in your figures must be defined in the associated legends. | OK, we have checked all the definitions in all legends. |
| The figure legends should contain enough information so that the meaning of the figure can be understood before reading the entire paper. Figures must also be accompanied by a brief title that summarises the whole figure and does not refer to specific panels. Please note that figure legends can be up to 350 words, and all definitions and abbreviations used in figures or figure legends should be defined. | We have checked all these as requested. |
| Please use a consistent Sans-Serif font and font size throughout all figures. | We have followed this rule. |
| Figure 2d: please define the different colours and the shape of the data points. | Our original definitions were placed above the figure; we have revised them to make them clearer. |
| Figure 2d: please define the blue/grey shaded region. | The blue/grey shaded region is for emphasis, and we have deleted this. |
|  |  |
| **Data and Code** |  |
| Our guidance: | Your response: |
| Nature journals strongly support public availability of data and code. Please deposit the data and code used in your paper into a public data repository, or alternatively, present the data as Supplementary Information. If data can only be shared on request, please explain why in your Data Availability Statement, and also in the correspondence with your editor.   Please note that for some data types, deposition in a public repository is mandatory. Any restrictions on sharing of these data types must be clearly indicated in the statement and discussed with the editor. More information on our data deposition policies and available repositories can be found here: | We have presented the data as Supplementary Information and crystallographic data are deposited at the Cambridge Crystallographic Data Centre. |
| <https://www.nature.com/nature-research/editorial-policies/reporting-standards#availability-of-data> |  |
| Please use the following template to provide all the information stated above:  The XX data generated in this study have been deposited in the YY database under accession code ZZ [add hyperlink here]. The XX data are available under restricted access for {insert reason}, access can be obtained by {explain how}. The raw XX data are protected and are not available due to data privacy laws. The processed XX data are available at YY. The XX data generated in this study are provided in the Supplementary Information/Source Data file. The XX data used in this study are available in the YY database under accession code ZZ [Add hyperlink here]. | We have revised the data availability section as requested. |
|  |  |
| **Methods** |  |
| Our guidance: | Your response: |
| Sufficient details of the experiments must be provided in the Methods section such that they could be reproduced without reference to published papers. Use of the term "as described previously" is not encouraged. | We have provided sufficient details of the experiments in the Methods section. |
| Please ensure that all accession codes used in this study (new ones AND previously published ones) are listed in the Data Availability statement, together with their corresponding hyperlink. Use precisely the following format to ensure that the links are permanent:  CODE [hyperlink] (description if necessary)  Use full DOI hyperlinks [http://doi.org/xxxxx] whenever possible.  For example:  5XRN [http://doi.org/10.2210/pdb5XRN/pdb]  1483958 [https://doi.org/10.5517/ccdc.csd.cc1lt5m6]  SRP109982 [https://www.ncbi.nlm.nih.gov/sra/?term=SRP109982]  GSE101099 [https://www.ncbi.nlm.nih.gov/geo/query/acc.cgi?acc=GSE101099]  NQLW00000000 [https://www.ncbi.nlm.nih.gov/assembly/GCA_002312845.1/]  PXD016640 [http://proteomecentral.proteomexchange.org/cgi/GetDataset?ID=PXD016640]  EMD-10857 [https://www.ebi.ac.uk/pdbe/entry/emdb/EMD-10857]  BMRB 28095 [https://dx.doi.org/10.13018/BMR28095] | In our article, no code was applied. |
|  |  |
| **End matter** |  |
| Our guidance: | Your response: |
| Nature Portfolio defines Competing Interest (CI) as financial and non-financial interests (including but not limited to funding, employment, stocks, shares, patents, personal or professional relationships with individuals or institutions, and unpaid membership advocacy) that could be perceived to directly undermine the objectivity, integrity, and value of a publication, or could be seen as having an influence on the judgments and actions of authors with regard to objective data presentation, analysis, and interpretation.  Please thoroughly review our policy on Competing Interests and include a detailed statement both in your final manuscript file and in our manuscript tracking system. Please ensure the statements are identical in both. Be specific about how each point stated relates to the research and list applicable author initials, and/or patent numbers.  If there are no competing interests, a negative statement must be included. | We have thoroughly reviewed the policy on Competing Interests and made sure the detailed statements are in both the final manuscript file and in the manuscript tracking system. |
| <https://www.nature.com/nature-research/editorial-policies/competing-interests> |  |
| Please confirm that all relevant funding awarded to each author is described in the Acknowledgements section. List each grant number, followed by the initials of the author who received it. | We have checked to make sure all relevant funding is awarded to each author in the Acknowledgements section. |
|  |  |
| **Preparing your manuscript files** |  |
| Our guidance: | Your response: |
| Unless otherwise stated please limit individual file sizes to approximately 30MB. We strongly encourage the use of repositories for large datasets or source data due to size considerations. | OK, the sizes of our files are lower than 30MB. |
| Please supply a brief (maximum 250 characters, including spaces) summary of the main findings of the paper to be used on our website and in our e-alerts. The summary should be written in the third person in language suitable for a broad audience. The summary may be edited by the editors prior to publication. Please provide this summary in your cover letter. | We have provided the summary in the cover letter. |
| Please provide figures as individual vector files with editable text. Acceptable file types for figures are .ai, .eps, .pdf, .ppt or Chem Draw for fully editable vector-based art. For detailed guidance on figure preparation, see https://www.nature.com/documents/aj-artworkguidelines.pdf | We provided figure files in PDF type. |
| Please note that all Supplementary Information must be provided as a single separate PDF file, not within the manuscript file.  All Supplementary Information items (e.g. Supplementary Figures, Supplementary Tables, Supplementary Methods, Supplementary Notes, Supplementary Discussion, Supplementary References) must be included in one PDF document. Please refer to our formatting guide when preparing your supplementary information file: https://www.nature.com/documents/ncomms-formatting-instructions.pdf   All Supplementary Information files (e.g. Supplementary Data, Supplementary Software, etc.) must be cited in the main text.  Every Supplementary Figure must be accompanied by a legend of up to 350 words, referring to all panels, and a brief title that summarises the whole figure.  Only Supplementary Movie, Audio, Data and Software files should be submitted separately from the Supplementary Information. | We have provided supplementary materials in PDF format and according to the required information format. |
| The use or adaptation of previously published images is strongly discouraged. If this is unavoidable, please request the necessary rights documentation to re-use such material from the relevant copyright holders and return this to us when you submit your revised manuscript. Please check whether your manuscript or Supplementary Information contain third-party images, such as figures from the literature, stock photos, clip art or commercial satellite and map data.  If any elements of your submitted work have been created with BioRender you will need to ensure you have obtained a publication license from BioRender, adhering to the user requirements as outlined within the license. The reference for BioRender created graphics should be present in the accompanying legend of the display material it is present in.  A copy of the publication license should be uploaded to our system as a related manuscript file upon resubmission.  For more information please see the BioRender knowledge article here: https://help.biorender.com/hc/en-gb/articles/21283116932765-CC-BY-publishing-and-reader-permissions  For more information on what constitutes ownership by a third party, please contact our Editorial Assistant at naturecommunications@nature.com | We have checked and confirmed that there are no third-party pictures. And scheme 1b was drawn by us. |
|  |  |
| **Please note that suspected third party content is present in scheme 1b.** |  |
|  |  |
| **You will need to upload:** |  |
| Completed Third Party Rights Table (if relevant) | No relevant |
| A point-by-point response to the reviewers' comments | Provided |
| A completed copy of this checklist | Provided |
| The main manuscript file in either Microsoft Word or LaTeX format | Provided Microsoft Word format |
| Separate Figure files | Provided |
| Separate Source Data files | Provided |
| Inventory of Supporting Information | Provided |
